# Phylogenetic analysis of the tenascin gene family: evidence of origin early in the chordate lineage

**DOI:** 10.1186/1471-2148-6-60

**Published:** 2006-08-07

**Authors:** RP Tucker, K Drabikowski, JF Hess, J Ferralli, R Chiquet-Ehrismann, JC Adams

**Affiliations:** 1 Department of Cell Biology and Human Anatomy, University of California at Davis, Davis, CA 95616, USA; 2 Friedrich Miescher Institute, Novartis Research Foundation, Basel, Switzerland; 3 Dept. of Cell Biology, Lerner Research Institute and Dept. of Molecular Medicine, Cleveland Clinic Lerner College of Medicine, Cleveland Clinic Foundation, Cleveland, OH 44118, USA; 4 Institute of Biology 3, University of Freiburg, Freiburg, Germany

## Abstract

**Background:**

Tenascins are a family of glycoproteins found primarily in the extracellular matrix of embryos where they help to regulate cell proliferation, adhesion and migration. In order to learn more about their origins and relationships to each other, as well as to clarify the nomenclature used to describe them, the tenascin genes of the urochordate *Ciona intestinalis*, the pufferfish *Tetraodon nigroviridis *and *Takifugu rubripes *and the frog *Xenopus tropicalis *were identified and their gene organization and predicted protein products compared with the previously characterized tenascins of amniotes.

**Results:**

A single tenascin gene was identified in the genome of *C. intestinalis *that encodes a polypeptide with domain features common to all vertebrate tenascins. Both pufferfish genomes encode five tenascin genes: two tenascin-C paralogs, a tenascin-R with domain organization identical to mammalian and avian tenascin-R, a small tenascin-X with previously undescribed GK repeats, and a tenascin-W. Four tenascin genes corresponding to tenascin-C, tenascin-R, tenascin-X and tenascin-W were also identified in the *X. tropicalis *genome. Multiple sequence alignment reveals that differences in the size of tenascin-W from various vertebrate classes can be explained by duplications of specific fibronectin type III domains. The duplicated domains are encoded on single exons and contain putative integrin-binding motifs. A phylogenetic tree based on the predicted amino acid sequences of the fibrinogen-related domains demonstrates that tenascin-C and tenascin-R are the most closely related vertebrate tenascins, with the most conserved repeat and domain organization. Taking all lines of evidence together, the data show that the tenascins referred to as tenascin-Y and tenascin-N are actually members of the tenascin-X and tenascin-W gene families, respectively.

**Conclusion:**

The presence of a tenascin gene in urochordates but not other invertebrate phyla suggests that tenascins may be specific to chordates. Later genomic duplication events led to the appearance of four family members in vertebrates: tenascin-C, tenascin-R, tenascin-W and tenascin-X.

## Background

Tenascins are a family of extracellular matrix glycoproteins charcterized by an N-terminal globular domain and heptad repeats, which facilitate multimerization; one or more tenascin-type epidermal growth factor (EGF)-like repeats (consensus sequence X_4_CX_3_CX_5_CX_4_CXCX_8_C); a series of fibronectin (FN) type III domains, and a C-terminal fibrinogen-related domain (FReD). Diversity within the family exists at many levels. Each species of vertebrate examined to date has more than one tenascin gene, and the gene products themselves are frequently alternatively spliced (e.g., see [1,2]). In addition, electron microscopy reveals purified tenascins with 6 arms (hexabrachions) as well as trimers, dimers and monomers [3,4]. Tenascins are particularly abundant in the embryonic extracellular matrix, but some reappear in the adult during regeneration, inflammatory disease, tumorigenesis and wound healing [2,5,6]. Tenascins act through interactions with cell surface receptors (reviewed by [4]; see also [7]) as well as by binding to and blocking sites on other extracellular matrix molecules (e.g., see [8]).

**Figure 1:**
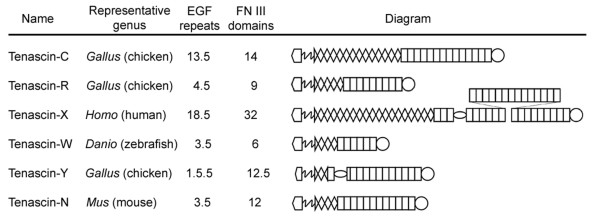
**The tenascins**. Six tenascins have been described in the literature: tenascins-C, -R, -X, -W, -Y and -N. This figure shows the repeat and domain organization of a tenascin that is representative of the group belonging to the genus where it was first described. The shapes found in the diagrams at the right symbolize the N-terminal linker domain (home plate), heptad repeats (zig-zag), EGF-like repeats (diamonds and partial diamonds), FN type III domains (rectangles), and a C-terminal FReD (circle). The serine/proline-rich domains of tenascin-X and tenascin-Y are indicated by an oval.

There are 6 names for tenascin gene products found in the current literature (Figure 1). Tenascin-C, the first tenascin to be cloned and sequenced [9-11], has 13.5 (chicken) or 14.5 (mammals) EGF-like repeats and up to 15 FN type III domains. The prominent expression of tenascin-C in tendons and embryonic extracellular matrix was used to create the name for the gene family, which comes from *tenere *(to hold) and *nasci *(to be born; see [12]). Tenascin-R was the second member of the tenascin family to be identified [13]. In birds and mammals, tenascin-R genes encode 4.5 EGF-like repeats and 9 FN type III domains. Tenascin-X is the name given to a large mammalian tenascin first identified as "gene X" in the major histocompatibility complex (MHC) class III gene region in both mouse and human [14,15]. This tenascin has 18.5 EGF-like repeats, and the tenascin-X genes of mouse and human encode 29 and 32 FN type III domains, respectively. The series of FN type III domains are interrupted in both mouse and human tenascin-X by a proline-rich stretch of about 100 amino acids. Tenascin-Y [16] is an avian tenascin described as being most similar to mammalian tenascin-X (the name "Y" comes from being "almost X"). The justification for a different name was because the similarity between the FReD of tenascin-Y and human tenascin-X was considerably lower than that between species orthologs of tenascin-C or tenascin-R. As in tenascin-X, the FN type III domains of tenascin-Y are interrupted by a region containing numerous serine-proline motifs. A fifth tenascin was eponymously named tenascin-W by Weber et al. [17]. Tenascin-W from both zebrafish [17] and mouse [18] has 3.5 EGF-like repeats, but a *Danio rerio *tenascin-W cDNA encodes five FN type III domains, whereas a murine cDNA encodes 9. The most recent tenascin to be described is tenascin-N [19], which like tenascin-W was named for its discoverer. Only characterized in the mouse, tenascin-N is identical to murine tenascin-W except for three additional FN type III domains (i.e., a total of 12 domains).

No tenascins have been identified in the *Caenorhabditis elegans *genome or in arthropod genomes, and the only complete cDNA sequences from poikilotherms come from *D. rerio *[17,20,21]. This has limited the phylogenetic analysis of the origins of tenascins, the identification of evolutionarily conserved regions, domains and potential receptor recognition motifs, as well as the ability to clarify the relationships between the different members of the tenascin gene family. Recently the genomes of a variety of organisms have been completely, or nearly completely, sequenced, facilitating the identification of tenascin genes in fish, amphibians, and invertebrate chordates. Using searches based on conservation of amino acid sequences and domain architecture, we have identified in silico the putative tenascin gene products of the freshwater green pufferfish, *Tetraodon nigroviridis*, the Japanese tiger pufferfish, *Takifugu rubripes*, the pipid frog, *Xenopus tropicalis*, and the ascidian, *Ciona intestinalis*. These studies show that the basic domain architecture of tenascins is highly conserved between an invertebrate chordate and vertebrates and that there are only four members of the tenascin family in vertebrates: tenascin-C, tenascin-R, tenascin-W and tenascin-X.

## Results

### *Ciona intestinalis *tenascin

**Figure 2:**
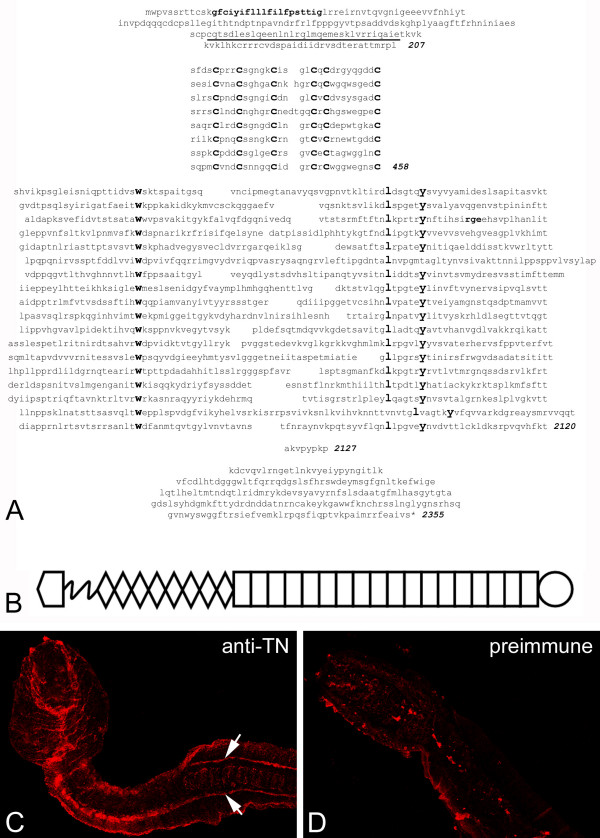
**Ciona *intestinalis *tenascin**. **A**. The amino acid sequence of a tenascin from *C. intestinalis*. The N-terminal linker region is at the top, with a signal peptide shown in bold and putative heptad repeats underlined. Between amino acids 208 and 458 are 8 EGF-like repeats. These are followed by 18 FN type III domains between amino acids 459 and 2120. The tryptophan (w), leucine (l) and tyrosine (y) residues that are characteristic of these domains are highlighted and aligned, and a putative integrin-binding motif (rge) found in the third FN type III domain is shown in bold. The C-terminal FReD is composed of amino acids 2128 through 2355. **B**. The repeat and domain organization of the *C. intestinalis *tenascin shown in A. A key to the shapes symbolizing each domain can be found in the legend to Figure 1. **C**. A rabbit antiserum against a recombinant fragment of *C. intestinalis *tenascin was used to immunostain whole larvae. The antiserum recognized the tunic, a line of matrix in the tail (arrows), and faintly labelled the tail muscles (between the arrows). **D**. The rabbit preimmune serum inconsistently labelled the tunic but not the line of matrix in tail or the tail muscles.

**Figure 3:**
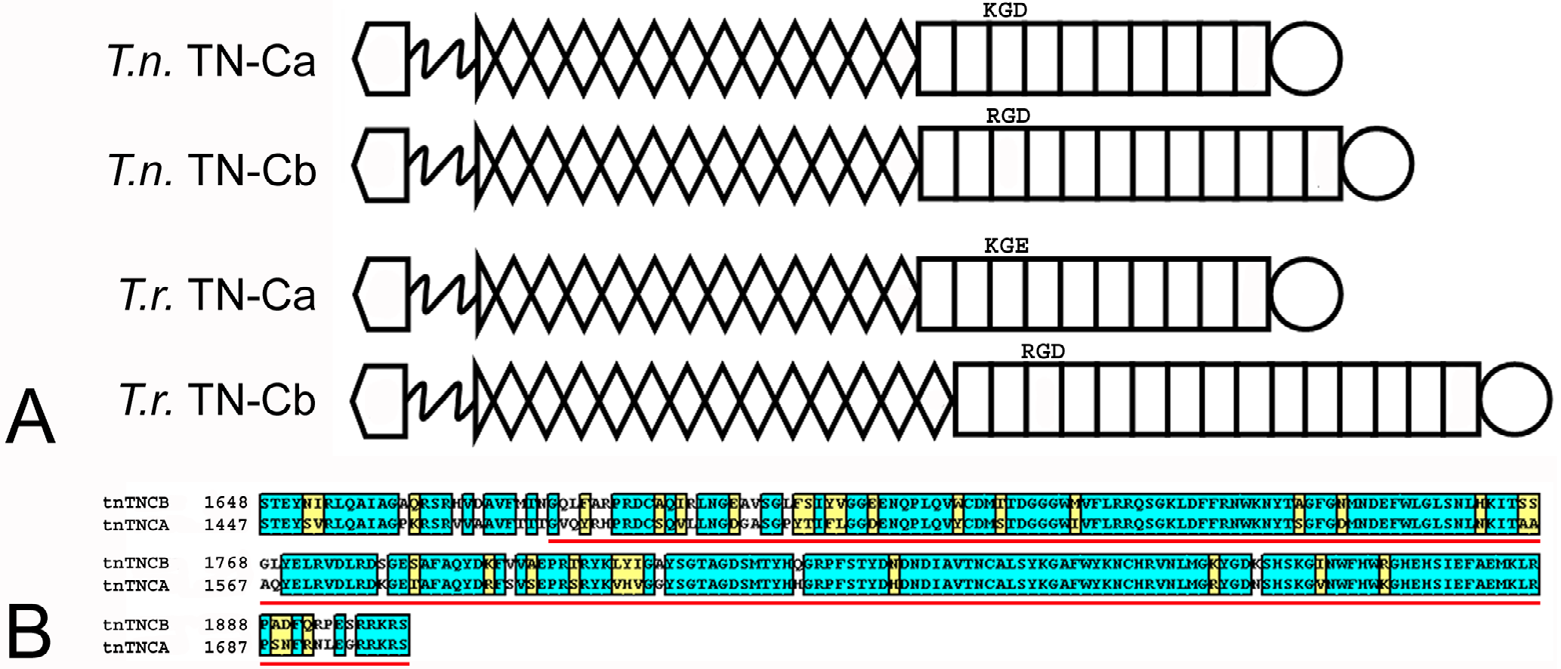
**There are two tenascin-Cs in pufferfish**. **A**. Analysis of the genomic sequences of *Tetraodon nigroviridis *(*T.n*.) and *Takifugu rubripes *(*T.r*.) reveals that each species of pufferfish had two tenascin-C genes. The repeat and domain organization of the paralogous genes are illustrated here. All four have a putative integrin binding motifs (kgd, rgd or kge) in the third FN type III domain. **B**. The C-terminal FReDs (underlined in red) of the two tenascin-Cs from *Tetraodon nigroviridis *are highly conserved. Identical residues are boxed in blue and similar residues are boxed in yellow.

Analysis of the genomic sequence of the urochordate *C. intestinalis *[22] using the BLAT [23] and SNAP (UCSC Genome Bioinformatics [24]) [25] programs reveals a single tenascin-like gene product. It was necessary to modify this predicted protein by visual inspection of the genomic sequence to remove some parts of predicted exons that did not show any homology to the known tenascins and by the identification of open reading frames complementing missing parts of three of the fibronectin type III repeats. The sequence of the N-terminus was confirmed by predicting a potential signal peptide upstream of the EGF repeats followed by RT-PCR using primers homologous to the region harboring the methionine N-terminal to the predicted signal peptide and a primer from the previously predicted region. The coding part of this tenascin gene lies between position 1585–21365 of scaffold 366 that is located on chromosome 9 (Laboratory for Developmental Biology and Genome Biology [26]; see [27]). The domain organization predicted by SMART [26] reveals an N-terminal signal sequence (aa 13–32), 4 and 1/2 heptad repeats (aa 136–168; confirmed by Paircoil [see Methods]), 8 tenascin-type EGF-like repeats (aa 208–458), 18 FN type III domains (aa 469–2119) and a FReD (2128–2355). The C-terminal portion of the predicted protein is confirmed by an EST [GenBank EST:BW395090.1] that includes the FReD, as well as by the 9 ESTs in Gene Cluster 02393 of the cDNA resources of the Ghost Database [29]. In addition, a highly-related gene product is encoded on *C. savigyni *contig 41574 [GenBank CoreNucleotide:AACT01041574]. The corrected *C. intestinalis *protein sequence has 2355 aa (Figure 2A), a predicted pI of 7.63 and a molecular mass of 262 kDa. A stick diagram of this tenascin, the first reported from an invertebrate, is shown in Figure 2B. Note that the third FN type III domain of *C. intestinalis *tenascin contains the tripeptide motif RGE in a region that is likely to be exposed to receptor binding [30] and that corresponds to the position of the integrin-binding RGD motifs of tenascin-C in many vertebrates (e.g., see Figure 3A). As others have shown that the RGE motif can have similar properties to the RGD motif [31], this may represent an integrin-binding region in tenascin from *C. intestinalis*.

To analyze the expression pattern of this novel tenascin we raised an antiserum against the second FN type III domain expressed in *E. coli *(see Methods). In whole mounts this antiserum recognized the thin tunic that envelopes the larva, the extracellular matrix at the base of the tail, and periodically arrayed structures found on either side of the nerve cord that may correspond to the dorsal portions of the primary muscles of the tail (Figure 2C). This latter staining pattern is reminiscent of the immunostaining of the sclerotome in chick and mouse embryos with antibodies against tenascin-C [32]. The tunics of the larvae incubated with preimmune serum were often labelled, but the matrix at the base of the tail and associated with the tail musculature was not (Figure 2D).

The genome sequencing of another urochordate, *Oikopleura dioica*, has been completed (Trace Archive Database Mega BLAST [33]). Using the Hidden Markov Model on the alignments of all *O. dioica *FReDs we could identify a putative tenascin gene in this distinct urochordate as well. Unfortunately, we were not able to identify the entire *O. dioica *tenascin gene because the genome scaffolds have not been assembled yet (data not shown).

### *Tetraodon *and *Takifugu *each have five tenascins

Using a BLASTP [34] search of predicted proteins from all translated sequences with the chicken tenascin-C FReD (selected because it generated fewer non-tenascin hits than searches with other repeats or domains), four potential tenascin-like sequences were identified in the freshwater pufferfish *Tetraodon nigroviridis *(see Table 1 for NCBI Protein Database accession numbers [see Additional file: 1]). These translated sequences were then entered into the BLAT field of the *T. nigroviridis *Gene Browser at Genoscope, which returned four predicted tenascin-like proteins: 1) Genoscope identifier GSTENT00020055001, chrUn_random:20095278..20099581; 2) Genoscope identifier GSTENT00025206001, chrUn_random:1836985..1839543; 3) Genoscope identifiers GSTENT00028393001, chr15:5874301..5884063 and GSTENP00028394001, chr15:5848022..5850949; and 4) Genoscope identifier GSTENT00028391001; chr15:5898778..5899089. A fifth potential tenascin was identified using a BLAT search of the *T. nigroviridis *genome using the FReD of a tenascin identified by others [35] in the Japanese pufferfish *Takifugu rubripes *(Genoscope identifier GSTENT00034161001, chr8:6275880..6277019). The *Tetraodon nigroviridis *tenascin sequences were then used to search the genome of the Japanese pufferfish *Takifugu rubripes *at the Fugu Genome Browser Gateway at UCSC Genome Bioinformatics and the NCBI Fugu Genome Project. These searches also revealed five putative tenascin genes: 1) Genescan predicted gene scaffold_2387.1 and SINFRUT00000156379; 2) Genescan predicted gene scaffold_148.12 and SINFRUT00000139096; 3) Genescan predicted gene scaffold_392.8 and SINFRUP00000066378; 4) Genescan predicted gene scaffold_392.6 and SINFRUP00000069989 and 5) Genescan predicted gene scaffold_795.4 and SINFRUP00000072635 [see Additional file: 2].

**Table 1:** Accession numbers of sequences used to obtain data for searching, alignment and phylogenetic reconstructions of tenascins*

Name	NCBI Protein
*H. sapiens *tenascin-C	CAA55309
*M. musculus *tenascin-C	NP_035737
*G. gallus *tenascin-C	NP_990787
*D. rerio *tenascin-C	CAA61489
*T. nigroviridis *tenascin-Ca	CAG01316
*T. nigroviridis *tenascin-Cb	CAG05242
*H. sapiens *tenascin-R	CAI21650
*M. musculus *tenascin-R	NP_071707
*G. gallus *tenascin-R	CAA45920
*D. rerio *tenascin-R	AAP37046
*T. nigroviridis *tenascin-R	CAG07653
*H. sapiens *tenascin-W	CAB41260
*M. musculus *tenascin-W	CAE45651; NP_808507
*G. gallus *tenascin-W	XP_422277
*D. rerio *tenascin-W	CAA04755
*T. nigroviridis *tenascin-W	CAG07652
*H. sapiens *tenascin-X	AAB41287
*M. musculus *tenascin-X	AAB82015
*Takifugu rubripes *tenascin-X	CAD45004
*G. gallus *tenascin-X ("Y")	CAA67509

*The accession numbers *Xenopus *tenascins from the *X. tropicalis *Genome Browser Gateway [24] the NCBI Frog Sequence Database [39] are listed separately in the text.

### Pufferfish have two tenascin-Cs

Two of the predicted tenascin sequences found in the *Tetraodon nigroviridis *genome showed the most similarity to zebrafish, chicken, and the numerous mammalian tenascin-Cs following BLASTP analyses of their FReDs and first and last FN type III domains. Both of these tenascins were predicted by SMART and visual inspection to include an N-terminal linker, a series of heptad repeats, 12.5 EGF-like repeats and a C-terminal FReD. One of the two sequences contains 12 FN type III domains (GSTENT00025206001) and the other contains 10 FN type III domains (GSTENT00020055001; Figure 3A; note that it was necessary for us to complete several predicted partial FN type III domains by identifying missing exons by translating nearby open reading frames in the genomic sequence). The amino acid sequences of the FReDs of the two tenascins share 80% identity and 94% similarity (Figure 3B). Bayesian inference of the phylogenetic trees predicts that these proteins are more closely related to each other than to any other vertebrate tenascin. Because of their sequence similarity to each other and to other tenascin-Cs, overall domain organization, and predicted evolutionary proximity, we will refer to these paralogs as tenascin-Ca (modified from GSTENT00020055001) and tenascin-Cb (modified from GSTENT00025206001). Like tenascin-C in *Gallus gallus *and man, tenascin-Cb contains an RGD integrin-binding motif in its third FN type III domain in a region predicted to be exposed to receptor binding. In tenascin-Ca this region contains the potentially reactive motif KGD. Similarly duplicated tenascin-C genes were identified in the *Takifugu rubripes *genome (Figure 3A). SMART reveals that one predicted protein (scaffold_2387.1 and SINFRUT00000156379) has heptad repeats, 12.5 tenascin-type EGF-like repeats, 10 FN type III domains and a C-terminal FReD. It was also necessary to modify this predicted protein by identifying open reading frames in the genomic sequence that corresponded to missing exons that completed several FN type III domains. The domain organization of the corrected tenascin is identical to that of *Tetraodon nigroviridis *tenascin-Ca. The FReDs of the two pufferfish tenascin-Cas are 95% identical and 99% similar. The second *Takifugu rubripes *tenascin-C (scaffold_148.12 and SINFRUT00000139096) is predicted by SMART and direct examination to have heptad repeats, 13.5 EGF-like repeats, 15 FN type III domains and a C-terminal FReD. Like *Tetraodon nigroviridis *tenascin-Cb, it has an RGD motif in the third FN type III domain (Figure 3A). The amino acid sequences of the tenascin-Cb FReDs from the two species of pufferfish are 92% identical and 98% similar.

### Tenascin-R in *Tetraodon *and *Takifugu*

There are two predicted protein sequences lying side-by-side on chromosome 15 of *T. nigroviridis *that, when considered as a single gene product, contain an N-terminal linker, heptad repeats, 3.5 EGF-like repeats, 7 FN type III domains and a C-terminal FReD (GSTENT00028393001 and GSTENP00028394001). Phylogenetic analysis of the FReD and terminal FN type III domain show this potential tenascin to be most similar to amniote and zebrafish tenascin-R. Like tenascin-R in *D. rerio *it is adjacent to, and in the opposite orientation from, a tenascin-W gene (see below). The *T. nigroviridis *tenascin-R sequence, however, is not complete; the genomic sequence found between the regions encoding the EGF-like repeats and FN type III domains contains approximately 500 undetermined nucleotides. In contrast, the *Takifugu rubripes *tenascin-R (SINFRUP00000066378), found by BLASTP of the NCBI Protein Database, is missing only the signal sequence at the N-terminus. The remaining predicted protein includes heptad repeats, 4.5 EGF-like repeats, 9 FN type III domains and a C-terminal FReD. Alignment of the two pufferfish tenascin-R sequences confirms that the fourth EGF-like repeat and first two FN type III domains of *Tetraodon nigroviridis *are missing from the predicted protein. The domain architecture of *Takifugu rubripes *tenascin-R is identical to that found in the predicted tenascin-R proteins encoded in genomes of *D. rerio*, *G. gallus*, mouse and man (e.g., see Figure 1). Pufferfish tenascin-R does not contain an RGD motif, but the third FN type III domains contain an IDG motif, which is a potential recognition site for the alpha4/alpha9 family of integrins [36]. This motif is also found in the same location on *D. rerio*, *G. gallus*, *M. musculus *and human tenascin-R.

### Unique repeats in pufferfish tenascin-X

**Figure 4:**
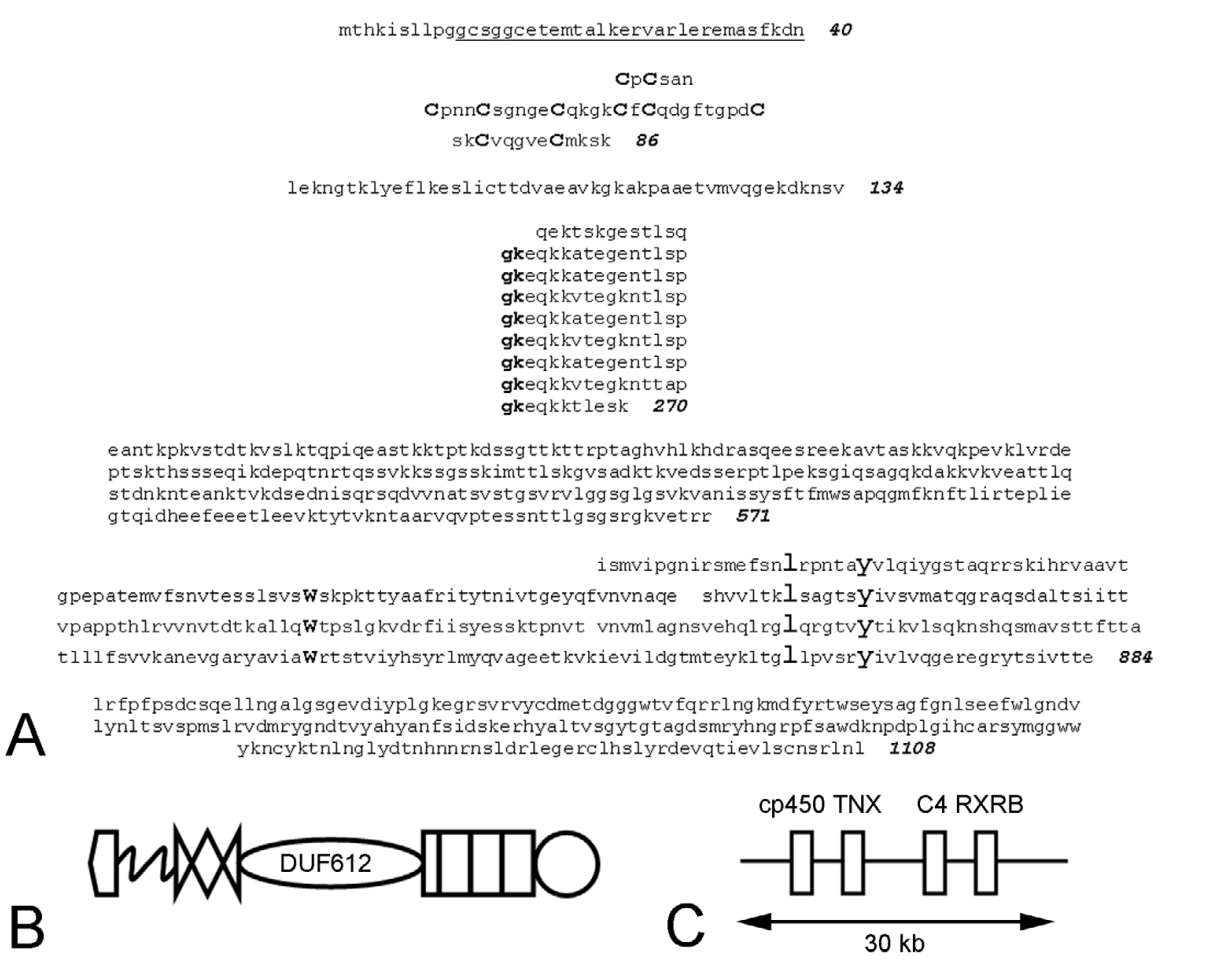
***Tetraodon nigroviridis *tenascin-X**. **A**. The predicted amino acid sequence of *T. nigroviridis *tenascin-X. Putative heptad repeats (underlined) are found near the N-terminus. Between amino acids 41 and 86 are one complete are two partial EGF-like repeats. This is followed by a short (48 amino acid) linking region and 7 complete and two partial GK repeats (between amino acids 135 and 270). Between the GK repeats and amino acid 571 is a region rich in charged amino acids. Three complete and one partial FN type III domains are found between amino acids 572 and 884. There is a FReD at the C-terminus. **B**. The repeat and domain organization of pufferfish tenascin-X. The region between amino acids 87 and 571 is similar to the DUF612 domain of UNC-89. **C**. The *T. nigroviridis *tenascin-X gene (TNX) is found between the genes encoding cytochrome p450 21-hydroxylase (cp450), C4 complement and retinoid X receptor beta (RXRB). The same genes overlap or flank tenascin-X genes in birds and mammals.

In addition to the BLASTP searches with the chicken FReD sequence (see above), a BLAT search of the *T. nigroviridis *genome was carried out using the FReD sequence of a partial putative tenascin-X identified by others [35] in *Takifugu rubripes *[NCBI Protein:CAD45004]. This revealed a novel tenascin with numerous features not yet described in the gene family (GSTENT00034161001). Note that it was necessary to identify an exon in the genomic sequence to derive the final amino acid sequence illustrated in Figure 4A. The putative *Tetraodon nigroviridis *tenascin-X is much smaller than mammalian tenascin-X: it encodes at most 1108 amino acids and is predicted to have a molecular mass of 122 kDa. The COILS program (see Methods) predicts that it has heptad repeats near the N-terminus (underlined in Figure 4A), and SMART reveals a single tenascin-type EGF-like sequence that, as in tenascin-Y, is followed by what may be considered a partial EGF-like repeat. Separating the EGF-like repeat from one partial and three complete FN type III domains is a stretch of 485 amino acids, many of which are charged or polar. The first 48 amino acids in this region contains numerous charged residues, but a BLASTP search fails to reveal any similarities between this region and any other sequence. The next 136 residues make up 8 complete and one partial copies of a previously undescribed simple tandem repeat of 16 amino acids (GKEQKKATEGENTLSP) that mostly contains polar or charged residues. BLASTP searches with these repeats fail to reveal significant homologies with proteins from vertebrates, but the region is 43% similar to a collagen-binding, surface protein from *Bacillus cereus *[NCBI Protein:YP_082380]. The final 301 residues of this region are also highly charged and, when examined by BLASTP on their own, fail to show homologies with other known proteins. However, when the entire predicted open reading frame of *T. nigroviridis *tenascin-X is used in a BLASTP run, the 16-residue repeats and the adjacent 301-residue region are recognized as a DUF612 domain (Pfam 04747; Figure 4B). The function of the DUF612 domain is unknown, but it is found in *C. elegans *UNC-89, a large multidomain protein required for myofibril assembly [37].

The unusual repeat and domain structure of *T. nigroviridis *tenascin-X is also seen in *Takifugu rubripes *tenascin-X. This was determined by a BLAT search of the *T. rubripes *genome with the FReD of *Tetraodon nigroviridis *tenascin-X. This search revealed a very large predicted protein (GENSCAN00000009040), which, upon further examination, was found to be composed of three closely clustered but distinctive gene products. The middle gene encodes a predicted tenascin with one EGF-like repeat, 19 charged repeats similar to those found in *T. nigroviridis *tenascin-X interrupted by two partial charged repeats, a charged domain that is 69% identical to amino acids 271–572 in *T. nigroviridis *tenascin-X, one partial and three complete FN type III domains, and a C-terminal FReD.

Phylogenetic analysis shows that the FReDs from the putative pufferfish tenascin-Xs are most similar to the FReDs of chicken tenascin-Y and mammalian tenascin-X (see below). However, the most striking evidence that the unusual tenascin-X of *T. nigroviridis *is indeed a member of this particular tenascin subfamily is its genomic location: it is found on chromosome 8 flanked by the genes encoding cytochrome p450 21-hydroxylase and C4 complement (Figure 4C). In mammals, the tenascin-X gene overlaps with the cytochrome p450 21-hydroxylase gene, which is encoded on the opposite strand of DNA, and lies adjacent to one of two C4 complement genes (e.g., see [15]). The retinoid X receptor beta gene, which lies next to the C4 gene in *T. nigroviridis*, is also an MHC complex gene in mammals found approximately 1 Mb from the human tenascin-X gene on chromosome 6. Thus, both by sequence homology and by synteny, the small gene encoding a single tenascin-type EGF-like sequence and only three complete FN type III domains corresponds to the *T. nigroviridis *ortholog of mammalian tenascin-X.

### Tenascin-W: diversity through domain duplication

A putative gene product with a repeat and domain structure similar to zebrafish tenascin-W was identified in *T. nigroviridis *(GSTENT00028391001). This tenascin gene encodes an N-terminal linker, heptad repeats, 3.5 EGF-like repeats, four FN type III domains, and a C-terminal FReD. BLASTP of the first FN type III domain, the fourth FN type III domain, and the FReD all reveal that this putative gene product is most similar to tenascin-W from zebrafish and mouse. A similar tenascin-W was found in the genome of *Takifugu rubripes *(SINFRUP00000069989), but this tenascin-W has five FN type III domains instead of four. There is an RGD motif in the fifth FN type III domain of *T. rubripes *tenascin-W, but this motif is not found in tenascin-W from *Tetraodon nigroviridis*.

**Figure 5:**
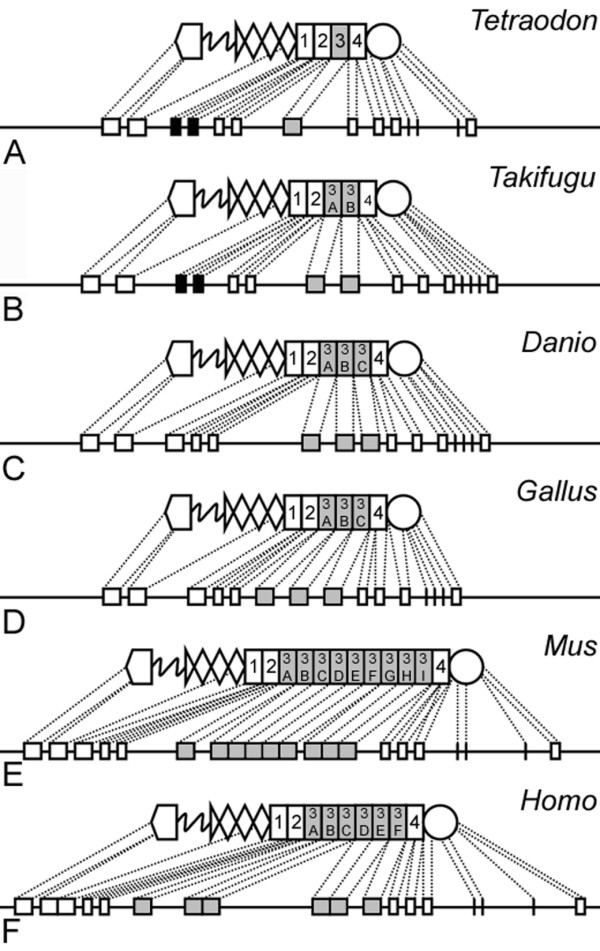
**Tenascin-W diversity is generated by duplications of a FN type III domain**. **A**. The tenascin-W of *Tetraodon nigroviridis *is predicted to be encoded on 14 exons. The figure shows a schematic of the predicted protein's repeat and domain organization and the corresponding exons. The N-terminal linker is encoded on the first exon. The second exon encodes the heptad repeats and the EGF-like repeats. This is conserved in all of the tenascin-Ws illustrated here. FN type III domains 1, 2 and 4 are encoded on two exons, but the third FN type III domain is encoded on a single exon (shaded). The FReDs of all of the tenascin-Ws is encoded on five exons. **B**. The full-length predicted tenascin-W of *Takifugu rubripes *has five FN type III domains. The additional domain is the result of a duplication of the third FN type III domain, which is encoded on a single exon. **C**, **D**. The predicted tenascin-Ws of *Danio rerio *(C) and *Gallus gallus *(D) have 6 FN type III domains, the apparent consequence of an additional duplication of the third FN type III domain. **E**, **F**. In mouse (*Mus*) and man (*Homo*) the very large tenascin-W predicted proteins can also be explained by multiple duplications of the third FN type III domain. Note that the first FN type III domains of the pufferfish, but not the other tenascin-Ws, are encoded on two exons (black). The relative sizes of the exons and introns between the different genera are not shown to scale.

**Figure 6:**
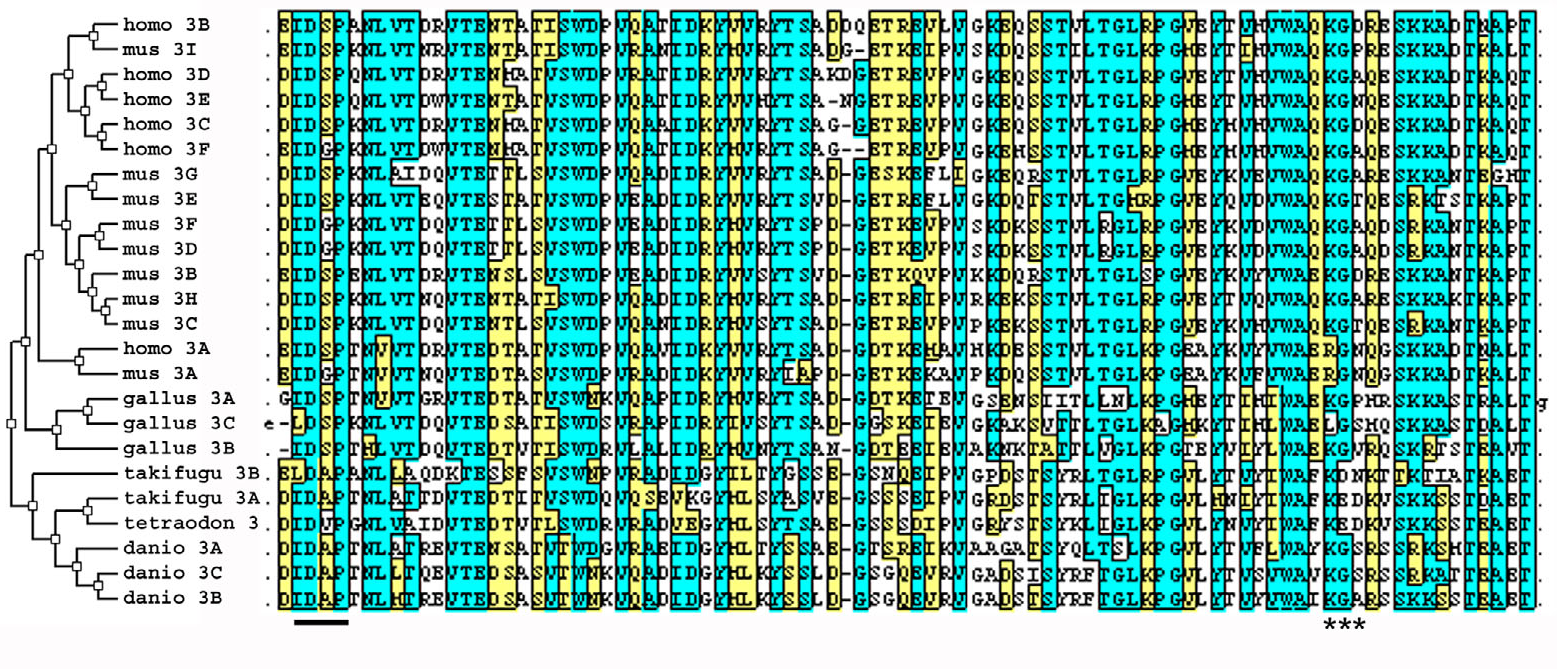
**Alignment of the duplicated FN type III domains oftenascin-W**. The third FN type III domain of *Tetraodon nigroviridis *tenascin-W has been duplicated one or more times in other tensacin-Ws. Alignment reveals the conservation of sequences within these domains, including putative integrin binding motifs near the N-terminus of the domain (underlined). The region where an integrin-binding RGD sequence in an exposed loop is found in chicken tenascin-C is indicated by asterisks. Several tenascin-Ws have a potentially active KGD motif in this region. At the left is a rooted phylogenetic tree generated by SATCHMO. This analysis indicates that many of the domain duplications took place after the divergence of primate and rodent lineages. Identical amino acids are shaded blue, while similar amino acids are boxed in yellow.

The predicted tenascin-W orthologs from *T. nigroviridis*, *Takifugu rubripes*, *D. rerio*, *G. gallus*, *M. musculus *and human all have an N-terminal linker, heptad repeats, 3.5 EGF-like repeats and a C-terminal FReD. They differ, however, in the numbers of FN type III domains encoded in their respective tenascin-W genes. The smallest number of domains is seen in *Tetraodon nigroviridis *(with four) and *Takifugu rubripes *(with five); *D. rerio *and chicken have 6, mouse has 12, and man has 9 FN type III domains (Figure 5). Despite the differences in numbers of FN type III domains, each of the tenascin-W genes listed above is found in each genome immediately adjacent to the tenascin-R gene and encoded in the opposite orientation. For example, in *Tetraodon nigroviridis *the two tenascin genes are separated by only 10 kb on chromosome 15, and in mouse the two tenascin genes are only 15 kb away from each other on chromosome 1. In *D. rerio*, the two genes are encoded on chromosome 2 (Wellcome Trust Sanger Institute, zebrafish genome assembly Zv5). Moreover, the different tenascin-W genes share nearly identical intron/exon organization in each species examined (Figure 5). In *D. rerio*, *G. gallus*, *M. musculus *and man the first tenascin-W FN type III domain is encoded on a single exon, the second FN type III domain is encoded on two exons, and the final FN type III domain, adjacent to the FReD, is encoded on two exons. The intron/exon junction sites are the same in each of these species. As in other tenascins, the heptad linkers and EGF-like repeats are encoded on a single exon, and the FReD is encoded by five exons. In the pufferfish, tenascin-W is identically encoded except for the first FN type III domain, which is formed from two exons (Figure 5). The variation in the numbers of FN type III domains between the various tenascin-W predicted proteins seems to be the result of repeated duplications of the third FN type III domain. There is only one of these domains in *T. nigroviridis*, and it is encoded by a single exon. In *Takifugu rubripes*, Sequence Alignment and Tree Construction using Hidden Markov mOdels (SATCHMO) analysis [38] reveals that the third FN type III domain has recently duplicated, resulting in a total of five FN type III domains. In *D. rerio *and *G. gallus *this domain has duplicated twice, resulting in the 6 total FN type III domains. Evidence of this duplication is provided by the very high sequence similarities between these particular domains, as well as from construction of a phylogenetic tree based on their sequences (Figure 6). This tree also reveals that the duplications most likely took place after the evolutionary separation of the species examined, since the descendents of the third FN type III domain are typically most similar within a species than between species. Note that the *D. rerio *tenascin-W cDNA sequence reported by Weber et al. [17] only contained five FN type III repeats. The extra repeat shown here (which corresponds to repeat 3C in Figures 5 and 6) was found in the genomic sequence where, like all of the duplicated repeats, it is predicted to be encoded from its own exon. The presence of this novel FN type III domain in tenascin-W transcripts was confirmed by RT-PCR using mRNA from an adult zebrafish as a source of cDNA template and primers corresponding to the end of the fourth FN type III domain and the beginning of the sixth FN type III domain (see Methods for details). In the mouse, the 12 FN type III domains are the result of an eight-fold replication of the third domain, and in man the third FN type III domain is replicated five times. Sequence similarities and phylogenetic analysis also reveal the possible order in which these duplications occurred (Figure 6). As one might expect, the species with the most third FN type III domains, the mouse, has some repeats that are very similar to each other: domains 3D and 3F have identical amino acid sequences, as previously noted by Neidhardt et al. [19].

### *Xenopus tropicalis *tenascins

**Figure 7:**
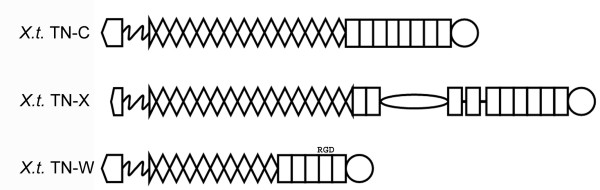
***Xenopus tropicalis *tenascins**. Four tenascins were identified in the *X. tropicalis *genome. Stick diagrams of the three complete sequences are shown here. The *X. tropicalis *tenascin-C gene encodes 14.5 EGF-like repeats and 8 FN type III domains. There are no RGD motifs. The domain represented by an oval between the second and third FN type III domains shares sequences similarities with the DUF612 domain of pufferfish tenascin-X and the SP-domain of avian and mammalian tenascin-X. The amphibian tenascin-W contains an RGD domain in an exposed loop in the fourth FN type III domain and a KGD motif in the fifth FN type III domain; these domains appear to have undergone a recent duplication.

Four tenascin genes were identified in the genome of the amphibian *Xenopus tropicalis *by BLAT searches of the *X. tropicalis *Genome Browser Gateway (UCSC Genome Bioinformatics [24]) and by BLASTP searches of the NCBI Frog Sequence Database (National Center for Biotechnology Information [39]) with the FReD sequences from chicken and pufferfish tenascins (Figure 7 [see Additional file: 3]). The tenascin-C predicted from the *X. tropicalis *genome (68086_prot), identified by the similarity of its FReD sequence with that of fish and amniote tenascin-Cs, has heptad repeats near its N-terminus, 14.5 EGF-like repeats, and 8 FN type III domains. It does not have an RGD or RGD-like motif in its FN type III domains. Only a partial *X. tropicalis *tenascin-R sequence is found in the database; it encodes a single FN type III domain and a FReD (JGI Filtered Gene 30381) at the terminus of scaffold 197. However, a complete tenascin-X was found by combining two predicted proteins (50684_prot and 63933_prot). When combined the single predicted protein contains heptad repeats, 14 complete and two partial EGF-like repeats, 10 FN type III domains and a C-terminal FReD. Between the second and third FN type III domains is a 649-amino acid region that is not recognized as a known domain by SMART. Near the center of this region is a 37-amino acid stretch that shares 70% identity and 89% similarity to part of the serine-proline-rich domain of tenascin-Y and 59% identity and 72% similarity to part of the proline-rich domain of human tenascin-X. The entire region is 38% similar to a portion of UNC-89, but the SMART program does not predict the presence of a DUF619 domain. The fourth tenascin found in the *X. tropicalis *genome is tenascin-W. It was identified by combining two predicted proteins (30377_prot and scaffold_197.67) using the C-terminal sequence of the former, since this was the most similar to tenascin FReDs following alignment. Thus assembled, the *X. tropicalis *tenascin-W gene encodes a signal sequence, heptad repeats, 9.5 EGF-like repeats, five FN type III domains and a C-terminal FReD. The fourth FN type III domain contains a potentially active RGD motif and the fifth contains a KGD motif also predicted to be in an exposed loop. Unlike the other tenascin-Ws described here (Figure 5), *X. tropicalis *tenascin-W has 9.5 EGF-like repeats instead of 3.5, and the fourth (not the third) FN type III domain has duplicated itself once.

### Analysis of phylogeny and synteny

**Figure 8:**
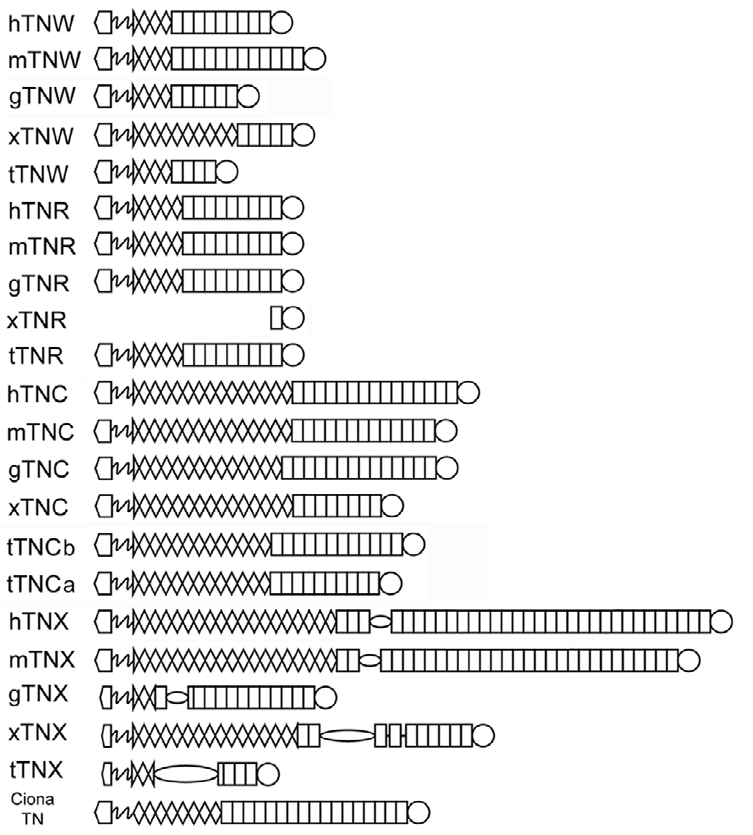
**Tenascins**. Stick diagrams illustrating the hypothetical repeat and domain organizations of tenascins based on genomic sequences. A key to the shapes used can be found in the legend to Figure 1. h, *Homo*; m, *Mus*; g, *Gallus*; t, *Tetraodon*; x, *Xenopus*.

**Figure 9:**
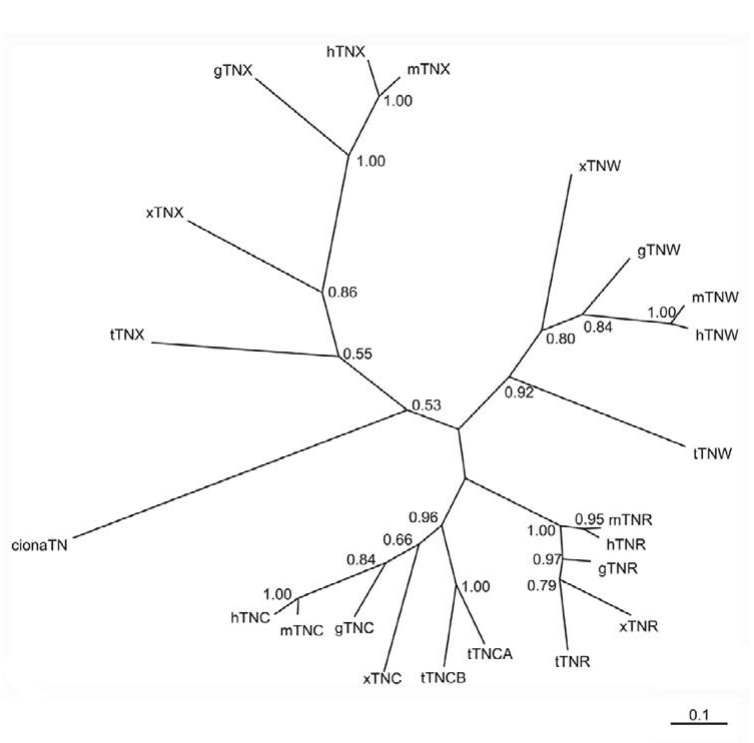
**A tenascin phylogenetic tree**. The amino acid sequences of the FReDs from urochordate, fish, amphibian and mammalian tenascins were used to construct an unrooted molecular phlyogenetic tree. The numbers at the internal nodes are the probability that each branch point is correct. The scale represents 0.100 expected changes. The tree reveals that there are four members of the tenascin family in vertebrates: tenascin-X, tenascin-W, tenascin-C and tenascin-R.

A summary of the repeat and domain organizations of the novel predicted tenascins and the previously described amniote tenascins is shown in Figure 8. Phylogenetic reconstruction using Bayesian inference based on the FReD of the tenascins of pufferfish, *X. tropicalis*, *G. gallus*, mouse and man reveals four members of the vertebrate tenascin gene family: tenascin-C, tenascin-R, tenascin-W and tenascin-X (Figure 9). Of these, tenascin-X represents the most distinctive sequence. A nearly identical tree based on the FReD sequences was constructed by maximal likelihood phylogeny (results not shown). As reported by Chiquet-Ehrismann et al. [40], Erickson [41], and Hughes [42], the FReDs of tenascin-C and tenascin-R share the most sequence similarity at the protein level, which provides one indication that they are derived from the same relatively recent ancestral duplication event. This view is strongly supported by a consideration of the organization of the human genome. The identification of regions of the human genome on different chromosomes that contain many pairs of related genes (paralogs) has provided strong support for the model that the genomes of modern vertebrates evolved from at least one whole genome duplication in early chordate evolution [43]. The largest region of paralogy in the human genome is that between chromosome 1q, which includes the tenascin-R locus at 1q24, and 9q, which contains the tenascin-C locus at 9q33 (block ENSP00000263525 of dataset 5.28; see [43]). The loci of the tenascin-C and tenascin-R genes are also known to be part of the MHC loci that exist as paralogous regions on chromosomes 1q, 6p, 9q and 19p. Thus, although tenascin-X is the most divergent family member in terms of protein sequence, its gene locus on chromosome 6q21.3 is paralogous with that of the tenascin-C and -R genes [44,45]. The paralogy of the MHC region is well-conserved in mice and, according to comparative mapping information, human chromosome 6 corresponds to chicken chromosome 16 [46]. Interestingly, the chicken tenascin-Y gene is located on chromosome 16 and is in immediate synteny with the complement C4, TAP1, TAP2 and MHC II-like genes that represent orthologs of the genes present in the human MHCIII locus next to the human tenascin-X gene (e.g., see [14,15]). As noted above, the gene neighbors of *T. nigroviridis *tenascin-X also correspond to MHC genes. This conserved chromosomal location between chicken tenascin-Y and its neighboring genes and the human, mouse and *T. nigriviridis *tenascin-X genes provides the most convincing argument that, despite distinctions in the polypeptide sequences, these genes all represent tenascin orthologs.

Although a fourth paralogous MHC gene cluster is encoded on human chromosome 19p13-1-13.3, no tenascin is encoded in this region, suggesting that this paralog was lost after the initial large-scale duplication events [44]. The locus of tenascin-W adjacent to tenascin-R on chromosome 1 is suggestive of a distinct evolutionary origin, for example by local tandem duplication of an ancestral tenascin gene. Because the chromosome 1 and 9 MHC regions are so well-related as to indicate their origin from the most recent large-scale duplication [44], the positioning of tenascin-W adjacent to tenascin-R suggests either that: 1) this positioning was ancestral to the duplication event and a tenascin gene has been lost or transposed away from adjacent the tenascin-C gene; or 2) the tenascin-W gene arose by a tandem duplication of tenascin-R after the original duplication event and with a relatively rapid rate of change to the polypeptide sequence. It can also be hypothesized that the tenascin-W gene was transposed from the original locus on chromosome 19 into its current location soon after the duplication that gave rise to the tenascin-C and tenascin-R genes. On present evidence we cannot distinguish between these models, but it is interesting to note that the tenascin-W protein sequence is equivalently related (around 34%-36%) to both tenascin-C and tenascin-R. Finally, in *C. intestinalis*, the single tenascin gene is adjacent to a Notch gene just like all vertebrate tenascins which are located in close proximity to a Notch gene present in the MHC paralogous regions, supporting the common ancestry.

## Discussion

Six different names are currently being used to describe the tenascins of vertebrates: tenascin-C, tenascin-R, tenascin-X, tenascin-Y, tenascin-W and tenascin-N. Analysis of intron-exon splice sites, phylogenetic relationships and synteny shows that this nomenclature is inaccurate and needlessly complicated, and that there are in fact only four vertebrate tenascins. The original designations of tenascin-C and tenascin-R are confirmed by our analyses, but avian tenascin-Y shares close phylogenetic and genomic relationships with tenascin-X, and tenascin-N shares phylogenetic, syntenic and intron-exon junction homologies with tenascin-W. Since the names tenascin-X and tenascin-W have clear precedence in the literature and have been adopted in the majority of published reports, we recommend that use of the terms tenascin-Y [16,47-51] and tenascin-N [19] be discontinued.

Here we report the first example of a tenascin from a non-vertebrate. A predicted tenascin was found in the genomic sequence of the invertebrate chordate *C. intestinalis *and is corroborated as an expressed gene by the identification of matching ESTs, by cDNA sequencing and by immunohistochemistry. A similar gene product was also identified in the related species *C. savignyi*. With only one copy of tenascin, *C. intestinalis *may represent an ideal model system for future studies of tenascin function, since analysis of its knockdown by morpholinos or its misexpression would not be complicated by the possible compensatory action of related tenascin gene products. We have also identified a tenascin-type FReD domain in *O. dioica *and expect to identify a full length tenascin when the genomic sequencing is completed and assembled. Thus, tenascins are most likely common to all urochordates.

There are no tenascins in *Caenorhabiditis elegans *and *Drosophila melanogaster*. It is now recognized that these organisms have undergone extensive gene loss and that the Cnidaria have a higher level of gene conservation in comparison to vertebrates [52]. Nevertheless, we could not identify Cnidarian tenascins from the various databases (see Methods). Searches of the draft genome sequence of the echinoderm *Strongylocentrotus purpuratus *have also not revealed tenascin genes. Thus, to date, tenascins appear to be exclusive to the chordate lineage. Sequences encoding a tenascin-like FReD domain are included in the *Branchiostoma floridae *EST database [GenBank EST:CF919227] [GenBank EST:CF919269] [53], but it is unknown if this sequence includes adjacent FN type III domains and EGF-like repeats. It will be of interest to know if one or more tenascin is encoded in the amphioxus genome once this sequencing project is completed. Of the four vertebrate tenascins, tenascin-X has the most distinctive FReD sequence and overall domain organization. We hypothesize that tenascin-X arose from the first tenascin gene duplication in vertebrates.

Analysis of two pufferfish genomes revealed not four, but five, tenascins. The fifth tenascin appears to be the result of a relatively recent duplication of the tenascin-C gene. In keeping with the established protocol for naming very similar duplicated genes, we propose that these tenascin-C paralogs be referred to as tenascin-Ca and tenascin-Cb. The two pufferfish studied here are closely related, having diverged from a common ancestor only 18–30 million year ago [54]. Nevertheless, the tenascin-Cb genes of these close relatives encode different numbers of EGF-like repeats and FN type III domains. This illustrates the potential for the numbers of these repeats and domains to change during evolution (see also Hughes [42]) and points to the potential problems that can stem from giving different names to tenascins on the basis of the numbers of their repeated domains. It will be interesting to study the expression of these tenascin-C genes in *Tetraodon nigroviridis *and *Takifugu rubripes*: do their expression patterns overlap, or have they evolved distinctive regulatory elements? If so, has their function diverged? There is considerable evidence that the great species diversity of bony fishes is the consequence of an additional duplication of the whole fish genome followed by massive gene loss since the appearance of the ancestral tetrapod (e.g., see [54]). Interestingly, in the family Tetraodontidae the only tenascin to persist as a duplicated gene is tenascin-C. Searches of the latest zebrafish genome assembly (Zv5, Wellcome Trust Sanger Institute) identify only one tenascin-C on chromosome 5 (NP_ 570982). It will be interesting to establish if the persistence of tenascin-C paralogs is specific to the pufferfish lineage.

The tenascin-X gene in pufferfish encodes previously undescribed, highly charged repeats. We propose the name "GK repeats" to describe these unique sequences, since all but one of the 19 repeats in *T. rubripes *and all of the repeats in *Tetraodon nigroviridis *begin with the amino acids glycine and lysine. Given the concentration of tenascin-X in the epimysium of birds and mammals, it is intriguing that these repeats combined with the rest of the DUF619 domain share some sequence homology with a prokaryotic collagen-binding protein and the muscle-specific protein UNC-89 in *C. elegans*. The observation that *X. tropicalis *tenascin-X contains a region with sequence similar both to UNC-89 as well as bird and human tenascin-X leads us to suggest that this region may have a role that is important to tenascin-X function, and its biological properties should be studied further.

A previous study in the zebrafish [17] reported five FN type III domains in the cDNA sequence of tenascin-W. The analysis of the genomic sequence of *D. rerio *as well as RT-PCR of zebrafish cDNA reveals a sixth FN type III domain. Thus, the *D. rerio *tenascin-W gene encodes the same number of FN type III domains as does the orthologous chicken gene, yet it encodes two more than does *Tetraodon nigroviridis*. This diversity again points out the potential hazards of giving proteins a new name based on the number of repeated domains – of the four tenascin subfamilies, only the tenascin-Rs have the same number of FN type III domains encoded in the genes of fish, birds and mammals. In the case of mammalian tenascin-C and tenascin-X, distinct processes of conservative or concerted evolution have been described for their FN type III domains [42]. In the case of tenascin-W most of the diversity can be traced to different numbers of duplications in the third FN type III domain. This domain exists as a single copy in *T. nigroviridis*, two copies in *Takifugu rubripes*, three copies in *D. rerio *and *G. gallus*, 6 copies in man, and 9 copies in many other mammals including dog [NCBI Protein:XP_547455], rat [NCBI Protein:XP_222794] and mouse. What is it about this particular FN type III domain that may be leading to its duplication? One possibility is that this domain contains an integrin binding site, and different numbers of integrin binding sites may change the affinity of the intact molecule for a receptor, or may allow for tenascin-W to crosslink numerous receptors. Note that the LDVP/IDAP/IDSP motif, which has been shown to recognize alpha4beta1 integrin in fibronectin and VCAM [55], is found in almost all of the duplicated third FN type III domains analyzed here (underlined in Figure 6). Tenascin-W from *X. tropicalis *is different from other tenascin-Ws in its number of EGF-like repeats and the relatively recent duplication of its fourth, and not third, FN type III domain. Interestingly, this also results in the duplication of two potentially active integrin binding motifs: an RGD in the fourth domain and a KGD in the fifth. It is intriguing to note that species that lack an RGD motif in the third FN type III domain of tenascin-C (i.e., *X. tropicalis *and mouse) have an RGD motif in their tenascin-W. Since tenascin-C and tenascin-W are often co-expressed during development [17,18,51] it is interesting to speculate that they may have overlapping functions related to their binding to an RGD-dependent integrin.

Most tenascins studied to date are alternatively spliced, and only rarely are all of the potential FN type III domains identified in the final protein (e.g., see [56,57]). This variability leads to different functional domains being exposed in different tissues [1,7,58]. As more ESTs become available, it will be interesting to analyze the many potential splice variants of each of these tenascins, as they may reveal additional functional variability in the tenascin gene family.

## Conclusion

Tenascins are not known in protostomes or Cnidaria. We provide evidence that a single tenascin is encoded in the genome of the urochordate *C. intestinalis*. This invertebrate chordate tenascin contains the motif RGE in an exposed loop of its third FN type III domain, which may correspond to an integrin-binding site conserved in some vertebrate tenascins. Sequence alignments, analysis of domains and exon/intron organization and phylogenetic analyses of tenascins from four classes of vertebrates reveal that in fish and in tetrapods there are four members of the tenascin gene family: tenascin-C, tenascin-R, tenascin-X and tenascin-W. We suggest that use of the names tenascin-Y and tenascin-N be discontinued. In pufferfish there are two tenascin-C paralogs but single copies of the other tenascins. The human genome provides clear evidence that tenascin-C, tenascin-R and tenascin-X arose through the same ancestral genome duplications. Tenascin-W may have evolved as a result of a local duplication of the ancestor of tenascin-C and -R. The tenascin-X genes from the pufferfish *Tetraodon nigroviridis *and *Takifugu rubripes *encode different numbers of unique, highly charged tandem repeats of unknown function. *X. tropicalis *tenascin-X shares features with both the smaller teleost tenascin-Xs and the very large tenascin-Xs found in mammals. Finally, much of the diversity seen in the size of tenascin-W can be accounted for by the multiple duplications of the exon encoding the third FN type III domain in different species.

## Methods

### Sequences and bioinformatics

The NCBI Protein Database accession numbers of complete or partial tenascin amino acid sequences identified by key-word search or BLASTP (NCBI Basic Local Alignment Search Tool [59]) (reviewed by [60]) and used to obtain data for searching, alignment and phylogenetic reconstructions are listed in Table 1. Genomes were searched and analyzed at the NCBI [39], UCSC Genome Bioinformatics [24], Joint Genomic Institute Eukaryotic Genomics [61], the Laboratory for Developmental Biology and Genome Biology [26], Centre National de Séquençage Genoscope [62], the Max Planck Institute for Molecular Genetics [63], the Cnidarian Evolutionary Biology Database [64] and the Nematostella vectensis Genomics Database [65]. Sequence alignment and phylogenetic trees were constructed simultaneously by using SATCHMO (Berkeley Phylogenomics [66]) as described by Edgar and Sjölander [38]. The phylogenetic tree of the FReDs was constructed with MrBayes: Bayesian Inference of Phylogeny [67] (see [68]) using the WAG substitution model [69]. We ran 1 million generations after which the average standard deviation of split frequencies was 0.004518. The phylogenetic tree was drawn using DrawTree [70]. For identification of parologous tenascin-encoding regions in the human genome, the database of "Paralogons in the human genome", version 5.28, was searched (Paralogons in the Human Genome [71]) (see [43]).

### Domain and repeat identification

Likely heptad repeats were identified using the Simple Modular Architecture Research Tool SMART [72], Paircoil Scoring Form [73] (see also [74]) and COILS [75] programs. Tenascin-type EGF-like repeats were identified by their characteristic number and spacing of cysteine residues (X_4_CX_3_CX_5_CX_4_CXCX_8_C) and confirmed with the SMART program, which classifies them as generic EGF repeats. FReDs and FN type III domains were identified by the NCBI Conserved Domain feature of BLASTP [59] and by SMART [72]. Partial FN type III domains were also identified by the NCBI Conserved Domain program. Often the different programs for predicting open reading frames in pufferfish and *X. tropicalis *were not in agreement. For example, the sequence ELDAPSDLSAQDVTESSFTVSRDSTQVHIDGYFLSFSSSAGSN was predicted to lie between the third and fourth FN type III domains of *Tetraodon nigroviridis *tenascin-W at the NCBI protein database [CAG07652], but not in predicted protein at the Genoscope site (GSTENT00028391001). In such cases the proteins were aligned with avian and mammalian tenascin sequences using the SATCHMO program and the predicted protein with the best fit to known cDNA sequences was used. Additional short predicted open-reading frames were not considered to be part of the protein if the BLASTP and SMART programs predicted them to be potentially artifactual due to low-complexity. Intron-exon splice sites were estimated from the results of the "build protein" feature of BLASTP genomic searches and from the gene model feature of the *Tetraodon *genome browser [62].

### Molecular cloning, antibody production and immunohistochemistry

The sequence of the N-terminus of *Ciona intestinalis *tenascin was predicted from genomic sequence 5' of the EGF-like repeats encoding a potential signal peptide. This was confirmed by the identification of a cDNA using RT-PCR with primers homologous to the region harboring the methionine N-terminal to the predicted signal peptide and a primer from the next exon. Animals were obtained from the Marine Biological Laboratory (Woods Hole, MA). RNA was extracted from larvae collected 18 hours after fertilization and used as template for reverse transcription. PCR was performed with the primer pair 5'-ATGTGGCCTGTTTCGAGTCG-3'/5'-ATTGCTGCTGGTCAGGAACG-3' and the resulting band sequenced. It encoded amino acids 1–66 shown in Figure 2A. To raise antibodies we amplified the cDNA encoding the first FN type III domain from *C. intestinalis *mRNA by RT-PCR with the primers 5'-TCTCATGTCATCAAACCATCAG-3'/5'-TGTTTTCACAGAAGCAGTAATTGG-3'. The resulting cDNA encoded amino acids 459–550 from the sequence shown in Figure 2A. Interestingly, the DNA sequence was only 86.8% identical to the exons of the genomic sequence (Ciona intestinalis Genome [77]), but all of them were silent mutations resulting in a 100% conserved protein sequence. These differences are possibly due to the genomic diversity that has been noted by others between specimens of *C. intestinalis *from the Pacific and Atlantic [78]. This protein fragment was expressed in *E. coli *and antiserum against the purified protein was raised in rabbits. The antiserum recognized a large (>250 kDa) smeared band on immunoblots of homogenates of recently hatched *C. intestinalis *larvae (generously provided by W.R. Jeffery, University of Maryland) as well as smaller bands that were also present on the blot incubated with the preimmune serum. For immunostaining of whole mounts, larvae were fixed in 4% paraformaldehyde in sea water and permeabilized in methanol at -20C. Larvae were then rinsed in phosphate buffered saline with 0.01% Tween-20 (PBT), blocked in 0.1% bovine serum albumin in PBT, and incubated overnight in the anti-*Ciona *tenascin serum (1:100) or similarly diluted preimmune serum. Larvae were then rinsed overnight and incubated in goat anti-rabbit Alexa 594 secondary antibody (1:500) in PBT overnight. After extensive washes the immunostained larvae were coverslipped and observed using an Olympus confocal microscope.

### Reverse Transcriptase Polymerase Chain Reaction

The cDNA encoding the fifth FN type III domain of *D. rerio *tenascin-W was cloned in the following way: total RNA from an adult fish was isolated with Trizol reagent (Invitrogen) and mRNA was isolated with the RNeasy mRNA purification kit (Qiagen). First strand cDNA was generated with SuperScript III reverse transcriptase (Invitrogen) according to the manufacturer's recommendation. RT-PCR (30 cycles 94°C 1 min, 94°C 50 sec, 60°C 1 min, 72°C 2 min) using primers corresponding to the sequences from neighboring exons (5'-GAGTTCAACAGAAGCGGAAAC-3'/5'-TTGAGTCTGAACATCAGTGGC-3') generated an appropriately sizedproduct that was sequenced. The cloned cDNA corresponded to the FN type III domain found in the genomic sequence encoded on asingle exon between the fourth and sixth FN type III domains of tenascin-W (99% identical, as opposed to 81% identical to the fourth FN type III domain). The cDNA coded protein that was identical to the protein predicted from the genomic sequence except that athreonine replaced an isoleucine (the 53rd residue of the domain). This sequence was confirmed by the use of asecond independent pair of primers (5'-AAGCGGAAACAGATATAGACGC-3'/5'-AATCTCTGCTGTTTCAGCCTC-3').

## Authors' contributions

RPT identified and analyzed the tenascins from fish and *Xenopus*, performed the whole mount immunohistochemistry, wrote the first draft of the paper and prepared Figures 1, 2, 3, 4, 5, 6, 7, 8. KD with the help of JF cloned and sequenced a fragment of the *Ciona *tenascin, raised the antiserum against the recombinant *Ciona *tenascin fragment, developed the phylogenetic tree (Figure 9), carried out the *D. rerio *RT-PCR with JF, and contributed significantly to the text. JFH provided assistance with the various genome and domain identification databases, clarified the use of terminology and assisted with the content and organization of the manuscript. JF conducted the *D. rerio *RT-PCR and *Ciona *tenascin cloning with KD. RC-E identified and analyzed the *Ciona *tenascin sequence, analyzed tenascin-X synteny and contributed to major revisions to the manuscript. JCA and RPT conceived the project. JCA provided advice on web-based bioinformatics resources, identified *Ciona *tenascin and the putative amphioxus tenascin through ESTs, and contributed extensively to the studies of tenascin synteny and each draft of the text. All authors read and approved the final manuscript.

## Supplementary Material

Additional file 1 Side-by-side alignment of predicted tenascins from Tetraodon. Click here for file

Additional file 2 Side-by-side alignment of predicted tenascins from Takifugu. Click here for file

Additional file 3 Side-by-side alignment of predicted tenascins from Xenopus. Click here for file
