# Supplementary material for: Phylogenetic analysis of the tenascin gene family: evidence of origin early in the chordate lineage
Source: BMC Evol Biol. 2006 Aug 7;6:60. doi: 10.1186/1471-2148-6-60 (PMC1578592; doi:10.1186/1471-2148-6-60)
Supplement: Additional file 1 — Side-by-side alignment of predicted tenascins from Tetraodon. [file 1471-2148-6-60-S1.doc]

Appendix 1: Side-by-side alignment of predicted tenascins from Tetraodon.

Tetraodon tenascin-CA:

00000001 M G T K T I L L V C L S V S L L F Q L S 00000060

>>>>>>>> | | | | | | | | | | | | | | | | | | | | <<<<<<<<

20082225 atgggcacaaaaacgatcctcttggtttgtctgtccgtgagcctcctcttccagctctcc 20082284

00000061 A A G L V R K I I R H R R E T L M P K K 00000120

>>>>>>>> | | | | | | | | | | | | | | | | | | | | <<<<<<<<

20082285 gctgcaggcctggtcagaaagataatccgtcaccgcagagaaactctgatgcccaaaaag 20082344

00000121 S Q E N L T L P H P D Q P V V F N H V Y 00000180

>>>>>>>> | | | | | | | | | | | | | | | | | | | | <<<<<<<<

20082345 tcccaggagaacctcacgctgccccatccagaccagccggtggtctttaaccacgtctat 20082404

00000181 N I N V P S T S L C S V D L D L P G E P 00000240

>>>>>>>> | | | | | | | | | | | | | | | | | | | | <<<<<<<<

20082405 aacatcaacgtcccctccacctccctctgctccgtggaccttgatttgccaggagagccg 20082464

00000241 E V K H K T P L R G V Q N M E H I E H T 00000300

>>>>>>>> | | | | | | | | | | | | | | | | | | | | <<<<<<<<

20082465 gaggtcaaacacaagacaccgctgagaggagtgcagaacatggagcacatagagcacact 20082524

00000301 E D G D N Q I V F T H R I T I P K Q A C 00000360

>>>>>>>> | | | | | | | | | | | | | | | | | | | | <<<<<<<<

20082525 gaagacggcgacaaccagattgtgtttacacaccgcatcactatcccgaagcaggcgtgc 20082584

00000361 S C E N Q L L D V K S I L N R L E M L E 00000420

>>>>>>>> | | | | | | | | | | | | | | | | | | | | <<<<<<<<

20082585 agctgtgagaaccagctgctggacgttaaatccatcctgaataggctggagatgctggag 20082644

00000421 L E L S S L R E Q C S G G A G C C G A Q 00000480

>>>>>>>> | | | | | | | | | | | | | | | | | | | | <<<<<<<<

20082645 ttggagttgtcgagtctgagggagcagtgcagcggtggggctggctgctgcggagctcag 20082704

00000481 V A 00000486

>>>>>>>> | | <<<<<<<<

20082705 gttgca 20082710

00000487 G E I S T K P Y C N G R G N W S S D T C 00000546

>>>>>>>> | | | | | | | | | | | | | | | | | | | | <<<<<<<<

20083329 ggtgaaatttccaccaaaccctactgtaacggccgtggcaactggagcagcgacacgtgc 20083388

00000547 S C L C E V G W K G Q N C S E P D C P G 00000606

>>>>>>>> | | | | | | | | | | | | | | | | | | | | <<<<<<<<

20083389 agctgcctctgcgaggtgggatggaaaggccagaactgcagcgagcccgactgtcccggc 20083448

00000607 D C Q D Q G R C L N G R C E C F E G F G 00000666

>>>>>>>> | | | | | | | | | | | | | | | | | | | | <<<<<<<<

20083449 gactgccaggaccagggccgctgcctgaacggcagatgcgaatgcttcgagggtttcggc 20083508

00000667 G D D C S A E L C L L D C G D Y G H C V 00000726

>>>>>>>> | | | | | | | | | | | | | | | | | | | | <<<<<<<<

20083509 ggagacgactgcagcgccgagctctgcctgctggactgcggcgactacggccactgcgtc 20083568

00000727 S G V C L C E E G F S G Q D C S Q T N C 00000786

>>>>>>>> | | | | | | | | | | | | | | | | | | | | <<<<<<<<

20083569 agcggcgtgtgcctgtgtgaggagggcttcagcgggcaggactgcagccagaccaactgc 20083628

00000787 L N S C L G R G R C L E D E C V C D E P 00000846

>>>>>>>> | | | | | | | | | | | | | | | | | | | | <<<<<<<<

20083629 ctcaacagctgcctgggccgcgggcgctgcctggaggacgagtgcgtctgcgatgagccc 20083688

00000847 W T G L D C S E L I C P N D C Y D R G R 00000906

>>>>>>>> | | | | | | | | | | | | | | | | | | | | <<<<<<<<

20083689 tggactggactggactgctctgaactcatctgtccaaacgactgctacgaccgcggacgc 20083748

00000907 C L N G T C E C E Q G Y A G E D C G D L 00000966

>>>>>>>> | | | | | | | | | | | | | | | | | | | | <<<<<<<<

20083749 tgccttaacggcacctgtgagtgcgagcagggctacgccggggaggactgtggggattta 20083808

00000967 S C P G L C S N R G V C L N G Q C V C Q 00001026

>>>>>>>> | | | | | | | | | | | | | | | | | | | | <<<<<<<<

20083809 tcctgtcctggtctttgtagcaaccgcggcgtgtgcctaaatggccagtgtgtgtgccag 20083868

00001027 S G Y S G D D C S K L T C P K N C N E K 00001086

>>>>>>>> | | | | | | | | | | | | | | | | | | | | <<<<<<<<

20083869 agcggctacagcggagacgactgctccaagctgacctgtccaaagaactgcaacgagaaa 20083928

00001087 G H C F N G K C I C D P G R E G E D C S 00001146

>>>>>>>> | | | | | | | | | | | | | | | | | | | | <<<<<<<<

20083929 ggccattgcttcaacgggaagtgtatctgcgatccgggacgcgaaggggaggattgctcc 20083988

00001147 V L S C P D N C N D R G Q C V D G A C V 00001206

>>>>>>>> | | | | | | | | | | | | | | | | | | | | <<<<<<<<

20083989 gtcctctcttgccctgacaactgcaacgaccggggtcagtgcgtggatggagcatgcgtg 20084048

00001207 C D A G Y Q G E D C G A L S C P N N C L 00001266

>>>>>>>> | | | | | | | | | | | | | | | | | | | | <<<<<<<<

20084049 tgtgatgctggctatcagggcgaggactgcggtgctctttcctgtcccaacaactgtctg 20084108

00001267 D R G N C V N G Q C V C D K G Y S G E D 00001326

>>>>>>>> | | | | | | | | | | | | | | | | | | | | <<<<<<<<

20084109 gaccgtggaaactgtgttaacggacagtgcgtctgtgacaaaggctacagtggggaggac 20084168

00001327 C S V K T C P K K C M E R G D C V D G K 00001386

>>>>>>>> | | | | | | | | | | | | | | | | | | | | <<<<<<<<

20084169 tgcagtgtcaagacctgtccaaagaagtgcatggaacgtggtgattgcgtggacggcaag 20084228

00001387 C M C F P G F K G K D C G E M T C P G D 00001446

>>>>>>>> | | | | | | | | | | | | | | | | | | | | <<<<<<<<

20084229 tgcatgtgctttcccggttttaagggcaaggactgcggtgagatgacctgtcctggagac 20084288

00001447 C S N Q G R C E S G K C V C H K G Y T G 00001506

>>>>>>>> | | | | | | | | | | | | | | | | | | | | <<<<<<<<

20084289 tgcagcaaccagggccgctgtgaaagtggaaaatgtgtttgccacaaaggttacacaggc 20084348

00001507 E D C S L K T C P K N C H D R G Y C I D 00001566

>>>>>>>> | | | | | | | | | | | | | | | | | | | | <<<<<<<<

20084349 gaagactgcagtctgaagacgtgtcccaaaaactgtcatgacagaggctactgcattgat 20084408

00001567 G N C V C Y E G F T G P D C S T L A C P 00001626

>>>>>>>> | | | | | | | | | | | | | | | | | | | | <<<<<<<<

20084409 ggcaactgtgtgtgttatgaaggcttcactgggccagactgctccaccctggcttgtccc 20084468

00001627 S D C Q N Q G H C K N G V C V C E E G F 00001686

>>>>>>>> | | | | | | | | | | | | | | | | | | | | <<<<<<<<

20084469 agtgactgccagaaccagggacactgcaaaaatggagtatgtgtctgtgaagagggcttc 20084528

00001687 I G E D C S Q 00001707

>>>>>>>> | | | | | | | <<<<<<<<

20084529 attggagaggactgctcacag 20084549

00001708 V S P P K D L T V V E V T P E T V D L S 00001767

>>>>>>>> | | | | | | | | | | | | | | | | | | | | <<<<<<<<

20085295 gtatcgccgcccaaggacctcacggtcgtggaggtgacaccagagacagtggacctgtcc 20085354

00001768 W D N E M R V S E Y L V K Y V P T A P G 00001827

>>>>>>>> | | | | | | | | | | | | | | | | | | | | <<<<<<<<

20085355 tgggacaatgagatgcgtgtttcagagtacctggtcaagtatgtgcccactgctcctgga 20085414

00001828 G L E L D M Q V P G D Q K K A T I L E L 00001887

>>>>>>>> | | | | | | | | | | | | | | | | | | | | <<<<<<<<

20085415 ggcctggagctggacatgcaggtccctggggaccagaagaaggccaccattctagagctt 20085474

00001888 E P G V E Y L I R V Y A V L N N K K S I 00001947

>>>>>>>> | | | | | | | | | | | | | | | | | | | | <<<<<<<<

20085475 gaacctggagtggagtacctgatcagggtctacgctgtgctcaacaacaagaagagcatt 20085534

00001948 P V D A R V A L 00001971

>>>>>>>> | | | | | | | | <<<<<<<<

20085535 cctgtggacgctagagtagctctg 20085558

00001972 D L P K P D G L K F K S V R D T S V E V 00002031

>>>>>>>> | | | | | | | | | | | | | | | | | | | | <<<<<<<<

20085636 gatttaccaaagcctgatgggctcaaattcaagtccgtgcgggacacctcggtagaggtc 20085695

00002032 E W D P L D I P F D G W N L I F R N T 00002088

>>>>>>>> | | | | | | | | | | | | | | | | | | | <<<<<<<<

20085696 gagtgggaccctttggacattccctttgatggctggaacctcatctttagaaacacg 20085752

00002089 K E E D G E I L N S L G Q P E T T F E Q 00002148

>>>>>>>> | | | | | | | | | | | | | | | | | | | | <<<<<<<<

20085981 aaagaagaggatggcgagattctgaactcgcttggccagcccgagaccacctttgagcag 20086040

00002149 S G L G P G Q E Y E V K L E V V K N N K 00002208

>>>>>>>> | | | | | | | | | | | | | | | | | | | | <<<<<<<<

20086041 tcgggcctgggccctggccaggagtacgaggtcaagctggaggtggtgaagaacaacaag 20086100

00002209 R G P P A S K S V I T 00002241

>>>>>>>> | | | | | | | | | | | <<<<<<<<

20086101 cgtggaccacctgcctccaagagcgtcatcaca 20086133

00002245 I D S P N Q V D I R D V T D T T A L L T 00002304

>>>>>>>> | | | | | | | | | | | | | | | | | | | | <<<<<<<<

20086920 atcgattccccaaaccaggtagacatccgagatgtgacggacaccacagcgctgctcacc 20086979

00002305 W L P P V A E V E E V S I S Y G P S S N 00002364

>>>>>>>> | | | | | | | | | | | | | | | | | | | | <<<<<<<<

20086980 tggttgccgcccgtggcagaggtggaggaggttagcatttcctacggacccagctccaac 20087039

00002365 P A D R N V V E L P S T E S Q Y H L G G 00002424

>>>>>>>> | | | | | | | | | | | | | | | | | | | | <<<<<<<<

20087040 cctgcagaccgcaacgtggtggagctgccatctacggaaagccagtaccacctggggggc 20087099

00002425 L D P D T H Y Q V S L T A H K G D W S S 00002484

>>>>>>>> | | | | | | | | | | | | | | | | | | | | <<<<<<<<

20087100 ctcgaccctgacacccactaccaagtttctctgacagcccacaagggggactggagcagc 20087159

00002485 K P V H E S F L T G 00002514

>>>>>>>> | | | | | | | | | | <<<<<<<<

20087160 aaacccgtccacgagtctttcctgacaggt 20087189

00002515 L D A P K N L K T A E I T D E S I T L E 00002574

>>>>>>>> | | | | | | | | | | | | | | | | | | | | <<<<<<<<

20087262 ctggatgctccgaaaaacctgaagacggctgaaataacagacgagagcatcactctggag 20087321

00002575 W E N S R A Q V D N Y R I K Y G P L S G 00002634

>>>>>>>> | | | | | | | | | | | | | | | | | | | | <<<<<<<<

20087322 tgggagaacagtcgcgctcaggttgacaactatcgcatcaagtatggacccctgtctgga 20087381

00002635 G E H R E L L F T P G A K D H T H A K I 00002694

>>>>>>>> | | | | | | | | | | | | | | | | | | | | <<<<<<<<

20087382 ggcgagcacagggagctactcttcacccctggagccaaagaccacactcacgccaaaatc 20087441

00002695 T 00002697

>>>>>>>> | <<<<<<<<

20087442 act 20087444

00002698 G L R A S T E Y G M G V T A V K D E R E 00002757

>>>>>>>> | | | | | | | | | | | | | | | | | | | | <<<<<<<<

20088318 ggcctgagagccagcacagagtatgggatgggtgtaacagcggtgaaagatgagcgggag 20088377

00002758 S L P T T T N A V T 00002787

>>>>>>>> | | | | | | | | | | <<<<<<<<

20088378 agtttgccaacaaccaccaacgcagtgacc 20088407

00002788 A L D S P K D L T V T K V T E T T M L L 00002847

>>>>>>>> | | | | | | | | | | | | | | | | | | | | <<<<<<<<

20089102 gcactggattctccaaaggacctaaccgttaccaaggtgacagagaccaccatgttgctg 20089161

00002848 E W R H P Q A K L D S Y R L V Y V S A D 00002907

>>>>>>>> | | | | | | | | | | | | | | | | | | | | <<<<<<<<

20089162 gagtggagacacccgcaggctaaactggactcttacaggctggtttacgtatctgctgat 20089221

00002908 G H R R E E V L P G D L K S Y S L V D L 00002967

>>>>>>>> | | | | | | | | | | | | | | | | | | | | <<<<<<<<

20089222 ggccacagaagggaggaagtgctgccgggtgatctaaagtcctacagcctggtggacctt 20089281

00002968 S P G T L Y T I S I S A E R G S R T S A 00003027

>>>>>>>> | | | | | | | | | | | | | | | | | | | | <<<<<<<<

20089282 agccccggcacgctttacaccataagcatcagtgcagagcgcggcagcaggaccagcgcc 20089341

00003028 P N T V S A F T 00003051

>>>>>>>> | | | | | | | | <<<<<<<<

20089342 cccaacaccgtctcagcattcaca 20089365

00003052 E E E K P L V T S F T I S D V S W D S L 00003111

>>>>>>>> | | | | | | | | | | | | | | | | | | | | <<<<<<<<

20090959 gaggaggagaagccgctggtgaccagtttcaccatcagcgacgtgtcctgggacagcctc 20091018

00003112 Q L S W E A P D G A F Q A F L V K V T D 00003171

>>>>>>>> | | | | | | | | | | | | | | | | | | | | <<<<<<<<

20091019 cagttgtcttgggaggccccggacggggctttccaggccttcctggtcaaggtgacagat 20091078

00003172 A E T G S E V Q N H S V V A G A R S F A 00003231

>>>>>>>> | | | | | | | | | | | | | | | | | | | | <<<<<<<<

20091079 gcagagactggttccgaggtgcagaaccactcggtggttgctggcgctcggagcttcgcc 20091138

00003232 I S D L S A T T W Y R V S L Y G L H R G 00003291

>>>>>>>> | | | | | | | | | | | | | | | | | | | | <<<<<<<<

20091139 atctctgacctctctgctaccacctggtacagagtcagcctgtacgggctccacaggggg 20091198

00003292 A L L G P V T A D T I T 00003327

>>>>>>>> | | | | | | | | | | | | <<<<<<<<

20091199 gcgctcctagggccagtgacggctgacaccatcaca 20091234

00003328 E A E P E I Q A L L V S A V T P G S F R 00003387

>>>>>>>> | | | | | | | | | | | | | | | | | | | | <<<<<<<<

20092620 gaggctgagccagagatccaggccctcctggtctcggcagtcacccctgggagctttcgg 20092679

00003388 L T W T A E E D A L D T F V I M V S P A 00003447

>>>>>>>> | | | | | | | | | | | | | | | | | | | | <<<<<<<<

20092680 ctgacgtggacggctgaggaggacgctttggacacctttgtgatcatggtcagcccggcg 20092739

00003448 D D P G H P T E L V L A S E Q R S V A V 00003507

>>>>>>>> | | | | | | | | | | | | | | | | | | | | <<<<<<<<

20092740 gatgacccgggtcatcccacagagctggtgctggccagcgagcagcgaagcgtagccgtt 20092799

00003508 T N L T E D T Q Y K I Q M F G L R F G R 00003567

>>>>>>>> | | | | | | | | | | | | | | | | | | | | <<<<<<<<

20092800 acaaacctgacagaggacacgcagtacaaaatccaaatgtttggcctccgttttggaaga 20092859

00003568 R T K S V Q E S V R T 00003600

>>>>>>>> | | | | | | | | | | | <<<<<<<<

20092860 agaaccaaatctgtgcaggaaagtgtcagaaca 20092892

00003601 D L A P P K G I R F S D V T D T S T T V 00003660

>>>>>>>> | | | | | | | | | | | | | | | | | | | | <<<<<<<<

20094301 gatctggcccctccgaaagggatccgcttctctgacgtgactgacacgtccaccaccgtt 20094360

00003661 H W G A P R V R V D S Y Q I T Y V P A H 00003720

>>>>>>>> | | | | | | | | | | | | | | | | | | | | <<<<<<<<

20094361 cactggggggctccaagagtgcgagtggacagctatcagatcacctacgtccctgcccat 20094420

00003721 G 00003723

>>>>>>>> | <<<<<<<<

20094421 gga 20094423

00003724 G S A K T L T V D G S K S Q T V L P N L 00003783

>>>>>>>> | | | | | | | | | | | | | | | | | | | | <<<<<<<<

20094830 ggaagtgcaaagacactgacagtggacggttccaagtctcagactgtgctgcccaacctg 20094889

00003784 T P G V T Y E V T I V A V K G S R E S L 00003843

>>>>>>>> | | | | | | | | | | | | | | | | | | | | <<<<<<<<

20094890 acccctggggtcacctatgaggtcaccatcgttgccgtcaaagggtcaagagagagtctg 20094949

00003844 P A S D S I T T G 00003870

>>>>>>>> | | | | | | | | | <<<<<<<<

20094950 ccagcgtccgacagcatcaccacaggc 20094976

00003871 A L D K P R G L V A V N I T D T G A L L 00003930

>>>>>>>> | | | | | | | | | | | | | | | | | | | | <<<<<<<<

20095115 gctctggataaacctcgtgggctggttgccgtcaacatcaccgacactggggctctgttg 20095174

00003931 L W Q P A I A T I D G Y V I T Y S A D A 00003990

>>>>>>>> | | | | | | | | | | | | | | | | | | | | <<<<<<<<

20095175 ctctggcaaccagccatcgctacgatagacggttacgtcatcacgtacagcgcagacgca 20095234

00003991 G I S S I 00004005

>>>>>>>> | | | | | <<<<<<<<

20095235 ggtatttcctccatc 20095249

00004006 M E R V S G N V M E F Q M S S L V P A T 00004065

>>>>>>>> | | | | | | | | | | | | | | | | | | | | <<<<<<<<

20095550 atggagcgagtttctgggaacgtgatggagttccagatgtcctctctggttcctgccacc 20095609

00004066 R Y T V K V F A A R D L A K S A A T S T 00004125

>>>>>>>> | | | | | | | | | | | | | | | | | | | | <<<<<<<<

20095610 cggtacacagtcaaggtgtttgcagccagagacttggccaagagcgcagccaccagcacg 20095669

00004126 E F T T 00004137

>>>>>>>> | | | | <<<<<<<<

20095670 gagttcacaacc 20095681

00004138 D V D T P T H L A A S S I Q T E S A M L 00004197

>>>>>>>> | | | | | | | | | | | | | | | | | | | | <<<<<<<<

20095797 gacgtggacactcccactcatttagcagccagtagcatccaaacggagagcgccatgcta 20095856

00004198 T W K A P R A S I T G Y I L S F E A V D 00004257

>>>>>>>> | | | | | | | | | | | | | | | | | | | | <<<<<<<<

20095857 acctggaaggccccacgcgctagcatcaccggctacatcctcagcttcgaggcggttgat 20095916

00004258 G T V R 00004269

>>>>>>>> | | | | <<<<<<<<

20095917 ggcacagtccgc 20095928

00004270 E V V L S P T A V S Y N M A Q L S A S T 00004329

>>>>>>>> | | | | | | | | | | | | | | | | | | | | <<<<<<<<

20095998 gaagtggtcctgagccccaccgctgtgtcctacaacatggctcagctaagcgcttccaca 20096057

00004330 E Y S V R L Q A I A G P K R S R V V A A 00004389

>>>>>>>> | | | | | | | | | | | | | | | | | | | | <<<<<<<<

20096058 gagtactcggtcaggctgcaggccatcgctggtcccaagaggagtagggtggttgcagcc 20096117

00004390 V F T T 00004401

>>>>>>>> | | | | <<<<<<<<

20096118 gtcttcaccacc 20096129

00004405 G V Q Y R H P R D C S Q V L L N G D G A 00004464

>>>>>>>> | | | | | | | | | | | | | | | | | | | | <<<<<<<<

20096474 ggtgtgcagtaccgacaccccagagactgctcccaggttctgctgaacggtgacggtgct 20096533

00004465 S G P Y T I F L G G D E N Q P L Q V Y C 00004524

>>>>>>>> | | | | | | | | | | | | | | | | | | | | <<<<<<<<

20096534 tcgggcccgtacaccatcttcctgggtggggatgagaaccagccgctgcaggtctactgt 20096593

00004525 D M S T D G G G W I 00004554

>>>>>>>> | | | | | | | | | | <<<<<<<<

20096594 gacatgagcaccgacggaggcggatggatc 20096623

00004555 V F L R R Q S G K L D F F R N W K N Y T 00004614

>>>>>>>> | | | | | | | | | | | | | | | | | | | | <<<<<<<<

20097091 gttttcctcagacgccaaagcgggaagctggattttttccgcaactggaagaattacacg 20097150

00004615 S G F G D M N D E F W L 00004650

>>>>>>>> | | | | | | | | | | | | <<<<<<<<

20097151 tccggctttggagatatgaacgacgaattctggctg 20097186

00004651 G L S N L N K I T A A A Q Y E L R V D L 00004710

>>>>>>>> | | | | | | | | | | | | | | | | | | | | <<<<<<<<

20097307 ggtctgtccaacctgaataagatcacagccgcggctcagtacgagctcagggtggacctg 20097366

00004711 R D K G E T A F A Q Y D R F S V S E P R 00004770

>>>>>>>> | | | | | | | | | | | | | | | | | | | | <<<<<<<<

20097367 agggacaaaggcgagacggccttcgcccagtacgaccgcttctccgtctccgagccgcgg 20097426

00004771 S R Y K V H V G G Y S G T 00004809

>>>>>>>> | | | | | | | | | | | | | <<<<<<<<

20097427 agccgctacaaagtccacgttggaggttacagcgggaca 20097465

00004810 A G D S M T Y H H G R P F S T Y D H D N 00004869

>>>>>>>> | | | | | | | | | | | | | | | | | | | | <<<<<<<<

20098136 gcaggtgactccatgacctaccaccacggacgtcccttttccacctacgaccatgacaac 20098195

00004870 D I A V T N C A L S Y K G A F W Y K N C 00004929

>>>>>>>> | | | | | | | | | | | | | | | | | | | | <<<<<<<<

20098196 gacatcgccgtcaccaactgtgctctgtcctacaagggagccttctggtacaaaaactgc 20098255

00004930 H R V N L M G R Y G D N S H S K 00004977

>>>>>>>> | | | | | | | | | | | | | | | | <<<<<<<<

20098256 catcgcgtcaacctcatggggcgatatggagacaacagtcacagcaag 20098303

00004978 G V N W F H W K G H E H S I E F A E M K 00005037

>>>>>>>> | | | | | | | | | | | | | | | | | | | | <<<<<<<<

20098518 ggcgtaaactggttccactggaaaggtcatgagcattccattgagtttgctgagatgaag 20098577

00005038 L R P S N F R N L E G R R K R S 00005085

>>>>>>>> | | | | | | | | | | | | | | | | <<<<<<<<

20098578 ctgaggccgtccaactttagaaacctggagggaagacggaaacgatcc 20098625

Tetraodon tenascin-CB:

0000001 M A A K G L L G C L L L L A L L S V S K 0000060

>>>>>>> | | | | | | | | | | | | | | | | | | | | <<<<<<<

1826791 atggctgccaaaggcctcctgggatgcctcctcctgctggctttgctcagcgtatcaaag 1826850

0000061 A G L V G K I L R H R R Q T L A A P E E 0000120

>>>>>>> | | | | | | | | | | | | | | | | | | | | <<<<<<<

1826851 gccggcctggtggggaaaatcctgcgccatcgccgccagactttggccgcccctgaagaa 1826910

0000121 F N T S V P S A G Q P V V F N H V Y N I 0000180

>>>>>>> | | | | | | | | | | | | | | | | | | | | <<<<<<<

1826911 tttaacaccagtgtgcccagcgcgggccagcctgtggtcttcaaccacgtctacaacatc 1826970

0000181 N V P A S S L C S V S L D S P D S A L L 0000240

>>>>>>> | | | | | | | | | | | | | | | | | | | | <<<<<<<

1826971 aacgttcccgctagttccttgtgctcggtcagcctggactcgcccgacagcgcgctgctg 1827030

0000241 E A Q E G T G H Q S T E H T V D G D N Q 0000300

>>>>>>> | | | | | | | | | | | | | | | | | | | | <<<<<<<

1827031 gaagcccaggaaggaacaggccatcaaagcaccgagcacaccgtggacggggacaaccag 1827090

0000301 I I F T H R I N I P R Q A C G C S D D L 0000360

>>>>>>> | | | | | | | | | | | | | | | | | | | | <<<<<<<

1827091 atcatcttcacccatcgtatcaacatacctcggcaggcctgcggctgctccgacgacctg 1827150

0000361 L G L R D L L S R L E M L E G E V S A L 0000420

>>>>>>> | | | | | | | | | | | | | | | | | | | | <<<<<<<

1827151 ctcggcctcagggacctcctgagtcgcctggagatgctcgaaggagaggtttcagccctg 1827210

0000421 K D Q C N G E R M C C G S Q V T 0000468

>>>>>>> | | | | | | | | | | | | | | | | <<<<<<<

1827211 aaagatcagtgtaacggcgagagaatgtgctgcggatcgcaagtcaca 1827258

0000469 G E V A F T P Y C N G H G N Y S S E T C 0000528

>>>>>>> | | | | | | | | | | | | | | | | | | | | <<<<<<<

1827326 ggcgaggtggccttcacaccctactgtaacggccacggcaactacagcagcgagacctgc 1827385

0000529 S C T C E P G W R G A N C T E L D C P G 0000588

>>>>>>> | | | | | | | | | | | | | | | | | | | | <<<<<<<

1827386 agctgcacgtgtgagcccggctggaggggagccaactgcaccgagctggactgccccggg 1827445

0000589 D C Q G R G R C V N G T C Q C L E G F G 0000648

>>>>>>> | | | | | | | | | | | | | | | | | | | | <<<<<<<

1827446 gactgccagggtcggggccgctgcgttaacggaacgtgccaatgcctcgaaggcttcggg 1827505

0000649 G E N C T V E E C P V G C G T N G R C V 0000708

>>>>>>> | | | | | | | | | | | | | | | | | | | | <<<<<<<

1827506 ggggaaaactgcacggtcgaggaatgtcctgtgggatgcggcaccaacggcaggtgcgtc 1827565

0000709 A G T C V C S S G F F G G D C S Q T E C 0000768

>>>>>>> | | | | | | | | | | | | | | | | | | | | <<<<<<<

1827566 gctgggacctgcgtctgttccagtggcttcttcggcggggactgctcccagaccgagtgc 1827625

0000769 L N N C W R R G R C E G Q V C V C D Q P 0000828

>>>>>>> | | | | | | | | | | | | | | | | | | | | <<<<<<<

1827626 ctgaacaactgctggcgccgtggccgctgcgagggccaggtctgcgtgtgcgatcagccc 1827685

0000829 W T G A D C S E L L C P K D C L S R G R 0000888

>>>>>>> | | | | | | | | | | | | | | | | | | | | <<<<<<<

1827686 tggaccggcgccgactgctcggagctcctctgccccaaagactgcctctcccgtggacgc 1827745

0000889 C E N G T C Y C D E G Y A G E D C G Q R 0000948

>>>>>>> | | | | | | | | | | | | | | | | | | | | <<<<<<<

1827746 tgcgagaacggcacctgctactgcgatgaggggtacgccggggaggattgcggccagcgc 1827805

0000949 T C P G K C H G N G F C V D G R C V C I 0001008

>>>>>>> | | | | | | | | | | | | | | | | | | | | <<<<<<<

1827806 acttgccctggcaagtgccacggcaacggcttctgcgtggacggccgctgcgtgtgcatt 1827865

0001009 A G F S G E D C S Q L N C L N D C N G R 0001068

>>>>>>> | | | | | | | | | | | | | | | | | | | | <<<<<<<

1827866 gctggcttcagtggcgaagactgctcccagctcaactgcctgaacgactgcaacggccga 1827925

0001069 G S C F N G L C I C E A G Y Q G E D C S 0001128

>>>>>>> | | | | | | | | | | | | | | | | | | | | <<<<<<<

1827926 ggcagctgcttcaatgggctgtgcatctgcgaggcgggctaccagggcgaagactgcagc 1827985

0001129 Q L A C L N N C N S R G Q C I N G Q C S 0001188

>>>>>>> | | | | | | | | | | | | | | | | | | | | <<<<<<<

1827986 cagctagcttgtctgaacaactgtaacagcagagggcagtgcatcaacgggcagtgctct 1828045

0001189 C D V G F Y G E D C A E L S C P N S C F 0001248

>>>>>>> | | | | | | | | | | | | | | | | | | | | <<<<<<<

1828046 tgcgacgtcggcttctacggcgaagactgcgccgagctgtcctgtcccaacagctgcttt 1828105

0001249 N R G R C V N G Q C V C E E G Y A G E D 0001308

>>>>>>> | | | | | | | | | | | | | | | | | | | | <<<<<<<

1828106 aacagggggcgctgcgtgaacggacagtgcgtgtgcgaggaaggctacgccggcgaggac 1828165

0001309 C R V A T C P S N C Y G R G K C S E G R 0001368

>>>>>>> | | | | | | | | | | | | | | | | | | | | <<<<<<<

1828166 tgccgggtcgcgacctgcccctctaactgctatggtcggggtaagtgctccgagggtcgc 1828225

0001369 C A C H T G F T G D G M Q Q T E L S Q Q 0001428

>>>>>>> | | | | | | | | | | | | | | | | | | | | <<<<<<<

1828226 tgcgcgtgccacacgggcttcaccggggacggaatgcagcaaactgagctgtcccaacag 1828285

0001429 L P E S R Q 0001446

>>>>>>> | | | | | | <<<<<<<

1828286 ctgccagaatcgcggcag 1828303

0001447 G F A G E D C S R K A C P N D C L A R G 0001506

>>>>>>> | | | | | | | | | | | | | | | | | | | | <<<<<<<

1828335 ggcttcgccggcgaggactgcagccggaaagcttgccccaacgactgcctggcgcggggc 1828394

0001507 H C H D G K C V C Q D G Y T G V D C S A 0001566

>>>>>>> | | | | | | | | | | | | | | | | | | | | <<<<<<<

1828395 cactgccacgatggcaagtgcgtctgccaggacggctacacgggggtcgactgctctgcc 1828454

0001567 L S C P A N C N H R G R C V N G R C A C 0001626

>>>>>>> | | | | | | | | | | | | | | | | | | | | <<<<<<<

1828455 ctctcctgccccgccaactgtaaccacagggggcgttgtgtgaatggaaggtgtgcgtgc 1828514

0001627 E S G F E G E S C E E R S C L N G C R G 0001686

>>>>>>> | | | | | | | | | | | | | | | | | | | | <<<<<<<

1828515 gagagcgggtttgaaggcgaaagctgcgaagagcggagctgcctcaacggctgccggggc 1828574

0001687 N G R C L S G Q C L C D E G Y V G E D C 0001746

>>>>>>> | | | | | | | | | | | | | | | | | | | | <<<<<<<

1828575 aacggtcgctgcctgagcggccagtgcctctgcgatgagggatacgtgggagaagactgc 1828634

0001747 S G 0001752

>>>>>>> | | <<<<<<<

1828635 tcagga 1828640

0001753 V S P P T D L V V S E V T S D T V D L L 0001812

>>>>>>> | | | | | | | | | | | | | | | | | | | | <<<<<<<

1828878 gtgtctcctcccaccgaccttgtggtctccgaggtcaccagcgacaccgtggatctgctc 1828937

0001813 W R N Q M L V T E Y L V T Y A P T R A G 0001872

>>>>>>> | | | | | | | | | | | | | | | | | | | | <<<<<<<

1828938 tggcgcaaccagatgttggtgacggagtacctggtgacgtacgcccccaccagagccggc 1828997

0001873 G L L Q E F T V S G D K T A A T V H E L 0001932

>>>>>>> | | | | | | | | | | | | | | | | | | | | <<<<<<<

1828998 ggtctcctccaggagttcaccgtgtcaggggataaaactgcagccacggtgcacgagctc 1829057

0001933 E P G L E Y I I N V Y A I L S N K R S V 0001992

>>>>>>> | | | | | | | | | | | | | | | | | | | | <<<<<<<

1829058 gaaccaggcctagagtacataatcaatgtttacgccatcctgagcaacaagaggagcgtc 1829117

0001993 P I S A R V A T 0002016

>>>>>>> | | | | | | | | <<<<<<<

1829118 cccattagcgccagggtggctaca 1829141

0002017 D F P R P E G V I F K S V S E T S V E V 0002076

>>>>>>> | | | | | | | | | | | | | | | | | | | | <<<<<<<

1829217 gattttccccggcccgagggtgtgatcttcaaatctgtgagcgagacctcggtggaagtc 1829276

0002077 R W D Q L D I P F D G W E I F F R N T 0002133

>>>>>>> | | | | | | | | | | | | | | | | | | | <<<<<<<

1829277 aggtgggaccagctggacatccccttcgatggctgggaaatctttttccgcaacacg 1829333

0002134 K E E N G H I K T T V P S S Q N Q F V Q 0002193

>>>>>>> | | | | | | | | | | | | | | | | | | | | <<<<<<<

1829713 aaagaagaaaacggtcacatcaagaccaccgttccatcttctcagaaccagtttgtccag 1829772

0002194 S G L G P G Q E Y E I A I N M V R N N T 0002253

>>>>>>> | | | | | | | | | | | | | | | | | | | | <<<<<<<

1829773 tcaggtctcggaccaggacaggagtacgaaattgccatcaacatggttaggaacaacacc 1829832

0002254 R G P Q T T R T V T T 0002286

>>>>>>> | | | | | | | | | | | <<<<<<<

1829833 aggggaccccagaccaccagaacagtcaccacc 1829865

0002290 I D A P Q Q V E V K D V T D T S A L V S 0002349

>>>>>>> | | | | | | | | | | | | | | | | | | | | <<<<<<<

1830369 atcgacgccccccagcaggtggaggtgaaggacgtgacggacacctcggctctggttagc 1830428

0002350 W S Q P V A S V H R I T M F Y G L I S D 0002409

>>>>>>> | | | | | | | | | | | | | | | | | | | | <<<<<<<

1830429 tggtcgcagccggtggcatctgtgcacaggatcaccatgttttacgggctgatctccgac 1830488

0002410 P S D T N S V E I F P P D K Q Y S V D G 0002469

>>>>>>> | | | | | | | | | | | | | | | | | | | | <<<<<<<

1830489 ccctctgacacgaacagcgtggagatattcccccccgacaaacagtacagcgtggacggt 1830548

0002470 L R P D T E Y K V T L I S R R G D S T S 0002529

>>>>>>> | | | | | | | | | | | | | | | | | | | | <<<<<<<

1830549 ctgagaccagacaccgagtacaaggtgacgctcatctccaggaggggagactccaccagt 1830608

0002530 D P V S T S F I T 0002556

>>>>>>> | | | | | | | | | <<<<<<<

1830609 gaccccgtcagcacctcttttatcacc 1830635

0002557 A L D A P T N L Q I T S Q T D Q S I T V 0002616

>>>>>>> | | | | | | | | | | | | | | | | | | | | <<<<<<<

1830741 gcccttgatgctcccacaaacctgcagattacgtcccagacagaccagagcatcactgtt 1830800

0002617 Q W T N S K A N V S S Y L V K Y S P I S 0002676

>>>>>>> | | | | | | | | | | | | | | | | | | | | <<<<<<<

1830801 cagtggaccaacagcaaggctaacgttagcagctatctggtgaagtacagccccatttct 1830860

0002677 G D G H G E E L F P R Q P G Y S T K A A 0002736

>>>>>>> | | | | | | | | | | | | | | | | | | | | <<<<<<<

1830861 ggagacggtcacggcgaagagctgttcccacgtcaaccgggatactccaccaaagctgct 1830920

0002737 L T 0002742

>>>>>>> | | <<<<<<<

1830921 ctgact 1830926

0002743 G L R P G T E Y G I G V T A V K N E A E 0002802

>>>>>>> | | | | | | | | | | | | | | | | | | | | <<<<<<<

1830997 gggctgaggcctggaaccgaatacggaatcggtgtgaccgctgtgaagaacgaggcggag 1831056

0002803 S L P A T T N A E T 0002832

>>>>>>> | | | | | | | | | | <<<<<<<

1831057 agccttccagctaccaccaacgctgaaact 1831086

0002833 E I D P P R N V E D V E S S E T S L T L 0002892

>>>>>>> | | | | | | | | | | | | | | | | | | | | <<<<<<<

1831166 gaaatcgaccctcccaggaacgtggaggatgtggagtcctcagagacgtccctcaccctg 1831225

0002893 R W Q K P Q A K I S T Y R L V Y V S R D 0002952

>>>>>>> | | | | | | | | | | | | | | | | | | | | <<<<<<<

1831226 aggtggcagaaacctcaggccaagatcagcacctacaggctggtgtacgtctccagggac 1831285

0002953 G Q V E E E L I P A S A T S Y V M S N L 0003012

>>>>>>> | | | | | | | | | | | | | | | | | | | | <<<<<<<

1831286 gggcaggtggaggaggagctgatcccggcctcggcgacctcctacgtcatgtccaacctg 1831345

0003013 T P G M S Y S V T L T A E R G P R R S R 0003072

>>>>>>> | | | | | | | | | | | | | | | | | | | | <<<<<<<

1831346 actcctgggatgagctacagcgtgactctgacagcagagaggggtccgaggaggagcaga 1831405

0003073 P V S H S A S T 0003096

>>>>>>> | | | | | | | | <<<<<<<

1831406 ccagtgtcccattctgcgtccaca 1831429

0003097 E E L Q P A V T N L T V S D R T W D G F 0003156

>>>>>>> | | | | | | | | | | | | | | | | | | | | <<<<<<<

1832744 gaggagctgcagccggcagtgaccaacctcaccgtctctgacaggacgtgggatggcttc 1832803

0003157 T V S W T P A G G D F D S F V L E I T N 0003216

>>>>>>> | | | | | | | | | | | | | | | | | | | | <<<<<<<

1832804 actgtgtcctggacccccgcaggtggggactttgacagctttgtccttgagataacaaac 1832863

0003217 L E N L E E S Q N L T L S G G A L S L A 0003276

>>>>>>> | | | | | | | | | | | | | | | | | | | | <<<<<<<

1832864 ctggagaaccttgaggagagccagaacctgacgctctccggaggagcgctcagcctggcc 1832923

0003277 V S G L N P N T S Y V V G L F G V Y Q D 0003336

>>>>>>> | | | | | | | | | | | | | | | | | | | | <<<<<<<

1832924 gtgtccggcctgaaccccaacaccagctacgtggttggactgttcggggtgtatcaggac 1832983

0003337 S V L E P V Y T E A T T 0003372

>>>>>>> | | | | | | | | | | | | <<<<<<<

1832984 tccgttcttgaacccgtgtatactgaggccaccaca 1833019

0003373 E D V P Q L G L L T T S S V G P H D V S 0003432

>>>>>>> | | | | | | | | | | | | | | | | | | | | <<<<<<<

1834449 gaggacgtcccacagttggggctcctaacgacctcatctgtgggtccgcacgacgtcagc 1834508

0003433 L T W A T L S G H F D A F V V R I S D P 0003492

>>>>>>> | | | | | | | | | | | | | | | | | | | | <<<<<<<

1834509 ctgacctgggccacgctgtcgggccactttgatgcctttgtggtccgtatcagtgacccc 1834568

0003493 E Q Q F D T Q E Y T L Q R G A R N F T V 0003552

>>>>>>> | | | | | | | | | | | | | | | | | | | | <<<<<<<

1834569 gagcagcagtttgacacgcaggagtacacactccagcgtggggcccgtaactttaccgtc 1834628

0003553 S D L V D A T P Y D F E L Y G I S H D R 0003612

>>>>>>> | | | | | | | | | | | | | | | | | | | | <<<<<<<

1834629 tctgacctggtggacgccacgccgtatgactttgaactgtacggtatctctcatgaccgc 1834688

0003613 R T P S V F A H A V T 0003645

>>>>>>> | | | | | | | | | | | <<<<<<<

1834689 cgcactccctctgtgtttgcccatgctgtcaca 1834721

0003646 A P L P R V E N M T V S Q V T P H G F R 0003705

>>>>>>> | | | | | | | | | | | | | | | | | | | | <<<<<<<

1834927 gctccgttacctagagtggaaaatatgaccgtttcccaggtaactccccatggcttccgc 1834986

0003706 V S W E V N Q H L R Q E D L A P S S G H 0003765

>>>>>>> | | | | | | | | | | | | | | | | | | | | <<<<<<<

1834987 gtgtcatgggaggtgaatcagcacctgcggcaggaggatttagccccctctagcggccac 1835046

0003766 F R H F H I V V T D S G W L L E P Q E F 0003825

>>>>>>> | | | | | | | | | | | | | | | | | | | | <<<<<<<

1835047 tttcgccattttcacatagtggtgacagactcgggctggctgctggaaccgcaggagttc 1835106

0003826 S V P G N Q T H L D V T G L I T G I G Y 0003885

>>>>>>> | | | | | | | | | | | | | | | | | | | | <<<<<<<

1835107 tccgtgccggggaaccaaactcacctggatgtcacgggccttatcaccggcatcggctat 1835166

0003886 E V R L T G V S E S G L L S R P L T T V 0003945

>>>>>>> | | | | | | | | | | | | | | | | | | | | <<<<<<<

1835167 gaggtcaggctgaccggcgtgtccgagtcagggctcctctctcggcccctgaccacagtg 1835226

0003946 A V T 0003954

>>>>>>> | | | <<<<<<<

1835227 gctgtgaca 1835235

0003955 V A E P E V E H L F V S D I T D G G F R 0004014

>>>>>>> | | | | | | | | | | | | | | | | | | | | <<<<<<<

1835938 gtggctgagccggaggtggaacatctgtttgtctcggatatcacggacggcggtttccgc 1835997

0004015 L S W T S D E D M F D R F V V K I R D G 0004074

>>>>>>> | | | | | | | | | | | | | | | | | | | | <<<<<<<

1835998 ttgtcctggacttctgatgaagacatgtttgacagatttgtggtcaaaataagagacggc 1836057

0004075 K R L G H P R E Y A V R G D E R T M V V 0004134

>>>>>>> | | | | | | | | | | | | | | | | | | | | <<<<<<<

1836058 aaaagattaggtcaccctcgggagtacgccgtccgcggcgacgagcggaccatggtggta 1836117

0004135 T G L M S G A E Y E I E L Y G V A L D K 0004194

>>>>>>> | | | | | | | | | | | | | | | | | | | | <<<<<<<

1836118 accggactcatgagtggcgccgagtacgaaatcgagctttacggtgtcgcgttagacaaa 1836177

0004195 R S Q P V F G V A H T 0004227

>>>>>>> | | | | | | | | | | | <<<<<<<

1836178 cgctcgcaacccgtttttggagtcgctcacaca 1836210

0004228 G L S T P K G L Y F S D V T D S S A V V 0004287

>>>>>>> | | | | | | | | | | | | | | | | | | | | <<<<<<<

1836489 ggtctgagcactccgaagggcctgtatttctcagatgtgaccgactcctcggctgtggtt 1836548

0004288 H W S T P R S A V D S Y R V T Y V P F E 0004347

>>>>>>> | | | | | | | | | | | | | | | | | | | | <<<<<<<

1836549 cactggtccacgcctcggtctgcggtggacagctaccgcgtcacctacgtgccctttgaa 1836608

0004348 G 0004350

>>>>>>> | <<<<<<<

1836609 gga 1836611

0004351 G S P L T V T V D G G V F E A L L A N M 0004410

>>>>>>> | | | | | | | | | | | | | | | | | | | | <<<<<<<

1836702 ggcagcccgctgacggtgacggtggacggcggcgtgttcgaggctctgctcgccaacatg 1836761

0004411 I P G R K Y Q V T V S S V K G L E E S D 0004470

>>>>>>> | | | | | | | | | | | | | | | | | | | | <<<<<<<

1836762 attccaggcagaaagtaccaggtgacggtgagttccgtcaagggtctggaggagagcgac 1836821

0004471 P S M D T V T T 0004494

>>>>>>> | | | | | | | | <<<<<<<

1836822 cccagcatggacaccgttaccaca 1836845

0004495 A L D R P R T L T A L N V T D T S A L L 0004554

>>>>>>> | | | | | | | | | | | | | | | | | | | | <<<<<<<

1836925 gctttggaccggccgcggactctgacggcgctcaatgtcaccgacacctcggccctcctg 1836984

0004555 L W Q P C A A A V D G Y V V T Y S A E A 0004614

>>>>>>> | | | | | | | | | | | | | | | | | | | | <<<<<<<

1836985 ctgtggcagccgtgcgcagccgccgtggacggctacgtcgtcacctacagcgccgaagcc 1837044

0004615 V P P V V E H V S G S T V E F E M G S L 0004674

>>>>>>> | | | | | | | | | | | | | | | | | | | | <<<<<<<

1837110 gtgccccccgtcgtggagcacgtctctgggagcacggtggagttcgagatgggatctctg 1837169

0004675 A P G T R Y K V G V H A V K E A L K S N 0004734

>>>>>>> | | | | | | | | | | | | | | | | | | | | <<<<<<<

1837170 gctccgggaacgcgctataaagtcggagtccacgcggtaaaggaagccctgaagagcaac 1837229

0004735 P A I T E F T 0004755

>>>>>>> | | | | | | | <<<<<<<

1837230 cccgccattaccgagttcacc 1837250

0004756 T D V D P P R D L K A I N I Q T D G A T 0004815

>>>>>>> | | | | | | | | | | | | | | | | | | | | <<<<<<<

1837323 acagatgtggaccctcctcgggatctgaaggccatcaacatacagactgacggcgcgact 1837382

0004816 L T W K P P Q A A V T G Y T L T F T A G 0004875

>>>>>>> | | | | | | | | | | | | | | | | | | | | <<<<<<<

1837383 ctgacctggaaaccaccgcaggccgccgtcaccggctacaccctcaccttcacggccggc 1837442

0004876 G V I R 0004887

>>>>>>> | | | | <<<<<<<

1837443 ggtgtgatcagg 1837454

0004888 E V V L S P T A S S Y T M A Q L A G S T 0004947

>>>>>>> | | | | | | | | | | | | | | | | | | | | <<<<<<<

1837537 gaggtggtgctgagtccaacagcctcttcctacaccatggctcagctggctggctccacg 1837596

0004948 E Y N I R L Q A I A G A Q R S R H V D A 0005007

>>>>>>> | | | | | | | | | | | | | | | | | | | | <<<<<<<

1837597 gagtacaacatcaggctccaggccatcgccggggcccagcggagccgccatgtggacgcc 1837656

0005008 V F M T 0005019

>>>>>>> | | | | <<<<<<<

1837657 gtcttcatgacc 1837668

0005023 G Q L F A R P R D C A Q I R L N G E A V 0005082

>>>>>>> | | | | | | | | | | | | | | | | | | | | <<<<<<<

1837739 gggcagttgttcgcgcggccgcgggactgcgcccagattaggctgaacggagaggcggtt 1837798

0005083 S G L F S I Y V G G E E N Q P L Q V W C 0005142

>>>>>>> | | | | | | | | | | | | | | | | | | | | <<<<<<<

1837799 tctggcctgttctccatctacgtgggtggggaggagaaccagcccctccaggtttggtgt 1837858

0005143 D M T T D G G G W M 0005172

>>>>>>> | | | | | | | | | | <<<<<<<

1837859 gacatgaccacagacggtggaggatggatg 1837888

0005173 V F L R R Q S G K L D F F R N W K N Y T 0005232

>>>>>>> | | | | | | | | | | | | | | | | | | | | <<<<<<<

1838002 gttttcctcagacgccagagtgggaagctggatttcttcaggaactggaagaactacacc 1838061

0005233 A G F G N M N D E F W 0005265

>>>>>>> | | | | | | | | | | | <<<<<<<

1838062 gccggctttgggaacatgaacgatgagttctgg 1838094

0005266 L G L S N L H K I T S S G L Y E L R V D 0005325

>>>>>>> | | | | | | | | | | | | | | | | | | | | <<<<<<<

1838165 ttaggtctgagcaaccttcacaagataacgagttctggcctctacgagctgcgtgtggac 1838224

0005326 L R D S G E S A F A Q Y D K F V V A E P 0005385

>>>>>>> | | | | | | | | | | | | | | | | | | | | <<<<<<<

1838225 ctgagggacagcggcgagtcggcctttgctcagtatgacaagtttgtggtggctgagccg 1838284

0005386 R T R Y K L Y I G A Y S G T A 0005430

>>>>>>> | | | | | | | | | | | | | | | <<<<<<<

1838285 agaacgcgctacaagctgtacatcggagcctacagtggaacagca 1838329

0005431 G D S M T Y H Q G R P F S T Y D N D N D 0005490

>>>>>>> | | | | | | | | | | | | | | | | | | | | <<<<<<<

1838473 ggtgactccatgacgtaccatcagggtcgacccttctctacctacgacaatgacaacgac 1838532

0005491 I A V T N C A L S Y K G A F W Y K N C H 0005550

>>>>>>> | | | | | | | | | | | | | | | | | | | | <<<<<<<

1838533 atcgccgtcaccaactgcgccctgtcctacaaaggcgccttctggtataaaaactgtcac 1838592

0005551 R V N L M G K Y G D K S H S K 0005595

>>>>>>> | | | | | | | | | | | | | | | <<<<<<<

1838593 cgcgtcaacctcatgggcaaatatggtgacaaaagtcacagcaag 1838637

0005596 G I N W F H W R G H E H S I E F A E M K 0005655

>>>>>>> | | | | | | | | | | | | | | | | | | | | <<<<<<<

1838916 gggataaactggttccactggaggggccacgaacactcaattgaatttgcagagatgaaa 1838975

0005656 L R P A D F Q R P E S R R K R S 0005703

>>>>>>> | | | | | | | | | | | | | | | | <<<<<<<

1838976 cttcggccggccgactttcaaaggcccgagagcaggaggaagcggtcg 1839023

Tetraodon tenascin-W:

0000001 M T H S P L R R V L L L L G L V Y A M S 0000060

>>>>>>> | | | | | | | | | | | | | | | | | | | | <<<<<<<

5911200 atgacccacagtccgctgcggagggtcctgttgctgctgggtctcgtctacgccatgtca 5911141

0000061 L F S A A T N D P R A S S S E R G V T F 0000120

>>>>>>> | | | | | | | | | | | | | | | | | | | | <<<<<<<

5911140 ctcttcagcgccgccaccaatgacccgcgtgcctcgtcctccgagcggggagtcaccttc 5911081

0000121 S H V Y K I D V A E G S S C K T E A P S 0000180

>>>>>>> | | | | | | | | | | | | | | | | | | | | <<<<<<<

5911080 agccatgtctataaaatagacgtggctgagggatccagctgcaaaactgaggccccgtct 5911021

0000181 S 0000183

>>>>>>> | <<<<<<<

5911020 agt 5911018

0000184 L Q T E T T P N G E N D L V F R H S I K 0000243

>>>>>>> | | | | | | | | | | | | | | | | | | | | <<<<<<<

5910748 ctacagacagaaactactccaaacggagagaacgacctcgtttttaggcacagcatcaag 5910689

0000244 L Q T P K C D C D E S E S F K S L L Y R 0000303

>>>>>>> | | | | | | | | | | | | | | | | | | | | <<<<<<<

5910688 ctgcagacgccaaaatgtgactgcgacgagtcagaaagcttcaagtctctcttgtacaga 5910629

0000304 V N G L E E E V T Y L K S Q C T Q G C C 0000363

>>>>>>> | | | | | | | | | | | | | | | | | | | | <<<<<<<

5910628 gttaatgggctggaggaagaagtcacctatttaaagagccagtgtactcagggatgctgc 5910569

0000364 G G G G A A 0000381

>>>>>>> | | | | | | <<<<<<<

5910568 ggtggaggcggtgctgca 5910551

0000382 G L D T S C G G H G V Y Q H E T C S C L 0000441

>>>>>>> | | | | | | | | | | | | | | | | | | | | <<<<<<<

5910210 ggcctggacacgagctgcggtggccacggtgtgtaccagcatgaaacctgcagctgtctt 5910151

0000442 C N P G W E G P D C S V S S C P D E C N 0000501

>>>>>>> | | | | | | | | | | | | | | | | | | | | <<<<<<<

5910150 tgcaacccggggtgggaaggcccggattgctctgtgtcctcctgtcctgacgagtgcaac 5910091

0000502 D N G R C V D G Q C V C H Q G Y T G D D 0000561

>>>>>>> | | | | | | | | | | | | | | | | | | | | <<<<<<<

5910090 gacaacggtagatgcgtggacggccagtgtgtgtgtcaccaaggctacacaggggatgac 5910031

0000562 C S Q V A C P G D C S D K G H C V D G R 0000621

>>>>>>> | | | | | | | | | | | | | | | | | | | | <<<<<<<

5910030 tgcagccaggtggcgtgtccgggtgactgcagcgacaagggacactgtgtggacggaagg 5909971

0000622 C V C F P H F T G D D C S I Q K C P N D 0000681

>>>>>>> | | | | | | | | | | | | | | | | | | | | <<<<<<<

5909970 tgcgtgtgcttcccacacttcaccggggacgactgcagcattcagaagtgtcccaatgac 5909911

0000682 C A G H G R C V D G Q C V C D E G L Y G 0000741

>>>>>>> | | | | | | | | | | | | | | | | | | | | <<<<<<<

5909910 tgcgccggtcatggccggtgcgtggacggccagtgcgtctgcgatgaaggcctttacgga 5909851

0000742 E D C S 0000753

>>>>>>> | | | | <<<<<<<

5909850 gaggactgttca 5909839

0000754 S V F G P Q G L R L V R L T D I S L L V 0000813

>>>>>>> | | | | | | | | | | | | | | | | | | | | <<<<<<<

5907771 tcagtgttcggccctcagggactacggttggttcggctgaccgacatctccctcctggtt 5907712

0000814 E W E P V S G A E H Y I L T Y H P K N D 0000873

>>>>>>> | | | | | | | | | | | | | | | | | | | | <<<<<<<

5907711 gaatgggagcctgtttctggggcagagcattacattttgacctatcaccccaaaaatgat 5907652

0000874 E H S L Q Q 0000891

>>>>>>> | | | | | | <<<<<<<

5907651 gagcattcactgcagcag 5907634

0000892 V Q V P H K N S Y L I S G L T P G V T Y 0000951

>>>>>>> | | | | | | | | | | | | | | | | | | | | <<<<<<<

5907560 gttcaagttccccacaagaactcctacctcatctctgggctgactccgggggtcacctac 5907501

0000952 I V Q V H A V I K E V R S E A D T I E A 0001011

>>>>>>> | | | | | | | | | | | | | | | | | | | | <<<<<<<

5907500 attgtccaggtgcacgccgtcatcaaggaagtgcgcagtgaagcagacacgattgaagca 5907441

0001012 T T 0001017

>>>>>>> | | <<<<<<<

5907440 accaca 5907435

0001018 D I S G I N D F Q V L G Q T E V S I Q V 0001077

>>>>>>> | | | | | | | | | | | | | | | | | | | | <<<<<<<

5906301 gatatttcaggcataaacgactttcaggttcttgggcagacagaggtgtcgatccaggtg 5906242

0001078 G W K N P P A E V D Y F R L T A T D P S 0001137

>>>>>>> | | | | | | | | | | | | | | | | | | | | <<<<<<<

5906241 ggctggaagaacccgccagctgaggtggactacttcagactgaccgccaccgacccgtcc 5906182

0001138 G Q E E E L S V Q R S Q E A R T K H T I 0001197

>>>>>>> | | | | | | | | | | | | | | | | | | | | <<<<<<<

5906181 ggacaggaggaggagctgagcgtgcagaggagccaggaagcacgcactaaacacacaatt 5906122

0001198 L 0001200

>>>>>>> | <<<<<<<

5906121 ttg 5906119

0001201 G L F P G T D Y Q I S V Q A V K G A V E 0001260

>>>>>>> | | | | | | | | | | | | | | | | | | | | <<<<<<<

5905979 ggtctgtttccaggaacggactaccagatttcggtgcaggccgtcaaaggagctgtggag 5905920

0001261 G K P S S V T G G T 0001290

>>>>>>> | | | | | | | | | | <<<<<<<

5905919 ggaaagccgtcttctgtcactggcggcaca 5905890

0001291 D I D V P G N L V A I D V T E D T V T L 0001350

>>>>>>> | | | | | | | | | | | | | | | | | | | | <<<<<<<

5903001 gacattgatgttccaggcaacctggtcgccattgatgtaacggaggacactgtcacgctg 5902942

0001351 S W D R V R A D V E G Y M L S Y T S A E 0001410

>>>>>>> | | | | | | | | | | | | | | | | | | | | <<<<<<<

5902941 tcatgggatcgagtccgtgccgatgtcgagggttacatgctgagctacacgtctgccgaa 5902882

0001411 G S S S D I P V G R Y S T S Y K L I G L 0001470

>>>>>>> | | | | | | | | | | | | | | | | | | | | <<<<<<<

5902881 ggctccagttcagacatcccagtgggacgttacagcacctcgtacaagctgatcggtttg 5902822

0001471 K P G V L Y N V Y I W A F K E D K V S K 0001530

>>>>>>> | | | | | | | | | | | | | | | | | | | | <<<<<<<

5902821 aagcctggagttctttacaacgtctacatctgggccttcaaggaagacaaagtcagcaaa 5902762

0001531 K S S T E A E T 0001554

>>>>>>> | | | | | | | | <<<<<<<

5902761 aagagttcaacagaggctgaaaca 5902738

0001555 I D A P S E L K A T D V S V D S S V L T 0001614

>>>>>>> | | | | | | | | | | | | | | | | | | | | <<<<<<<

5899672 atcgacgccccatctgagctgaaggcgacagacgtgtcggtcgattcttctgttctcacc 5899613

0001615 W V P P L A D I D G Y I L T Y E H E D G 0001674

>>>>>>> | | | | | | | | | | | | | | | | | | | | <<<<<<<

5899612 tgggttcctcctctcgctgacatcgacggctacatcctcacctatgaacatgaggacggc 5899553

0001675 E M K 0001683

>>>>>>> | | | <<<<<<<

5899552 gagatgaag 5899544

0001684 A V E K Q L G H S E S R F A L S G L E T 0001743

>>>>>>> | | | | | | | | | | | | | | | | | | | | <<<<<<<

5899131 gctgtggagaagcagcttggacacagcgagagcaggtttgcactgtccggcctggagacg 5899072

0001744 G Q R Y S V T I I A Y R G N K R S K A A 0001803

>>>>>>> | | | | | | | | | | | | | | | | | | | | <<<<<<<

5899071 ggccagaggtacagtgtcaccataatcgcctacagagggaacaagaggagcaaagcagcc 5899012

0001804 Q T 0001809

>>>>>>> | | <<<<<<<

5899011 cagacc 5899006

0001810 P V G T L Y P F P M D C L Q I M K N G N 0001869

>>>>>>> | | | | | | | | | | | | | | | | | | | | <<<<<<<

5898891 ccagtcggtacgctctacccctttcccatggactgtcttcagatcatgaagaacgggaac 5898832

0001870 K K S G I F T V Y V N N D R S K P V E A 0001929

>>>>>>> | | | | | | | | | | | | | | | | | | | | <<<<<<<

5898831 aaaaagagcggcatttttacagtctatgttaacaatgaccgctccaaacctgtggaggcc 5898772

0001930 Y C D M E T D G G G W L 0001965

>>>>>>> | | | | | | | | | | | | <<<<<<<

5898771 tactgcgatatggagacggacggagggggctggctg 5898736

0001966 V L Q R R T S G K L D F L K R W R Q Y L 0002025

>>>>>>> | | | | | | | | | | | | | | | | | | | | <<<<<<<

5898637 gttctccaaagacgtacaagtggcaagctggactttctgaagcgctggagacagtaccta 5898578

0002026 A G F G N M T D E F W I 0002061

>>>>>>> | | | | | | | | | | | | <<<<<<<

5898577 gcagggtttggcaacatgactgatgagttctggata 5898542

0002062 G L D N I Y E L T N T P T R Y E L R F D 0002121

>>>>>>> | | | | | | | | | | | | | | | | | | | | <<<<<<<

5898460 ggtctggacaacatctatgagctcaccaacactcccactcgctatgagctgaggtttgat 5898401

0002122 L G L G P D R A Y A V Y D N F K I A S A 0002181

>>>>>>> | | | | | | | | | | | | | | | | | | | | <<<<<<<

5898400 ctgggcctggggcccgacagggcctacgccgtttacgacaacttcaagattgcgtcggcc 5898341

0002182 K Q K F K L T I G K Y S G T A 0002226

>>>>>>> | | | | | | | | | | | | | | | <<<<<<<

5898340 aaacagaagttcaaattgaccattggcaaatacagcggcacagca 5898296

0002227 G D A M T Y H Q G Q S W T T V D S D N D 0002286

>>>>>>> | | | | | | | | | | | | | | | | | | | | <<<<<<<

5897692 ggtgatgctatgacctaccaccaaggccagtcctggactaccgttgactctgacaatgac 5897633

0002287 I A L S N C A L S H R G A W W Y K N C H 0002346

>>>>>>> | | | | | | | | | | | | | | | | | | | | <<<<<<<

5897632 atcgccctgagcaactgcgccctgagccaccgtggcgcctggtggtacaagaactgccac 5897573

0002347 L A N L N G N W G D N R H S M 0002391

>>>>>>> | | | | | | | | | | | | | | | <<<<<<<

5897572 ctggccaacctcaacggcaactggggagacaacaggcacagcatg 5897528

0002392 G I N W K P W K G H L L S L D F T E M K 0002451

>>>>>>> | | | | | | | | | | | | | | | | | | | | <<<<<<<

5896324 ggcatcaactggaagccatggaagggccacctcttgtcgctcgatttcacggagatgaag 5896265

0002452 I R P V 0002463

>>>>>>> | | | | <<<<<<<

5896264 atccgaccggtg 5896253

Tetraodon tenascin-X:

0000001 M T H K I S L L P G G C S G G C E T E M 0000060

>>>>>>> | | | | | | | | | | | | | | | | | | | | <<<<<<<

6279952 atgacccataagatcagcttgctgcctggtggctgttcaggggggtgtgagaccgagatg 6279893

0000061 T A L K E R V A R L E R E M A S F K D N 0000120

>>>>>>> | | | | | | | | | | | | | | | | | | | | <<<<<<<

6279892 actgccctgaaggagcgtgtggcccgtttggaaagagagatggcctccttcaaagataac 6279833

0000121 C 0000123

>>>>>>> | <<<<<<<

6279832 tgt 6279830

0000124 P C S A N C P N N C S G N G E C Q K G K 0000183

>>>>>>> | | | | | | | | | | | | | | | | | | | | <<<<<<<

6279762 ccgtgttctgccaactgtccaaacaactgtagtggcaatggggagtgccagaaggggaaa 6279703

0000184 C F C Q D G F T G P D C S K C V Q G V E 0000243

>>>>>>> | | | | | | | | | | | | | | | | | | | | <<<<<<<

6279702 tgtttctgccaggatgggttcacaggtccagactgtagcaagtgtgtacaaggagttgag 6279643

0000244 C M K S K L E K N G T K L Y E F L K E S 0000303

>>>>>>> | | | | | | | | | | | | | | | | | | | | <<<<<<<

6279642 tgtatgaaaagtaagttggaaaaaaatggaacaaaattatatgaatttcttaaagagtcc 6279583

0000304 L I C T T D V A E A V K G K A K P A A E 0000363

>>>>>>> | | | | | | | | | | | | | | | | | | | | <<<<<<<

6279582 ttgatctgtactactgatgttgcagaagctgtcaaagggaaggccaaaccagcagcagag 6279523

0000364 T V M V Q G E K D K N S V Q E K T S K G 0000423

>>>>>>> | | | | | | | | | | | | | | | | | | | | <<<<<<<

6279522 acagtgatggtgcaaggagaaaaagacaaaaacagcgtccaggagaaaacgtctaaagga 6279463

0000424 E S T L S Q G K E Q K K A T E G E N T L 0000483

>>>>>>> | | | | | | | | | | | | | | | | | | | | <<<<<<<

6279462 gagagcactctgtcacaggggaaggagcagaagaaggctactgaaggagagaacactctc 6279403

0000484 S P G K E Q K K A T E G E N T L S P G K 0000543

>>>>>>> | | | | | | | | | | | | | | | | | | | | <<<<<<<

6279402 tcaccagggaaagaacagaagaaggctactgaaggagagaacactctctcaccagggaaa 6279343

0000544 E Q K K V T E G K N T L S P G K E Q K K 0000603

>>>>>>> | | | | | | | | | | | | | | | | | | | | <<<<<<<

6279342 gaacagaagaaggttactgaaggaaagaacactctctcaccagggaaggagcagaagaag 6279283

0000604 A T E G E N T L S P G K E Q K K V T E G 0000663

>>>>>>> | | | | | | | | | | | | | | | | | | | | <<<<<<<

6279282 gctactgaaggagagaacactctctcaccagggaaagaacagaagaaggttactgaagga 6279223

0000664 K N T L S P G K E Q K K A T E G E N T L 0000723

>>>>>>> | | | | | | | | | | | | | | | | | | | | <<<<<<<

6279222 aagaacactctctcaccagggaaggaacagaagaaggctactgaaggagagaacactctc 6279163

0000724 S P G K E Q K K V T E G K N T T A P G K 0000783

>>>>>>> | | | | | | | | | | | | | | | | | | | | <<<<<<<

6279162 tcaccagggaaggagcagaagaaggttactgaaggaaagaacactaccgcaccaggaaag 6279103

0000784 E Q K K T L E S K E A N T K P K V S T D 0000843

>>>>>>> | | | | | | | | | | | | | | | | | | | | <<<<<<<

6279102 gagcagaagaagactctggagagcaaagaagctaatactaaaccaaaagtgtctactgat 6279043

0000844 T K V S L K T Q P I Q E A S T K K T P T 0000903

>>>>>>> | | | | | | | | | | | | | | | | | | | | <<<<<<<

6279042 actaaagtgagtcttaaaacacaaccaatacaggaagcttccacaaaaaaaacacctact 6278983

0000904 K D S S G T T K T T R P T A G H V H L K 0000963

>>>>>>> | | | | | | | | | | | | | | | | | | | | <<<<<<<

6278982 aaagactcttctggcacaacaaagaccactcgcccaactgccggtcatgttcatttgaag 6278923

0000964 H D R A S Q E E S R E E K A V T A S K K 0001023

>>>>>>> | | | | | | | | | | | | | | | | | | | | <<<<<<<

6278922 catgatagagcaagccaagaggaatcccgcgaagaaaaagcagtgacagcttctaaaaag 6278863

0001024 V Q K P E V K L V R D E P T S K T H S S 0001083

>>>>>>> | | | | | | | | | | | | | | | | | | | | <<<<<<<

6278862 gtacaaaaacctgaagtcaaacttgtcagggatgaacctactagcaaaacacactccagc 6278803

0001084 S E Q I K D E P Q T N R T Q S S V K K S 0001143

>>>>>>> | | | | | | | | | | | | | | | | | | | | <<<<<<<

6278802 tccgaacaaataaaagatgaacctcaaacaaacagaacccagagtagtgtgaagaagtct 6278743

0001144 S G S S K I M T T L S K G V S A D K T K 0001203

>>>>>>> | | | | | | | | | | | | | | | | | | | | <<<<<<<

6278742 agtggttcctcaaaaatcatgactactttgtccaaaggtgttagtgctgataaaaccaaa 6278683

0001204 V E D S S E R P T L P E K S G I Q S A G 0001263

>>>>>>> | | | | | | | | | | | | | | | | | | | | <<<<<<<

6278682 gtagaggattcttcggagcggcctactctacctgagaagagtggtattcaatcagccggc 6278623

0001264 Q K D A K K V K V E A T T L Q S T D N K 0001323

>>>>>>> | | | | | | | | | | | | | | | | | | | | <<<<<<<

6278622 cagaaagatgccaagaaggtcaaagtagaagcaacaaccttacaatccactgacaacaaa 6278563

0001324 N T E A N K T V K D S E D N I S Q R S Q 0001383

>>>>>>> | | | | | | | | | | | | | | | | | | | | <<<<<<<

6278562 aatacagaagctaacaaaactgtaaaagattcagaagacaacatttctcaaaggagtcag 6278503

0001384 D V V N A T S V S T G S V R V L G G S G 0001443

>>>>>>> | | | | | | | | | | | | | | | | | | | | <<<<<<<

6278502 gatgtagttaatgctacatctgtctcaactgggagtgtgagggttctgggtggatctggg 6278443

0001444 L G S V K V A N I S S Y S F T F M W S A 0001503

>>>>>>> | | | | | | | | | | | | | | | | | | | | <<<<<<<

6278442 ttaggttcagtgaaggttgctaacatctcctcctacagctttactttcatgtggtcagca 6278383

0001504 P Q G M F K N F T L I R T E P L I E G T 0001563

>>>>>>> | | | | | | | | | | | | | | | | | | | | <<<<<<<

6278382 ccacaggggatgttcaagaacttcactttgatcaggacagaacctttgatcgaaggtacc 6278323

0001564 Q I D H E E F E E E T L E E V K T Y T V 0001623

>>>>>>> | | | | | | | | | | | | | | | | | | | | <<<<<<<

6278322 caaatcgaccatgaggagtttgaggaggagactctcgaagaagttaagacttacacagta 6278263

0001624 K N T A A R V Q V P T E S S N T T L G S 0001683

>>>>>>> | | | | | | | | | | | | | | | | | | | | <<<<<<<

6278262 aagaacaccgcagccagagtccaggtaccgactgaaagcagcaacacaactctcgggtct 6278203

0001684 G S R G K V E T R R I S M V I P G N I R 0001743

>>>>>>> | | | | | | | | | | | | | | | | | | | | <<<<<<<

6278202 gggtcaagaggcaaagtcgaaaccaggagaatctccatggtgatccctggaaacatacgc 6278143

0001744 S M E F S N L R P N T A Y V L Q I Y G S 0001803

>>>>>>> | | | | | | | | | | | | | | | | | | | | <<<<<<<

6278142 tctatggagttcagcaacctccggccaaacactgcctacgtcctgcaaatctatggcagc 6278083

0001804 T A Q R R S K I H R V A A V T 0001848

>>>>>>> | | | | | | | | | | | | | | | <<<<<<<

6278082 acagcacagaggaggtcaaagatccacagagtagctgcagtcaca 6278038

0001849 G P E P A T E M V F S N V T E S S L S V 0001908

>>>>>>> | | | | | | | | | | | | | | | | | | | | <<<<<<<

6277966 ggtccagagccagccacagagatggttttcagcaacgtgacagaatcatctctcagtgtt 6277907

0001909 S W S K P K T T Y A A F R I T Y T N I V 0001968

>>>>>>> | | | | | | | | | | | | | | | | | | | | <<<<<<<

6277906 tcctggtccaaaccaaagaccacttatgcggccttcaggatcacatacaccaacatcgtc 6277847

0001969 T 0001971

>>>>>>> | <<<<<<<

6277846 aca 6277844

0001972 G E Y Q F V N V N A Q E S H V V L T K L 0002031

>>>>>>> | | | | | | | | | | | | | | | | | | | | <<<<<<<

6277750 ggggagtaccagtttgtgaatgtgaacgctcaggagtctcatgtggttctcaccaaatta 6277691

0002032 S A G T S Y I V S V M A T Q G R A Q S D 0002091

>>>>>>> | | | | | | | | | | | | | | | | | | | | <<<<<<<

6277690 tctgctggaacctcctacattgtctctgtcatggctacacaaggcagagctcagagtgat 6277631

0002092 A L T S I I T T 0002115

>>>>>>> | | | | | | | | <<<<<<<

6277630 gcccttacctccatcattaccaca 6277607

0002116 V P A P P T H L R V V N V T D T K A L L 0002175

>>>>>>> | | | | | | | | | | | | | | | | | | | | <<<<<<<

6277439 gtacccgctcctccaacacatctgagagttgtcaacgtgacagacaccaaggctctgctg 6277380

0002176 Q W T P S L G K V D R F I I S Y E S S K 0002235

>>>>>>> | | | | | | | | | | | | | | | | | | | | <<<<<<<

6277379 caatggacaccaagtttgggcaaagtagaccgcttcattatcagctatgagtcctccaag 6277320

0002239 P N V T V N V M L A G N S V E H Q L R G 0002298

>>>>>>> | | | | | | | | | | | | | | | | | | | | <<<<<<<

6277241 cctaatgtgactgtgaatgtgatgttggctggaaactcggtggaacaccagctgagaggc 6277182

0002299 L Q R G T V Y T I K V L S Q K N S H Q S 0002358

>>>>>>> | | | | | | | | | | | | | | | | | | | | <<<<<<<

6277181 ctgcaaagaggcaccgtgtacaccatcaaggtcctgagtcagaagaacagtcaccagagc 6277122

0002359 M A V S T T F T T A 0002388

>>>>>>> | | | | | | | | | | <<<<<<<

6277121 atggccgtctccaccaccttcaccactgct 6277092

0002389 T L L L F S V V K A N E V G A R Y A V I 0002448

>>>>>>> | | | | | | | | | | | | | | | | | | | | <<<<<<<

6277036 actcttctgcttttctcagtggtcaaagccaacgaggttggtgcccgctacgcagtgatt 6276977

0002449 A W R T S T V I Y H S Y R L M Y Q V A G 0002508

>>>>>>> | | | | | | | | | | | | | | | | | | | | <<<<<<<

6276976 gcatggagaacctccactgtcatttaccacagctacaggctgatgtaccaggtggctgga 6276917

0002509 E E T K V K I 0002529

>>>>>>> | | | | | | | <<<<<<<

6276916 gaggagacgaaggtaaagatc 6276896

0002530 E V I L D G T M T E Y K L T G L L P V S 0002589

>>>>>>> | | | | | | | | | | | | | | | | | | | | <<<<<<<

6276827 gaggtgatcttggatggaaccatgacagagtacaagctgaccggcctgctgccagtgtca 6276768

0002590 R Y I V L V Q G E R E G R Y T S I V T T 0002649

>>>>>>> | | | | | | | | | | | | | | | | | | | | <<<<<<<

6276767 cgttacatcgttctggttcaaggagagagagaaggacgctacacatctattgttacaaca 6276708

0002650 E 0002652

>>>>>>> | <<<<<<<

6276707 gaa 6276705

0002653 L R F P F P S D C S Q E L L N G A L G S 0002712

>>>>>>> | | | | | | | | | | | | | | | | | | | | <<<<<<<

6276608 ctgcgtttccctttcccttctgattgctcccaggagcttctgaacggagctctggggtca 6276549

0002713 G E V D I Y P L G K E G R S V R V Y C D 0002772

>>>>>>> | | | | | | | | | | | | | | | | | | | | <<<<<<<

6276548 ggggaggtggacatctacccgttggggaaagagggtcggtctgtccgagtgtactgcgac 6276489

0002773 M E T D G G G W T 0002799

>>>>>>> | | | | | | | | | <<<<<<<

6276488 atggagactgatggtggcggctggaca 6276462

0002800 V F Q R R L N G K M D F Y R T W S E Y S 0002859

>>>>>>> | | | | | | | | | | | | | | | | | | | | <<<<<<<

6276378 gtgttccagaggaggcttaatggcaagatggatttctacagaacctggagtgaatacagc 6276319

0002860 A G F G N L S E E F W L 0002895

>>>>>>> | | | | | | | | | | | | <<<<<<<

6276318 gccggctttggaaacctcagtgaggagttctggctc 6276283

0002896 G N D V L Y N L T S V S P M S L R V D M 0002955

>>>>>>> | | | | | | | | | | | | | | | | | | | | <<<<<<<

6276206 ggaaacgatgttctctacaacctgaccagtgtcagccccatgagtctgagagtggatatg 6276147

0002956 R Y G N D T V Y A H Y A N F S I D S K E 0003015

>>>>>>> | | | | | | | | | | | | | | | | | | | | <<<<<<<

6276146 agatatggaaatgatacagtttatgctcactatgctaacttttcaattgactcaaaggag 6276087

0003016 R H Y A L T V S G Y T G T A 0003057

>>>>>>> | | | | | | | | | | | | | | <<<<<<<

6276086 aggcactacgctctcacagtgtctggttacaccggaactgca 6276045

0003058 G D S M R Y H N G R P F S A W D K N P D 0003117

>>>>>>> | | | | | | | | | | | | | | | | | | | | <<<<<<<

6275905 ggtgactcaatgaggtaccataacggtcgtccattctcagcttgggacaagaaccccgat 6275846

0003118 P L G I H C A R S Y M G G W W Y K N C Y 0003177

>>>>>>> | | | | | | | | | | | | | | | | | | | | <<<<<<<

6275845 cctttggggatccactgtgccaggtcttacatgggaggctggtggtacaagaactgctac 6275786

0003178 K T N L N G L Y D T N H N N 0003219

>>>>>>> | | | | | | | | | | | | | | <<<<<<<

6275785 aagaccaacctcaatggtctttacgacaccaaccataacaac 6275744

0003223 N S L D R L E G E R C L H S L Y R D E V 0003282

>>>>>>> | | | | | | | | | | | | | | | | | | | | <<<<<<<

6275647 aatagtctggatagactggaaggggaaagatgtctccattccctttaccgagatgaagtt 6275588

0003283 Q T I E V L S C N S R L N L 0003324

>>>>>>> | | | | | | | | | | | | | | <<<<<<<

6275587 cagaccattgaggttctctcctgcaactcacggctaaatctg 6275546

Tetraodon tenascin-R (partial):

0000013 G S S P A A P S A D N K L V R T T R V R 0000072

>>>>>>> | | | | | | | | | | | | | | | | | | | | <<<<<<<

5848751 ggttcctcccccgcagctccatcagcagacaacaagctggttcgtaccaccagggtgagg 5848810

0000073 R Q T L A G G Q A P P L S S E N Q T T R 0000132

>>>>>>> | | | | | | | | | | | | | | | | | | | | <<<<<<<

5848811 agacagacgttggcggggggccaggctccccccttgtcttcagaaaaccagaccacaagg 5848870

0000133 E Q P L V F N H V Y N I N V P L E S L C 0000192

>>>>>>> | | | | | | | | | | | | | | | | | | | | <<<<<<<

5848871 gaacaacctctggtgtttaatcacgtgtacaacatcaatgttcccctggagtccctgtgt 5848930

0000193 S V D L D S A A S A G P T D 0000234

>>>>>>> | | | | | | | | | | | | | | <<<<<<<

5848931 tctgtggacctggactctgctgcgtctgctggcccaactgac 5848972

0000235 D D V Y A P H Q H P E A A C G C P A T A 0000294

>>>>>>> | | | | | | | | | | | | | | | | | | | | <<<<<<<

5849243 gatgacgtttacgcaccgcatcaacatcccgaagctgcatgtggttgtccggcaactgcc 5849302

0000295 T I Q Q L V T R V E M L E K K V S L L K 0000354

>>>>>>> | | | | | | | | | | | | | | | | | | | | <<<<<<<

5849303 accatacagcagctggtgaccagggtggaaatgctggagaaaaaggtctccctgctcaaa 5849362

0000355 A Q C G S G C C S E G S A M 0000396

>>>>>>> | | | | | | | | | | | | | | <<<<<<<

5849363 gcccagtgtgggtcggggtgctgcagcgagggctcagctatg 5849404

0000397 G R V D F V P G C S G H G S F S F D L C 0000456

>>>>>>> | | | | | | | | | | | | | | | | | | | | <<<<<<<

5849921 ggtcgggtggactttgtcccgggctgcagtgggcacggcagcttcagttttgacctctgc 5849980

0000457 G C I C E E G W A G K N C S E P R C P D 0000516

>>>>>>> | | | | | | | | | | | | | | | | | | | | <<<<<<<

5849981 ggctgcatctgtgaggaaggctgggcgggtaagaactgctccgagccccgctgcccggac 5850040

0000517 D C S G Q G A C V E G E C V C D R D F G 0000576

>>>>>>> | | | | | | | | | | | | | | | | | | | | <<<<<<<

5850041 gactgctcgggccagggggcgtgcgtggaaggggagtgcgtgtgcgaccgcgacttcgga 5850100

0000577 G E N C S E P R C P S D C S G R G L C I 0000636

>>>>>>> | | | | | | | | | | | | | | | | | | | | <<<<<<<

5850101 ggagagaactgctcggagccgcggtgcccctccgactgctcgggccgggggctgtgcatc 5850160

0000637 D G E C V C E E S F T G E D C M V G R C 0000696

>>>>>>> | | | | | | | | | | | | | | | | | | | | <<<<<<<

5850161 gacggcgagtgtgtgtgcgaggagtccttcaccggggaggactgcatggtcgggaggtgc 5850220

0000697 L N D C S D Q G T C V N S T C Q C R P G 0000756

>>>>>>> | | | | | | | | | | | | | | | | | | | | <<<<<<<

5850221 ctgaacgactgctcggaccagggcacctgtgtcaacagcacgtgccagtgccggcccggc 5850280

0000757 Y V G E D C S L V Y C A N N C 0000801

>>>>>>> | | | | | | | | | | | | | | | <<<<<<<

5850281 tacgtcggggaagactgctcgctggtgtactgtgccaacaactgc 5850325

0000802 S P D L S N P D G L V F K S I T E T S V 0000861

>>>>>>> | | | | | | | | | | | | | | | | | | | | <<<<<<<

5855792 tctccagatctctccaatcctgatggtttagtcttcaaatccatcacggaaacatcggtg 5855851

0000862 E V Q W K P F Y Y S F D G W E I S F I P 0000921

>>>>>>> | | | | | | | | | | | | | | | | | | | | <<<<<<<

5855852 gaggtccagtggaaaccgttctactattccttcgatggctgggagatcagcttcatccca 5855911

0000922 K 0000924

>>>>>>> | <<<<<<<

5855912 aag 5855914

0000925 D N D G G M T A Q L P S T I T S F V Q T 0000984

>>>>>>> | | | | | | | | | | | | | | | | | | | | <<<<<<<

5858912 gacaatgatggagggatgacagcacagctgccgagcaccatcacttcttttgtgcagact 5858971

0000985 G L R P G E E Y T V N L V A L R D Q G R 0001044

>>>>>>> | | | | | | | | | | | | | | | | | | | | <<<<<<<

5858972 ggtctgagaccgggggaggagtacaccgtgaacctggtggccctccgagaccagggtcga 5859031

0001045 S Q P V T A T V T T 0001074

>>>>>>> | | | | | | | | | | <<<<<<<

5859032 agccagcctgtcacagccaccgtcaccaca 5859061

0001078 I D G P T Q L M V R D V S D T V A F V E 0001137

>>>>>>> | | | | | | | | | | | | | | | | | | | | <<<<<<<

5861387 attgatggacccacacagctgatggtgcgagatgtgtctgacaccgtagcctttgtggag 5861446

0001138 W T P P K A K L D Q I V L R Y G L V G E 0001197

>>>>>>> | | | | | | | | | | | | | | | | | | | | <<<<<<<

5861447 tggaccccacccaaagcaaagctcgatcaaatagtgttgcgctatggcttggtgggggaa 5861506

0001198 G P R T T F R L Q P T L S Q Y S 0001245

>>>>>>> | | | | | | | | | | | | | | | | <<<<<<<

5861507 ggtcctcggaccaccttccggctccagcctacactaagccagtactcc 5861554

0001246 L Q V L R P G S R Y E V S V T G V R T G 0001305

>>>>>>> | | | | | | | | | | | | | | | | | | | | <<<<<<<

5862184 ctgcaggttctgcgccctggttcgcgttacgaagtgtctgtgacaggtgtgcggacagga 5862243

0001306 N E S G S I S T E F T T 0001341

>>>>>>> | | | | | | | | | | | | <<<<<<<

5862244 aatgaaagcggatctatttcaacagaatttacaact 5862279

0001342 E I D A P K N L W L V S K T S T T L E L 0001401

>>>>>>> | | | | | | | | | | | | | | | | | | | | <<<<<<<

5862508 gaaatcgatgctcccaagaacctgtggctggtgtcaaagacctccaccaccctggagctg 5862567

0001402 E W D N S E A E 0001425

>>>>>>> | | | | | | | | <<<<<<<

5862568 gaatgggataacagcgaggctgag 5862591

0001426 V D G Y Q V V Y S T L A G E Q Y D K V I 0001485

>>>>>>> | | | | | | | | | | | | | | | | | | | | <<<<<<<

5862722 gtggacggctaccaggtggtttacagcaccttggcaggagaacaatacgataaagttatc 5862781

0001486 V P R N E G A T T K T A L 0001524

>>>>>>> | | | | | | | | | | | | | <<<<<<<

5862782 gttcctcgcaacgagggagcaaccactaaaaccgctctc 5862820

0001525 T D L L P G T E Y G I G I S A M K G S N 0001584

>>>>>>> | | | | | | | | | | | | | | | | | | | | <<<<<<<

5863469 acagacctgctgccgggcactgagtacggcatcggtatttctgctatgaaaggcagcaac 5863528

0001585 Q S T P A T M N A R T 0001617

>>>>>>> | | | | | | | | | | | <<<<<<<

5863529 cagagcacgccggcaacaatgaacgccagaaca 5863561

0001618 G L D V P M D L T V T A S T D N T I T L 0001677

>>>>>>> | | | | | | | | | | | | | | | | | | | | <<<<<<<

5863853 ggtttggacgtccccatggatctcaccgtgacagcttctacagacaacaccatcacattg 5863912

0001678 V W G V V Q G P I D H Y R V T C T S S S 0001737

>>>>>>> | | | | | | | | | | | | | | | | | | | | <<<<<<<

5863913 gtgtggggtgtggtccagggtcccatcgaccactacagggtcacatgcacatcctcctca 5863972

0001738 G V T T E 0001752

>>>>>>> | | | | | <<<<<<<

5863973 ggagtcaccactgag 5863987

0001753 L T V P K D V T T T T L A G L D P G T E 0001812

>>>>>>> | | | | | | | | | | | | | | | | | | | | <<<<<<<

5864058 ctgacagtgcccaaagatgttaccaccacaaccctggcagggctcgaccccgggactgag 5864117

0001813 Y T I T V A A R R G R Q Q S N V A T I D 0001872

>>>>>>> | | | | | | | | | | | | | | | | | | | | <<<<<<<

5864118 tacactatcactgtggcagcgagaagaggacggcaacaaagcaatgttgctacgattgac 5864177

0001873 A F T 0001881

>>>>>>> | | | <<<<<<<

5864178 gccttcaca 5864186

0001882 G I R P V T H L F L S E V T S D S V L V 0001941

>>>>>>> | | | | | | | | | | | | | | | | | | | | <<<<<<<

5866267 ggaatcaggcccgtaactcacctcttcttgtcagaagtcacgtcagactcggtgttggtg 5866326

0001942 A W S A P A P P A D L F I L S Y S S S D 0002001

>>>>>>> | | | | | | | | | | | | | | | | | | | | <<<<<<<

5866327 gcctggagcgccccggcaccacctgccgacctctttatcctgagctacagctcttccgat 5866386

0002002 G T D T S K V T L D G S K T A S L I Q G 0002061

>>>>>>> | | | | | | | | | | | | | | | | | | | | <<<<<<<

5866387 gggacagacacgtctaaggtgacgctggatggctctaagacagcatctctgatccagggg 5866446

0002062 L L P S T P Y T I S L I T I Q A D I T S 0002121

>>>>>>> | | | | | | | | | | | | | | | | | | | | <<<<<<<

5866447 ttgttgccatccacaccctacaccatcagtctaatcacaatacaggcggacatcacctct 5866506

0002122 E P I T 0002133

>>>>>>> | | | | <<<<<<<

5866507 gaacccattaca 5866518

0002155 I M V S D V T E D S V T L S W I R P L A 0002214

>>>>>>> | | | | | | | | | | | | | | | | | | | | <<<<<<<

5868225 ataatggtgtcagatgtgaccgaggactctgtgactctctcctggatcagacctctggct 5868284

0002215 P F E Y Y K L S Y Q S A R 0002253

>>>>>>> | | | | | | | | | | | | | <<<<<<<

5868285 ccgttcgaatactacaagctttcctaccagtcagccaga 5868323

0002254 G R V D S M V I D S D V T N Y T L S S L 0002313

>>>>>>> | | | | | | | | | | | | | | | | | | | | <<<<<<<

5868640 ggacgcgtggacagcatggtgattgacagcgacgtgaccaattacaccttgtccagcctt 5868699

0002314 F P A T E Y E I S L S A V K E S Q E S S 0002373

>>>>>>> | | | | | | | | | | | | | | | | | | | | <<<<<<<

5868700 ttccctgctacagaatatgagatcagcctcagtgctgtcaaagagagccaggagagcagc 5868759

0002374 V V S T S V F T 0002397

>>>>>>> | | | | | | | | <<<<<<<

5868760 gtggttagcacctctgtcttcaca 5868783

0002398 A M D M P S E L T A L N I T P R G A L L 0002457

>>>>>>> | | | | | | | | | | | | | | | | | | | | <<<<<<<

5870565 gcgatggacatgccctctgagttgacagctctgaacatcactccacgaggagccctgctg 5870624

0002458 R W N P P L S V V D N Y V L T L T H N Q 0002517

>>>>>>> | | | | | | | | | | | | | | | | | | | | <<<<<<<

5870625 aggtggaaccccccgctttccgtggtcgacaactatgtgctgaccctcacacacaatcaa 5870684

0002518 V T A D T F L V E G T K Q E H Q L S S L 0002577

>>>>>>> | | | | | | | | | | | | | | | | | | | | <<<<<<<

5870832 gtgacagctgacacgttcctggtggaaggcaccaagcaggagcaccagctgtccagcctg 5870891

0002578 S P S T S Y S V A L Y A T K G P L T S G 0002637

>>>>>>> | | | | | | | | | | | | | | | | | | | | <<<<<<<

5870892 agtcccagcaccagctactctgtggctctgtacgccaccaaaggaccgctgaccagcggc 5870951

0002638 T V I T N L Q T 0002661

>>>>>>> | | | | | | | | <<<<<<<

5870952 actgtgatcaccaacctccaaaca 5870975

0002665 M D A P L N L T A S E V N H R S A L I S 0002724

>>>>>>> | | | | | | | | | | | | | | | | | | | | <<<<<<<

5874265 atggatgcacctttgaacctgacagccagcgaggtcaaccaccgcagcgctctcatctcc 5874324

0002725 W Q P P M A E I D N Y M L T Y K S V D G 0002784

>>>>>>> | | | | | | | | | | | | | | | | | | | | <<<<<<<

5874325 tggcaaccaccgatggcagagattgataattacatgctcacgtacaagtcagtcgacggc 5874384

0002785 G R K 0002793

>>>>>>> | | | <<<<<<<

5874385 ggccgcaaa 5874393

0002794 E L I L D A E D T W I R L E G L A E I T 0002853

>>>>>>> | | | | | | | | | | | | | | | | | | | | <<<<<<<

5876472 gagctcatcctggatgctgaggacacatggatacgcctggaggggctggctgagatcaca 5876531

0002854 E Y T V K L Q A A R G L D T S A I V S T 0002913

>>>>>>> | | | | | | | | | | | | | | | | | | | | <<<<<<<

5876532 gagtacactgttaaactccaggctgccaggggtctcgacaccagcgccatcgtctcaact 5876591

0002914 T F I T 0002925

>>>>>>> | | | | <<<<<<<

5876592 acctttattaca 5876603

0002926 G S R L F A M P Q N C A Q H L L N G E T 0002985

>>>>>>> | | | | | | | | | | | | | | | | | | | | <<<<<<<

5876862 gggagtcgcctctttgccatgcctcagaactgtgctcagcacctcctaaacggtgagacg 5876921

0002986 L S G V Y T I Y I N R D P S Q G V Q V Y 0003045

>>>>>>> | | | | | | | | | | | | | | | | | | | | <<<<<<<

5876922 ctgagtggcgtttacaccatttacattaaccgagaccccagccagggtgtgcaggtgtac 5876981

0003046 C D M T T D D G G W I 0003078

>>>>>>> | | | | | | | | | | | <<<<<<<

5876982 tgcgacatgaccacggacgacggcggctggatt 5877014

0003079 V F Q R R Q N G L T D F S R K W S D Y R 0003138

>>>>>>> | | | | | | | | | | | | | | | | | | | | <<<<<<<

5877091 gtgttccagcggcgtcagaacggcctgaccgatttttccaggaagtggagcgactatcgt 5877150

0003139 V G F G N L E D E F W L 0003174

>>>>>>> | | | | | | | | | | | | <<<<<<<

5877151 gttgggtttggaaacctggaggacgaattctggctc 5877186

0003175 G L D N I Q R V A A Q G R Y E L R I D M 0003234

>>>>>>> | | | | | | | | | | | | | | | | | | | | <<<<<<<

5880046 ggcttagacaacatccagagggttgctgctcagggtcgttacgagctgcgaatcgacatg 5880105

0003235 K D G Q E S V Y A N Y D K F S I G D A R 0003294

>>>>>>> | | | | | | | | | | | | | | | | | | | | <<<<<<<

5880106 aaggacggacaggaatccgtctacgccaattatgataaattctccattggcgatgcaaga 5880165

0003295 D L Y K L R I G E Y N G T A 0003336

>>>>>>> | | | | | | | | | | | | | | <<<<<<<

5880166 gacctctacaagctcagaataggagagtacaatggaactgct 5880207

0003337 G D S L S Y H Q G R P F S T K D R D N D 0003396

>>>>>>> | | | | | | | | | | | | | | | | | | | | <<<<<<<

5880544 ggtgactctctgagctatcaccagggtcgccctttctccacaaaagacagggacaacgac 5880603

0003397 I A V T N C A L S Y K G A W W Y K N C H 0003456

>>>>>>> | | | | | | | | | | | | | | | | | | | | <<<<<<<

5880604 atcgctgtcactaactgtgccttgtcttacaaaggggcctggtggtacaagaactgccac 5880663

0003457 R A N L N G K Y G E S R H S 0003498

>>>>>>> | | | | | | | | | | | | | | <<<<<<<

5880664 cgggccaaccttaatggcaaatatggcgaatcaagacacagc 5880705

0003499 Q G I N W Y H W K G H E F S I P F V E M 0003558

>>>>>>> | | | | | | | | | | | | | | | | | | | | <<<<<<<

5881632 cagggtatcaactggtaccactggaaaggccacgagttctccatcccctttgtggagatg 5881691

0003559 K M R P F N Y R S I S S K R R R S 0003609

>>>>>>> | | | | | | | | | | | | | | | | | <<<<<<<

5881692 aagatgagacctttcaactaccgtagcatcagcagcaagcggaggcgctcc 5881742
