# Supplementary material for: Phylogenetic analysis of the tenascin gene family: evidence of origin early in the chordate lineage
Source: BMC Evol Biol. 2006 Aug 7;6:60. doi: 10.1186/1471-2148-6-60 (PMC1578592; doi:10.1186/1471-2148-6-60)
Supplement: Additional file 2 — Side-by-side alignment of predicted tenascins from Takifugu. [file 1471-2148-6-60-S2.doc]

Appendix 2: Side by side alignment of Takifugu tenascins.

Takifugu tenascin-W:

000000001 L S P E Q G V T F S H V Y K I D V A A G 000000060

>>>>>>>>> | | | | | | | | | | | | | | | | | | | | >>>>>>>>>

115879838 ttgtctcccgagcaaggagtcaccttcagccacgtttataaaatagacgtggctgcggga 115879897

000000061 S S C K S E D Q P S E G K 000000099

>>>>>>>>> | | | | | | | | | | | | | >>>>>>>>>

115879898 tccagctgcaaatccgaggaccagccttcggagggaaaa 115879936

000000100 T E A T S N G E N D I V F R H N I R L Q 000000159

>>>>>>>>> | | | | | | | | | | | | | | | | | | | | >>>>>>>>>

115880176 acagaagctacttcaaacggagagaacgacatcgtcttccggcacaacatcaggctccag 115880235

000000160 T P K C D C D E S E S L K S L M Y R I N 000000219

>>>>>>>>> | | | | | | | | | | | | | | | | | | | | >>>>>>>>>

115880236 accccaaaatgtgactgcgacgagtcagaaagcctcaagtctctcatgtacagaatcaat 115880295

000000220 G L E E E V T Y L K N Q C T Q G C C G G 000000279

>>>>>>>>> | | | | | | | | | | | | | | | | | | | | >>>>>>>>>

115880296 gggctggaagaagaagtcacctatttaaagaaccagtgtacccagggatgttgtggtgga 115880355

000000280 G G A I 000000291

>>>>>>>>> | | | | >>>>>>>>>

115880356 ggcggtgcaata 115880367

000000292 G L D T S C S G H G V Y Q Q E T C S C V 000000351

>>>>>>>>> | | | | | | | | | | | | | | | | | | | | >>>>>>>>>

115880543 ggcctggacacaagctgtagtggccacggtgtgtaccagcaagagacctgcagctgcgtg 115880602

000000352 C N L G W E G Q D C S L S S C P D E C N 000000411

>>>>>>>>> | | | | | | | | | | | | | | | | | | | | >>>>>>>>>

115880603 tgcaacctggggtgggaaggtcaagactgctccttgtcctcctgtcctgacgagtgcaac 115880662

000000412 D N G R C V D G R C V C H Q G Y T G D D 000000471

>>>>>>>>> | | | | | | | | | | | | | | | | | | | | >>>>>>>>>

115880663 gataacgggcggtgtgtggacggccggtgcgtgtgtcaccagggctacacaggggacgac 115880722

000000472 C N Q L T C L G D C N D K G Q C V D G K 000000531

>>>>>>>>> | | | | | | | | | | | | | | | | | | | | >>>>>>>>>

115880723 tgcaaccagttgacctgtctgggcgactgcaacgacaaggggcaatgcgtggatggaaag 115880782

000000532 C V C F P H F T G D D C S T Q K C P N N 000000591

>>>>>>>>> | | | | | | | | | | | | | | | | | | | | >>>>>>>>>

115880783 tgtgtgtgttttccacacttcacaggggacgactgcagcacccagaagtgtccgaacaat 115880842

000000592 C V G N G Q C V D G Q C I C D E G F Y G 000000651

>>>>>>>>> | | | | | | | | | | | | | | | | | | | | >>>>>>>>>

115880843 tgcgtcggtaacggccagtgcgtggacggccagtgcatctgtgatgaaggcttttatgga 115880902

000000652 E D C S 000000663

>>>>>>>>> | | | | >>>>>>>>>

115880903 gaagactgttca 115880914

000000664 S V F G P Q G L R L V Q L T D V S L L V 000000723

>>>>>>>>> | | | | | | | | | | | | | | | | | | | | >>>>>>>>>

115883824 tcagtctttggcccgcagggactgcggttggttcagctgaccgacgtctctctcctggtg 115883883

000000724 E W E P V L G A E Y Y I L T Y H S K N N 000000783

>>>>>>>>> | | | | | | | | | | | | | | | | | | | | >>>>>>>>>

115883884 gaatgggagcccgttcttggagcagagtattacattttgacctatcattccaaaaacaat 115883943

000000784 E R A L Q Q 000000801

>>>>>>>>> | | | | | | >>>>>>>>>

115883944 gagcgtgctttgcagcag 115883961

000000802 V Q V P N K K N S Y L I T G L S P G V T 000000861

>>>>>>>>> | | | | | | | | | | | | | | | | | | | | >>>>>>>>>

115884035 gttcaagttcctaacaagaagaactcttacctcatcaccgggctgtctcctggggtcacc 115884094

000000862 Y A V Q V Y A V I K E V R S E A D M I E 000000921

>>>>>>>>> | | | | | | | | | | | | | | | | | | | | >>>>>>>>>

115884095 tacgccgtccaggtgtacgcagtcatcaaggaagtgcgtagtgaagcggacatgattgag 115884154

000000922 A T T 000000930

>>>>>>>>> | | | >>>>>>>>>

115884155 gcgaccaca 115884163

000000931 D V S G I D E F Q V L G Q T E V S I Q V 000000990

>>>>>>>>> | | | | | | | | | | | | | | | | | | | | >>>>>>>>>

115885181 gatgtttcaggcatagatgagttccaagtcctggggcagacagaggtgtcgatccaggtg 115885240

000000991 G W K N P P A E V D Y F R L T T T D P A 000001050

>>>>>>>>> | | | | | | | | | | | | | | | | | | | | >>>>>>>>>

115885241 ggctggaagaacccgccagccgaagtagactacttcaggctcaccaccaccgaccccgcc 115885300

000001051 G Q E E E V N V Q R S Q E A R T K H T I 000001110

>>>>>>>>> | | | | | | | | | | | | | | | | | | | | >>>>>>>>>

115885301 ggacaggaagaggaagtgaacgtgcagaggagccaggaagcccgcacaaaacacacaatt 115885360

000001111 V 000001113

>>>>>>>>> | >>>>>>>>>

115885361 gtg 115885363

000001114 G L F P G T D Y Q I S V Q A V K G A T E 000001173

>>>>>>>>> | | | | | | | | | | | | | | | | | | | | >>>>>>>>>

115885491 ggactgtttccaggaactgactaccagatttcggtgcaggccgtcaaaggagccacggag 115885550

000001174 G K S S S L T A G T 000001203

>>>>>>>>> | | | | | | | | | | >>>>>>>>>

115885551 ggaaagtcttcttctctcactgcaggcaca 115885580

000001204 D I D A P T N L A T T D V T E D T I T V 000001263

>>>>>>>>> | | | | | | | | | | | | | | | | | | | | >>>>>>>>>

115888560 gacattgatgctccgaccaacctggcgaccactgacgtaacggaggacaccatcacggtg 115888619

000001264 S W D Q V Q S E V K G Y M L S Y A S V E 000001323

>>>>>>>>> | | | | | | | | | | | | | | | | | | | | >>>>>>>>>

115888620 tcatgggatcaagtccagtcggaagtcaagggttacatgctgagctacgcgtctgtcgag 115888679

000001324 G S S S E I P V G R D S T S Y R L I G L 000001383

>>>>>>>>> | | | | | | | | | | | | | | | | | | | | >>>>>>>>>

115888680 ggctccagttcagagattcccgtgggacgtgacagcacctcgtataggctgatcggtctg 115888739

000001384 K P G V L H N I Y I W A F K E D K V S K 000001443

>>>>>>>>> | | | | | | | | | | | | | | | | | | | | >>>>>>>>>

115888740 aagcccggagttctccacaacatctacatctgggccttcaaggaagacaaagtcagcaaa 115888799

000001444 K S S T D A E T 000001467

>>>>>>>>> | | | | | | | | >>>>>>>>>

115888800 aagagttcaacagatgctgaaaca 115888823

000001468 E L D A P A N L L A Q D K T E S S F S V 000001527

>>>>>>>>> | | | | | | | | | | | | | | | | | | | | >>>>>>>>>

115891142 gaactggatgctcctgctaacctcttagcacaagacaaaacagagtccagcttcagcgtg 115891201

000001528 S W N P V R A D I D G Y I L T Y G S S E 000001587

>>>>>>>>> | | | | | | | | | | | | | | | | | | | | >>>>>>>>>

115891202 tcatggaatccagtgcgtgcggacattgatggctacatcctcacctacggctcctctgag 115891261

000001588 G S N Q E I P V G P D S T S Y R L T G L 000001647

>>>>>>>>> | | | | | | | | | | | | | | | | | | | | >>>>>>>>>

115891262 ggctcaaaccaagaaataccagtcggacctgacagcacctcttacaggctgaccggtctg 115891321

000001648 R P G V L Y T V Y I W A F K D N K T T K 000001707

>>>>>>>>> | | | | | | | | | | | | | | | | | | | | >>>>>>>>>

115891322 aggcctggcgtcctctacacagtctacatctgggccttcaaagacaacaaaaccactaag 115891381

000001708 T I A T K A E 000001728

>>>>>>>>> | | | | | | | >>>>>>>>>

115891382 acgattgcgacaaaagctgag 115891402

000001729 T D I D A P S E L K A T D V T V D S S V 000001788

>>>>>>>>> | | | | | | | | | | | | | | | | | | | | >>>>>>>>>

115892983 acagacattgacgctccatcagagctgaaagcgacagacgtgacagtcgattcttctgtt 115893042

000001789 L T W V P P L A D I D G Y I L T Y R L E 000001848

>>>>>>>>> | | | | | | | | | | | | | | | | | | | | >>>>>>>>>

115893043 cttacctgggttcctcctctcgccgacatcgatggatacatcctcacctacagacttgag 115893102

000001849 D G N M K 000001863

>>>>>>>>> | | | | | >>>>>>>>>

115893103 gacggcaacatgaag 115893117

000001864 A V E K Q L G R S E S S F S A S G L E T 000001923

>>>>>>>>> | | | | | | | | | | | | | | | | | | | | >>>>>>>>>

115893582 gctgttgagaagcagcttggacgcagcgagagcagtttttcagcgtctggtctggagacg 115893641

000001924 G Q R Y A V T I I A Y R G D K R S K V K 000001983

>>>>>>>>> | | | | | | | | | | | | | | | | | | | | >>>>>>>>>

115893642 ggccagcgatacgctgtcaccatcattgcctacagaggggacaagaggagcaaagtgaag 115893701

000001984 Q A V F K T 000002001

>>>>>>>>> | | | | | | >>>>>>>>>

115893702 caggccgtcttcaaaaca 115893719

000002026 G L S S D H E E R E Q K E W H F H 000002076

>>>>>>>>> | | | | | | | | | | | | | | | | | >>>>>>>>>

115893827 ggactgtcttcagatcatgaagaacgggaacaaaaagagtggcattttcat 115893877

000002077 F I N N D R S K P V E A Y C D M E T D S 000002136

>>>>>>>>> | | | | | | | | | | | | | | | | | | | | >>>>>>>>>

115893882 tttattaacaatgaccgctccaagcctgtggaggcctactgcgatatggagacagacagc 115893941

000002137 G G W L 000002148

>>>>>>>>> | | | | >>>>>>>>>

115893942 gggggctggctg 115893953

000002149 V L Q R R T S G K L D F L K R W R Q Y I 000002208

>>>>>>>>> | | | | | | | | | | | | | | | | | | | | >>>>>>>>>

115894132 gtcctccaaagacgtacaagtggtaagctggactttctgaagcgctggagacagtacatc 115894191

000002209 A G F G N M T D E F W M 000002244

>>>>>>>>> | | | | | | | | | | | | >>>>>>>>>

115894192 gcagggtttggcaacatgacggacgagttctggatg 115894227

000002245 G L D K I Y E L T N T P T R Y E L R F D 000002304

>>>>>>>>> | | | | | | | | | | | | | | | | | | | | >>>>>>>>>

115894334 ggtctggacaagatatatgagctcaccaacactcccacccgctatgagctgaggttcgat 115894393

000002305 L G L G P D R A Y A V Y D N F Q I A S V 000002364

>>>>>>>>> | | | | | | | | | | | | | | | | | | | | >>>>>>>>>

115894394 ctgggcctggggccagacagggcctacgccgtttacgataacttccagatcgcgtcagtc 115894453

000002365 R Q K F K L T I G K Y S G T A 000002409

>>>>>>>>> | | | | | | | | | | | | | | | >>>>>>>>>

115894454 agacagaagttcaaactcaccattgggaaatacagcggcacagca 115894498

000002410 G D A M T Y H R G Q S W T T I D S D N D 000002469

>>>>>>>>> | | | | | | | Q | | | | | | | | | | | | >>>>>>>>>

115895068 ggtgacgctatgacctaccaccaaggccagtcctggaccaccattgactctgacaatgac 115895127

000002470 I A L S N C A L S H R G A W W Y K N C H 000002529

>>>>>>>>> | | | | | | | | | | | | | | | | | | | | >>>>>>>>>

115895128 atcgccctcagtaactgcgctctgagccaccgcggcgcatggtggtacaagaactgccac 115895187

000002530 L A N L N G N W G D N R H S M 000002574

>>>>>>>>> | | | | | | | | | | | | | | | >>>>>>>>>

115895188 ctggcgaacctcaacggaaactggggagacaacaggcacagcatg 115895232

000002575 G V N W K P W K G H L L S L D Y T E M K 000002634

>>>>>>>>> | | | | | | | | | | | | | | | | | | | | >>>>>>>>>

115896271 ggtgtcaactggaaaccgtggaagggtcacctcttgtcgctcgactacaccgagatgaaa 115896330

000002635 I R P A G A L S G R K R R S L 000002679

>>>>>>>>> | | | | | | | | | | | | | | | >>>>>>>>>

115896331 atccgacccgcgggggccttgtccggcaggaagaggcggtcgtta 115896375

Takifugu tenascin-R (partial):

000000001 V H T T R V R R Q T P A G G Q A P P A S 000000060

<<<<<<<<< | | | | | | | | | | | | | | | | | | | | <<<<<<<<<

115951944 gttcataccaccagagtgaggagacagaccccggcggggggtcaggctcctcccgcttct 115951885

000000061 S E N Q T A R E Q P L V F N H V Y N I N 000000120

<<<<<<<<< | | | | | | | | | | | | | | | | | | | | <<<<<<<<<

115951884 tcagaaaaccagaccgcaagggaacaacctctggtgtttaatcacgtgtacaacatcaat 115951825

000000121 V P L E S L C S V E L D S A A S P G P A 000000180

<<<<<<<<< | | | | | | | | | | | | | | | | | | | | <<<<<<<<<

115951824 gtacctctggagtccctctgttctgtcgaactggactctgctgcatcacccggtccagcc 115951765

000000181 N 000000183

<<<<<<<<< | <<<<<<<<<

115951764 aat 115951762

000000184 V D P S G P S E Y T E Q T M D P D S Q 000000240

<<<<<<<<< | | | | | | | | | | | | | | | | | | | <<<<<<<<<

115951630 gtggatccttcgggcccatcagaatacacagagcagacaatggaccccgacagccag 115951574

000000241 V T F T H R I N I P K A A C G C P A T A 000000300

<<<<<<<<< | | | | | | | | | | | | | | | | | | | | <<<<<<<<<

115951501 gtaacgttcacccaccgcatcaacatcccaaaagctgcatgcggttgtccggcaacagcc 115951442

000000301 T I Q Q L V T R V E M L E R E V S L L R 000000360

<<<<<<<<< | | | | | | | | | | | | | | | | | | | | <<<<<<<<<

115951441 acgatacagcagctggtcaccagggtggaaatgctggagagagaggtctccctgctcaga 115951382

000000361 A Q C G S G C C G E G S A M 000000402

<<<<<<<<< | | | | | | | | | | | | | | <<<<<<<<<

115951381 gcccagtgtggctcggggtgctgcggcgagggctccgccatg 115951340

000000403 G R L D F V P G C S G R G S F S F D L C 000000462

<<<<<<<<< | | | | | | | | | | | | | | | | | | | | <<<<<<<<<

115950843 ggtcgtttggactttgtcccgggctgcagcgggcgcggcagcttcagctttgacctctgc 115950784

000000463 G C I C E E G W A G K N C S E P R C P D 000000522

<<<<<<<<< | | | | | | | | | | | | | | | | | | | | <<<<<<<<<

115950783 gggtgtatctgtgaggaaggctgggcgggtaaaaactgctctgagccccgctgtccagat 115950724

000000523 D C S G Q G A C V E G E C V C D R D F S 000000582

<<<<<<<<< | | | | | | | | | | | | | | | | | | | | <<<<<<<<<

115950723 gactgctcgggtcagggggcctgcgtggagggcgagtgtgtgtgtgaccgtgatttcagc 115950664

000000583 G E N C S E P R C P S D C S G R G L C I 000000642

<<<<<<<<< | | | | | | | | | | | | | | | | | | | | <<<<<<<<<

115950663 ggagagaactgctccgagccacggtgcccctccgactgctcgggccgagggctgtgcatc 115950604

000000643 D G E C V C E E S Y T G E D C M V G R C 000000702

<<<<<<<<< | | | | | | | | | | | | | | | | | | | | <<<<<<<<<

115950603 gacggcgagtgcgtgtgcgaggagtcctacaccggagaggactgcatggtcgggaggtgt 115950544

000000703 L N D C S D Q G A C V N G T C Q C R P G 000000762

<<<<<<<<< | | | | | | | | | | | | | | | | | | | | <<<<<<<<<

115950543 ctgaacgactgctcggaccagggcgcctgcgtcaatgggacatgccagtgccggcccggg 115950484

000000763 Y I G E D C S L V Y C A N N C S K K G V 000000822

<<<<<<<<< | | | | | | | | | | | | | | | | | | | | <<<<<<<<<

115950483 tacatcggggaagactgctcgctggtgtactgtgccaacaactgcagcaagaagggggtg 115950424

000000823 C K T G F C V C Q D G F A G D D C N S 000000879

<<<<<<<<< | | | | | | | | | | | | | | | | | | | <<<<<<<<<

115950423 tgcaagaccgggttctgtgtctgccaggacggctttgctggagatgactgcaactca 115950367

000000880 V A P V M N L K I R G G T D R S I D L E 000000939

<<<<<<<<< | | | | | | | | | | | | | | | | | | | | <<<<<<<<<

115942468 gtggcgccggtcatgaatctgaagatccgtggtgggaccgatcgcagcattgacctggag 115942409

000000940 W E G S V V L T D F L V T Y T P S S T G 000000999

<<<<<<<<< | | | | | | | | | | | | | | | | | | | | <<<<<<<<<

115942408 tgggagggctcggtggtgctgacagattttttggtgacgtacacgccaagcagcactgga 115942349

000001000 G I P L E I R I P G N T T A C T I S G L 000001059

<<<<<<<<< | | | | | | | | | | | | | | | | | | | | <<<<<<<<<

115942261 ggtatcccgttagagattaggattccaggcaataccaccgcctgcaccatctcgggcctg 115942202

000001060 E A G V E Y N I N V F A V I N N S I S V 000001119

<<<<<<<<< | | | | | | | | | | | | | | | | | | | | <<<<<<<<<

115942201 gaggcaggcgtggaatacaacatcaacgtcttcgctgtcatcaacaacagcatcagcgtc 115942142

000001120 P A G I A V S T C K 000001149

<<<<<<<<< | | | | | | | | | | <<<<<<<<<

115942141 cctgctggcattgccgtctcgacatgcaag 115942112

000001153 L S N P D G L V F K S I T E T S V E V Q 000001212

<<<<<<<<< | | | | | | | | | | | | | | | | | | | | <<<<<<<<<

115941331 ctctccaatcctgatggtttagtcttcaaatccatcacagaaacatcggtggaggtccag 115941272

000001213 W K P F Y Y S F D G W E I S F I P K 000001266

<<<<<<<<< | | | | | | | | | | | | | | | | | | <<<<<<<<<

115941271 tggaaaccattctactattcctttgatggctgggagatcagcttcatcccaaag 115941218

000001267 D N D G G M T A Q L P S T I T S F V Q T 000001326

<<<<<<<<< | | | | | | | | | | | | | | | | | | | | <<<<<<<<<

115937946 gacaatgacggagggatgacagctcagctgccgagcaccatcacttcatttgtgcagact 115937887

000001327 G L R P G E E Y T V N L V A L R D Q G R 000001386

<<<<<<<<< | | | | | | | | | | | | | | | | | | | | <<<<<<<<<

115937886 ggactgagaccaggggaggaatacaccgtgaacctggtggccctccgagaccagggtaga 115937827

000001387 S Q P V T A T V I T 000001416

<<<<<<<<< | | | | | | | | | | <<<<<<<<<

115937826 agccagcctgtcacagccaccgtcatcacc 115937797

000001420 I D G P T Q L I V R D I S D T V A F V E 000001479

<<<<<<<<< | | | | | | | | | | | | | | | | | | | | <<<<<<<<<

115935607 atcgatggacccacacagctgattgtgcgggatatatctgacaccgtagcttttgttgag 115935548

000001480 W T P P K A K L D Q I V L R Y G L V G E 000001539

<<<<<<<<< | | | | | | | | | | | | | | | | | | | | <<<<<<<<<

115935547 tggaccccaccgaaagcaaagcttgatcaaattgtgttgcgctatggtttggtgggagaa 115935488

000001540 G P R T T F R L Q P T L S Q Y S 000001587

<<<<<<<<< | | | | | | | | | | | | | | | | <<<<<<<<<

115935487 ggtcctaggaccaccttccggctccagcctacactcagccagtactcc 115935440

000001588 L Q V L R P G S R Y E V S V T G V R T G 000001647

<<<<<<<<< | | | | | | | | | | | | | | | | | | | | <<<<<<<<<

115934766 ctgcaggttctgcgccctggttctcgttacgaagtgtctgtgacaggtgtgaggacagga 115934707

000001648 N E S G S I S T E F T T 000001683

<<<<<<<<< | | | | | | | | | | | | <<<<<<<<<

115934706 aacgagagcggatctatttccactgaattcacgacc 115934671

000001684 E I D A P K N L R L V S K T S T A L E L 000001743

<<<<<<<<< | | | | | | | | | | | | | | | | | | | | <<<<<<<<<

115934447 gaaatcgatgcccccaagaacctgcggctcgtgtcaaagacctccaccgccctggagctg 115934388

000001744 E W D N S E A E 000001767

<<<<<<<<< | | | | | | | | <<<<<<<<<

115934387 gaatgggataacagcgaggctgag 115934364

000001768 V E G Y Q V V Y S T L A G E Q Y D K V I 000001827

<<<<<<<<< | | | | | | | | | | | | | | | | | | | | <<<<<<<<<

115933885 gtggagggctaccaggtggtgtacagcactttagcaggagaacagtacgataaagttata 115933826

000001828 V P R N E G A T T R T T L T 000001869

<<<<<<<<< | | | | | | | | | | | | | | <<<<<<<<<

115933825 gttccccgcaacgagggagccaccacgaggaccaccctgacc 115933784

000001870 D L L P G T E Y G I G I S A M K G S N Q 000001929

<<<<<<<<< | | | | | | | | | | | | | | | | | | | | <<<<<<<<<

115933150 gacctgctgccgggcactgagtacggcattggcatttctgctatgaaaggcagcaaccag 115933091

000001930 S T P A T M N A R T 000001959

<<<<<<<<< | | | | | | | | | | <<<<<<<<<

115933090 agcacaccagcaacaatgaacgccaggaca 115933061

000001960 G L D V P M D L T V T A S T D K T I T L 000002019

<<<<<<<<< | | | | | | | | | | | | | | | | | | | | <<<<<<<<<

115932591 gggttggacgtgcccatggatctcaccgtgacagcttctacagacaaaaccatcacactc 115932532

000002020 V W G V V Q G P I D H Y K V T C T S S S 000002079

<<<<<<<<< | | | | | | | | | | | | | | | | | | | | <<<<<<<<<

115932531 gtgtggggtgtggtccagggtcccatcgaccattataaggtcacctgcacatcctcttct 115932472

000002080 G V T T E 000002094

<<<<<<<<< | | | | | <<<<<<<<<

115932471 ggagtcaccactgag 115932457

000002095 L T V P K D I T T I T L A E L D P G T E 000002154

<<<<<<<<< | | | | | | | | | | | | | | | | | | | | <<<<<<<<<

115932363 ctgacagtgcccaaagatattaccaccatcaccctggcagagctggaccccgggactgag 115932304

000002155 Y T V T V A A R R G R Q Q S N V A T I D 000002214

<<<<<<<<< | | | | | | | | | | | | | | | | | | | | <<<<<<<<<

115932303 tacaccgtcacggtggcagcaagaagaggacggcaacaaagcaatgtcgccacaatcgac 115932244

000002215 A F T 000002223

<<<<<<<<< | | | <<<<<<<<<

115932243 gcctttaca 115932235

000002224 G I R P V T H L F L S E V T S D S V L V 000002283

<<<<<<<<< | | | | | | | | | | | | | | | | | | | | <<<<<<<<<

115930093 ggaatcaggcccgtaactcacctcttcttatcagaagtcacgtcagactctgtgttggtg 115930034

000002284 A W S A P A P P A D L F I L S Y S S S D 000002343

<<<<<<<<< | | | | | | | | | | | | | | | | | | | | <<<<<<<<<

115930033 gcctggagcgccccggcaccacctgccgacctcttcatcctgagctacagctcttccgac 115929974

000002344 G T D S S K V T L D G S K T A S L V Q G 000002403

<<<<<<<<< | | | | | | | | | | | | | | | | | | | | <<<<<<<<<

115929973 gggacagactcatctaaggtgacgctggatggctctaagacagcatctctggtccagggg 115929914

000002404 L L P S T P Y T I S L I T I Q A D V T S 000002463

<<<<<<<<< | | | | | | | | | | | | | | | | | | | | <<<<<<<<<

115929913 ctgttgccgtccacaccgtacaccatcagtctaatcacaatacaagcggacgtcacctct 115929854

000002464 D P I T A S L T T 000002490

<<<<<<<<< | | | | | | | | | <<<<<<<<<

115929853 gaccccattacagcatcgctcactaca 115929827

000002491 G L D P P K E M M V S D V T E D S V T L 000002550

<<<<<<<<< | | | | | | | | | | | | | | | | | | | | <<<<<<<<<

115928263 ggcctggacccgccaaaagagatgatggtctcagatgtgaccgaggactctgtcactctc 115928204

000002551 S W I R P L A P F E Y Y K L S Y Q S A R 000002610

<<<<<<<<< | | | | | | | | | | | | | | | | | | | | <<<<<<<<<

115928203 tcttggatcagaccgttggctccatttgaatactataagctgtcctaccagtcagccaga 115928144

000002611 G R V D S M V I D S D V T N Y T L S S L 000002670

<<<<<<<<< | | | | | | | | | | | | | | | | | | | | <<<<<<<<<

115927800 ggccgagtggacagcatggtgattgacagcgatgtgaccaactacaccttgtccagcctg 115927741

000002671 F P A T E Y E I S I S A V K E S Q E S D 000002730

<<<<<<<<< | | | | | | | | | | | | | | | | | | | | <<<<<<<<<

115927740 ttccctgctacggaatatgaaatcagcatcagtgctgtcaaagagagccaggagagcgac 115927681

000002731 V V R T S V F T 000002754

<<<<<<<<< | | | | | | | | <<<<<<<<<

115927680 gtggttaggacctctgtcttcaca 115927657

000002755 A M D M P S E L T A L N I T P R G A L L 000002814

<<<<<<<<< | | | | | | | | | | | | | | | | | | | | <<<<<<<<<

115925398 gcgatggacatgccctctgagctgacggctctgaatatcaccccacggggagccttgctg 115925339

000002815 R W N P P L S V V D N Y V L T L T H N 000002871

<<<<<<<<< | | | | | | | | | | | | | | | | | | | <<<<<<<<<

115925338 aggtggaacccccctctgtctgtggtcgacaattatgtgctgaccctcacacacaat 115925282

000002872 Q V T A D T F L V E G I K Q E H Q L S N 000002931

<<<<<<<<< | | | | | | | | | | | | | | | | | | | | <<<<<<<<<

115924752 caagtgacagctgacacattcctggtggaaggcatcaagcaggagcaccaactgtccaac 115924693

000002932 L S P S T S Y S V A L Y A T K G P L T S 000002991

<<<<<<<<< | | | | | | | | | | | | | | | | | | | | <<<<<<<<<

115924692 ctaagtcccagcaccagctactctgtggctctgtacgctaccaaagggccgttgaccagc 115924633

000002992 G T V I T N L Q T 000003018

<<<<<<<<< | | | | | | | | | <<<<<<<<<

115924632 ggcactgtgatcaccaacctgcaaaca 115924606

000003022 M D A P L N L T A S E V N H R S A L I S 000003081

<<<<<<<<< | | | | | | | | | | | | | | | | | | | | <<<<<<<<<

115920677 atggatgcgcctttgaacctgacggccagcgaggtgaatcaccgcagcgccctcatctcc 115920618

000003082 W Q P P M A E I D N Y M L T Y K S A D S 000003141

<<<<<<<<< | | | | | | | | | | | | | | | | | | | | <<<<<<<<<

115920617 tggcaaccaccgatggcagagatcgataactacatgctcacttacaagtcagccgacagc 115920558

000003142 G R K 000003150

<<<<<<<<< | | | <<<<<<<<<

115920557 ggccgtaaa 115920549

000003151 E L I L D A E D T W I R L E G L A E I T 000003210

<<<<<<<<< | | | | | | | | | | | | | | | | | | | | <<<<<<<<<

115918360 gagctgatactagatgctgaggacacgtggatacgactggaggggctggctgagatcaca 115918301

000003211 E Y T V N L Q A A R G L D T S A V V S T 000003270

<<<<<<<<< | | | | | | | | | | | | | | | | | | | | <<<<<<<<<

115918300 gagtacaccgttaatctccaggcggccagaggtctcgacacaagtgctgtcgtctccact 115918241

000003271 T F I T 000003282

<<<<<<<<< | | | | <<<<<<<<<

115918240 acctttattaca 115918229

000003283 G S R L F A T P Q N C A Q H L L N G E T 000003342

<<<<<<<<< | | | | | | | | | | | | | | | | | | | | <<<<<<<<<

115917195 gggagtcgtctctttgccacgcctcagaactgcgcccagcacctcctgaatggcgagacg 115917136

000003343 L S G V Y T I Y I N R D P S Q G V Q V Y 000003402

<<<<<<<<< | | | | | | | | | | | | | | | | | | | | <<<<<<<<<

115917135 ctgagcggcgtttataccatctacattaaccgtgaccccagccagggtgtgcaggtgtac 115917076

000003403 C D M T T D E G G W I 000003435

<<<<<<<<< | | | | | | | | | | | <<<<<<<<<

115917075 tgcgacatgaccacggacgagggaggctggatt 115917043

000003436 V F Q R R Q N G L T D F S R K W S D Y R 000003495

<<<<<<<<< | | | | | | | | | | | | | | | | | | | | <<<<<<<<<

115916971 gtgttccagcggcgccagaacggcctgactgatttttccaggaagtggagcgactatcgc 115916912

000003496 V G F G N L E D E F W L 000003531

<<<<<<<<< | | | | | | | | | | | | <<<<<<<<<

115916911 gtcggctttggaaacctggaggatgaattctggctt 115916876

000003532 G L D N I Q R I A A Q G R Y E L R I D M 000003591

<<<<<<<<< | | | | | | | | | | | | | | | | | | | | <<<<<<<<<

115914221 ggcttagacaacatccagaggattgcggctcagggtcgttacgagctgcgaatcgacatg 115914162

000003592 K D G Q E S V Y A N Y D K F S I G D A R 000003651

<<<<<<<<< | | | | | | | | | | | | | | | | | | | | <<<<<<<<<

115914161 aaggatggacaggagtctgtgtatgccaattatgataaattctccattggtgatgcacga 115914102

000003652 N L Y K L R I G E Y N G T A 000003693

<<<<<<<<< | | | | | | | | | | | | | | <<<<<<<<<

115914101 aacctctacaagctcaggataggagagtacaatggaactgcc 115914060

000003694 G D S L S Y H Q G R P F S T K D R D N D 000003753

<<<<<<<<< | | | | | | | | | | | | | | | | | | | | <<<<<<<<<

115913731 ggtgactctctgagctatcaccagggtcgtcctttctcgacaaaagacagggacaatgac 115913672

000003754 I A V T N C A L S Y K G A W W Y K N C H 000003813

<<<<<<<<< | | | | | | | | | | | | | | | | | | | | <<<<<<<<<

115913671 atcgctgtcactaactgcgccttgtcttacaaaggagcctggtggtataagaactgccac 115913612

000003814 R A N L N G K Y G E S R H S 000003855

<<<<<<<<< | | | | | | | | | | | | | | <<<<<<<<<

115913611 cgggctaaccttaatggcaaatatggcgagtcaagacacagc 115913570

000003856 Q G I N W Y H W K G H E F S I P F V E M 000003915

<<<<<<<<< | | | | | | | | | | | | | | | | | | | | <<<<<<<<<

115912593 cagggtattaactggtaccactggaaaggccacgagttctccattccctttgtggagatg 115912534

000003916 K M R P F N Y R S I S S K R R R S 000003966

<<<<<<<<< | | | | | | | | | | | | | | | | | <<<<<<<<<

115912533 aagatgagacctttcaactaccgtagcatcagcagcaagcggaggcggtcc 115912483

Takifugu tenascin-X:

000000001 M T H K I S L L P G G C S G G C E T E M 000000060

>>>>>>>>> | | | | | | | | | | | | | | | | | | | | >>>>>>>>>

174788619 atgacccataagatcagcttgttgcccggtggctgttcaggggggtgtgagactgagatg 174788678

000000061 S A L K E R V A R L E K E M S S L K D N 000000120

>>>>>>>>> | | | | | | | | | | | | | | | | | | | | >>>>>>>>>

174788679 agtgccctgaaggagcgtgtggcccgcttggaaaaagagatgtcctccctaaaggataac 174788738

000000121 C 000000123

>>>>>>>>> | >>>>>>>>>

174788739 tgt 174788741

000000124 P C S V N C P N D C S G N G E C Q K G K 000000183

>>>>>>>>> | | | | | | | | | | | | | | | | | | | | >>>>>>>>>

174788815 ccgtgttctgtcaactgtccaaatgactgtagtggcaatggggaatgccagaaggggaaa 174788874

000000184 C V C Q Q G F M G P D C S N C A Q G A E 000000243

>>>>>>>>> | | | | | | | | | | | | | | | | | | | | >>>>>>>>>

174788875 tgtgtctgccagcaggggttcatggggccagactgcagcaattgtgcccaaggagctgag 174788934

000000244 C I K S K W G E K S S K F T K E S L T G 000000303

>>>>>>>>> | | | | | | | | | | | | | | | | | | | | >>>>>>>>>

174788935 tgcatcaaaagtaagtggggggaaaaatcatctaaatttactaaagagtccttgacagga 174788994

000000304 T C I N A V S E A V K G K A K S T G K T 000000363

>>>>>>>>> | | | | | | | | | | | | | | | | | | | | >>>>>>>>>

174788995 acatgtattaatgctgtttcagaagctgtcaaaggaaaggccaaatcaacagggaagaca 174789054

000000364 V T V Q G E K D K S S A Q T K G E N T L 000000423

>>>>>>>>> | | | | | | | | | | | | | | | | | | | | >>>>>>>>>

174789055 gtgacagtgcaaggagaaaaagacaaaagcagtgcccagaccaaaggagagaacactctc 174789114

000000424 S Q D K E E K K V T E G K N T L S Q G K 000000483

>>>>>>>>> | | | | | | | | | | | | | | | | | | | | >>>>>>>>>

174789115 tcacaggacaaggaggagaagaaggttacggaaggaaagaacactctctcacagggcaag 174789174

000000484 E E K V T E G K N T L S R G K E E K K V 000000543

>>>>>>>>> | | | | | | | | | | | | | | | | | | | | >>>>>>>>>

174789175 gaggagaaggttacggaaggaaagaacactctctcacggggcaaggaggaaaagaaggtt 174789234

000000544 T E G K N T L S Q G K E E K K V T E G K 000000603

>>>>>>>>> | | | | | | | | | | | | | | | | | | | | >>>>>>>>>

174789235 acggaaggaaagaacactctctcgcagggcaaggaggagaagaaggttacggaaggaaag 174789294

000000604 N T L S Q G K E E K K V T E G K N T L S 000000663

>>>>>>>>> | | | | | | | | | | | | | | | | | | | | >>>>>>>>>

174789295 aacactctctcgcaaggaaaggaggagaagaaggttacggaaggaaagaacactctctcg 174789354

000000664 Q G K G E K T V T E G K N T L S Q G K G 000000723

>>>>>>>>> | | | | | | | | | | | | | | | | | | | | >>>>>>>>>

174789355 cagggcaagggggagaaaacagttacggaaggaaagaacactctctcgcaaggaaagggg 174789414

000000724 E T K V T E G K N T L S Q G K E E K K V 000000783

>>>>>>>>> | | | | | | | | | | | | | | | | | | | | >>>>>>>>>

174789415 gagacgaaggttacggaaggaaagaacactctctcgcaaggaaaggaggagaagaaggtt 174789474

000000784 T E G V N T L S Q G K E E K K V T E G K 000000843

>>>>>>>>> | | | | | | | | | | | | | | | | | | | | >>>>>>>>>

174789475 acggaaggagtgaacactctctcacagggcaaggaggaaaagaaggttacggaaggaaag 174789534

000000844 N T L S Q G K E E K K V T E G K N T L S 000000903

>>>>>>>>> | | | | | | | | | | | | | | | | | | | | >>>>>>>>>

174789535 aacactctctcgcaaggaaaggaggagaagaaggttacggaaggaaagaacactctctcg 174789594

000000904 Q G K E E K K V T E G K N T L S Q G K E 000000963

>>>>>>>>> | | | | | | | | | | | | | | | | | | | | >>>>>>>>>

174789595 cagggcaaggaggagaaaaaagttacggaaggaaagaacactctctcgcaaggaaaggag 174789654

000000964 E K K V T E G K N T L L Q G K E E K K V 000001023

>>>>>>>>> | | | | | | | | | | | | | | | | | | | | >>>>>>>>>

174789655 gagaagaaggttacggaaggaaagaacactctcttgcaaggaaaggaggagaagaaggtt 174789714

000001024 T E G E N T L S Q G K E E K K 000001068

>>>>>>>>> | | | | | | | | | | | | | | | >>>>>>>>>

174789715 acagaaggagagaacactctctcgcaaggaaaggaggagaagaag 174789759

000001069 G K E E K T V M E G K N T L S Q G K E E 000001128

>>>>>>>>> | | | | | | | | | | | | | | | | | | | | >>>>>>>>>

174789792 ggcaaggaggagaaaacagttatggaaggaaagaacactctctcgcaaggaaaggaggag 174789851

000001129 K K V T E G K N T L S Q G K E E K K V M 000001188

>>>>>>>>> | | | | | | | | | | | | | | | | | | | | >>>>>>>>>

174789852 aagaaggttacggaaggaaagaacactctctcacagggcaaggaggaaaagaaggttatg 174789911

000001189 E G K N T I S Q G K E E K K V M E G E S 000001248

>>>>>>>>> | | | | | | | | | | | | | | | | | | | | >>>>>>>>>

174789912 gaaggaaagaacactatctcccagggaaaggaggagaagaaggttatggaaggagagagc 174789971

000001249 T L L Q G K E Q K K V T E I K D A D A K 000001308

>>>>>>>>> | | | | | | | | | | | | | | | | | | | | >>>>>>>>>

174789972 actcttttgcagggaaaggaacagaagaaggttactgaaatcaaagatgctgatgctaaa 174790031

000001309 P K V T T G T F T K L S L S K T Q T K Q 000001368

>>>>>>>>> | | | | | | | | | | | | | | | | | | | | >>>>>>>>>

174790032 cccaaagtgactaccggaactttcactaaactgagtctcagtaaaacacaaacaaagcaa 174790091

000001369 E V S T K K T P T K D S G A A K T T H P 000001428

>>>>>>>>> | | | | | | | | | | | | | | | | | | | | >>>>>>>>>

174790092 gaagtttcgacaaagaaaacacctactaaagactctggagcagcaaagaccactcaccca 174790151

000001429 T V S Q V Q V K H D K G N Q E E S R K E 000001488

>>>>>>>>> | | | | | | | | | | | | | | | | | | | | >>>>>>>>>

174790152 actgtttctcaggttcaagtgaagcatgataaaggaaaccaagaggaatcccgcaaagaa 174790211

000001489 K T V T A S K K V Q K P E V K L G T T S 000001548

>>>>>>>>> | | | | | | | | | | | | | | | | | | | | >>>>>>>>>

174790212 aaaacagtgacagcttctaaaaaggtacaaaaacctgaagtaaaacttggaactaccagc 174790271

000001549 K T Y S N S E Q L K D E P Q T N R T Q S 000001608

>>>>>>>>> | | | | | | | | | | | | | | | | | | | | >>>>>>>>>

174790272 aaaacatattctaattctgaacagttaaaagatgaacctcaaacaaacagaacccagagt 174790331

000001609 N V K T S T S S S K T L I T L S K S V G 000001668

>>>>>>>>> | | | | | | | | | | | | | | | | | | | | >>>>>>>>>

174790332 aatgtaaagacatcaactagttcctcaaaaaccttgataactctgtccaaaagtgttgga 174790391

000001669 T K K N K V E K E S S E H S T L P E K S 000001728

>>>>>>>>> | | | | | | | | | | | | | | | | | | | | >>>>>>>>>

174790392 actaaaaaaaacaaagtagagaaggagtcttcggagcactctactctacctgagaagagt 174790451

000001729 V V Q S V G Q K N I K K V K G D A M P S 000001788

>>>>>>>>> | | | | | | | | | | | | | | | | | | | | >>>>>>>>>

174790452 gttgttcaatcagttggccagaaaaacatcaagaaggtcaaaggagatgcaatgccttcg 174790511

000001789 Q S T D N K S T E V I K I V K D T E D K 000001848

>>>>>>>>> | | | | | | | | | | | | | | | | | | | | >>>>>>>>>

174790512 caatccactgacaacaaaagcactgaagttatcaaaattgtaaaagacacagaagacaaa 174790571

000001849 V L Q R S Q D L V N G T G V S I L S E G 000001908

>>>>>>>>> | | | | | | | | | | | | | | | | | | | | >>>>>>>>>

174790572 gttttacagaggagtcaggatttagttaatggtacaggggtgtcaatattgagtgaggga 174790631

000001909 Q D T R D K T T T Q G S G S V R V L G G 000001968

>>>>>>>>> | | | | | | | | | | | | | | | | | | | | >>>>>>>>>

174790632 caggacacgcgggacaaaacaacaacgcaaggctctggcagtgtgagggttctgggagga 174790691

000001969 S G L G S V K V A N V S S Y S F T L M W 000002028

>>>>>>>>> | | | | | | | | | | | | | | | | | | | | >>>>>>>>>

174790692 tctggattaggttcggtgaaggttgccaatgtctcctcctacagcttcactctcatgtgg 174790751

000002029 S A P Q G M F K N F T L I R T E S L T E 000002088

>>>>>>>>> | | | | | | | | | | | | | | | | | | | | >>>>>>>>>

174790752 tcagcaccacaaggaatgttcaaaaacttcacattgatcaggacagaatcgttgacagaa 174790811

000002089 G T Q V D H E E F E E E T F E E V K T Y 000002148

>>>>>>>>> | | | | | | | | | | | | | | | | | | | | >>>>>>>>>

174790812 ggtacccaggttgatcatgaggagtttgaggaggagacttttgaagaagtcaagacttac 174790871

000002149 T I K N T T A R V Q V P T E S S N T T V 000002208

>>>>>>>>> | | | | | | | | | | | | | | | | | | | | >>>>>>>>>

174790872 acgataaagaacacaacggctagagtccaggtaccgactgaaagcagcaacaccactgtg 174790931

000002209 V S E S R G K A E T R R I S M V I P G N 000002268

>>>>>>>>> | | | | | | | | | | | | | | | | | | | | >>>>>>>>>

174790932 gtgtctgagtcaagaggcaaagctgaaaccagaagaatctccatggtgatccctggaaac 174790991

000002269 V R S V E F S N L R P N T A Y V L Q I Y 000002328

>>>>>>>>> | | | | | | | | | | | | | | | | | | | | >>>>>>>>>

174790992 gtgcgctctgtggagttcagcaaccttcggccaaacacagcgtatgtccttcaaatctac 174791051

000002329 G S A A Q R R S K I H R V T T V T 000002379

>>>>>>>>> | | | | | | | | | | | | | | | | | >>>>>>>>>

174791052 ggcagtgcagcacagaggaggtcaaagatccacagagtaaccacagtcaca 174791102

000002380 G P E P A T E M V F S N V T E S S L S V 000002439

>>>>>>>>> | | | | | | | | | | | | | | | | | | | | >>>>>>>>>

174791174 ggtccagaaccagccacagagatggttttcagtaatgtgacagagtcctccctcagtgtt 174791233

000002440 S W S K P K T T Y A A F R I T Y T N I V 000002499

>>>>>>>>> | | | | | | | | | | | | | | | | | | | | >>>>>>>>>

174791234 tcctggtccaaaccaaagacaacgtacgcagccttccggatcacatacaccaacattgtc 174791293

000002500 T 000002502

>>>>>>>>> | >>>>>>>>>

174791294 aca 174791296

000002503 G E Y H Y V T V S S Q Q S H V V L T K L 000002562

>>>>>>>>> | | | | | | | | | | | | | | | | | | | | >>>>>>>>>

174791395 ggagagtaccattatgtgaccgtgagctctcagcaatctcatgtggttctcaccaagtta 174791454

000002563 S V G T S Y I V S V M A T Q G R T Q S D 000002622

>>>>>>>>> | | | | | | | | | | | | | | | | | | | | >>>>>>>>>

174791455 tctgttggaacctcctacatagtttctgtcatggctacacaaggcagaacccagagtgat 174791514

000002623 A L T S I I T T 000002646

>>>>>>>>> | | | | | | | | >>>>>>>>>

174791515 gctctaacatccatcataaccaca 174791538

000002647 V P A P P T H L R V V N V T D T K A L L 000002706

>>>>>>>>> | | | | | | | | | | | | | | | | | | | | >>>>>>>>>

174791655 gtacccgcccctccaacacatcttagagtcgtcaacgtgacagataccaaagctctgctg 174791714

000002707 Q W T P S L G K V D R F I I S Y E S S K 000002766

>>>>>>>>> | | | | | | | | | | | | | | | | | | | | >>>>>>>>>

174791715 caatggacccccagtttgggaaaagtagatcgcttcatcatcagctatgagtcctccaag 174791774

000002770 P N V T V T V M L S G N S V E H Q L R G 000002829

>>>>>>>>> | | | | | | | | | | | | | | | | | | | | >>>>>>>>>

174791858 cctaatgtgaccgtgactgtgatgttgtctgggaactcggtggaacaccagctgagaggc 174791917

000002830 L Q R G T M Y T V K V L S Q K D S H Q S 000002889

>>>>>>>>> | | | | | | | | | | | | | | | | | | | | >>>>>>>>>

174791918 ctgcagagaggcaccatgtacacagttaaagtcctgagtcagaaggacagtcaccagagc 174791977

000002890 M A V S T T F T T A N 000002922

>>>>>>>>> | | | | | | | | | | | >>>>>>>>>

174791978 atggccgtctcaaccacatttaccactgctaat 174792010

000002926 V K A S E V S A R Y A V I I W R T S T V 000002985

>>>>>>>>> | | | | | | | | | | | | | | | | | | | | >>>>>>>>>

174792086 gtcaaagccagtgaggtcagtgcccgctacgcagtgatcatctggagaacctccacagtc 174792145

000002986 V Y H S Y R L I Y Q V A G E E T K 000003036

>>>>>>>>> | | | | | | | | | | | | | | | | | >>>>>>>>>

174792146 gtctaccatagctacaggctgatctaccaggtggctggagaggagacaaag 174792196

000003037 E V I L D G T M T E Y K L 000003075

>>>>>>>>> | | | | | | | | | | | | | >>>>>>>>>

174792273 gaggtgatcttggatggaaccatgacagagtataagctg 174792311

000003154 T G L L P M S R Y I V L V Q G E R D G R 000003213

>>>>>>>>> | | | | | | | | | | | | | | | | | | | | >>>>>>>>>

174792312 acaggcctgctgccaatgtcgcgttacattgttctggttcagggagagagagatggccgc 174792371

000003214 Y T S I V T T E F I T 000003246

>>>>>>>>> | | | | | | | | | | | >>>>>>>>>

174792372 tacacatctatagtcacaacagaattcatcact 174792404

000003247 G K L R F P F P T D C S Q E L L N G A L 000003306

>>>>>>>>> | | | | | | | | | | | | | | | | | | | | >>>>>>>>>

174792488 ggcaaactacggttccccttccctactgattgctcccaggagctactaaacggagctctg 174792547

000003307 E S G E V D I Y P Q G K E G R S V R V Y 000003366

>>>>>>>>> | | | | | | | | | | | | | | | | | | | | >>>>>>>>>

174792548 gagtcaggggaggtggacatctacccccagggaaaagagggacggtcggtccgcgtgtat 174792607

000003367 C D M E T D G G G W T 000003399

>>>>>>>>> | | | | | | | | | | | >>>>>>>>>

174792608 tgtgacatggagaccgatggaggcggctggaca 174792640

000003400 V F Q R R M N G R T D F Y R T W T E Y S 000003459

>>>>>>>>> | | | | | | | | | | | | | | | | | | | | >>>>>>>>>

174792718 gtgttccagaggaggatgaatggaaggacggatttctacagaacctggactgagtacagt 174792777

000003460 A G F G N L S E E F W L 000003495

>>>>>>>>> | | | | | | | | | | | | >>>>>>>>>

174792778 gccggattcggaaacctcagtgaagaattctggctc 174792813

000003496 G N D V L Y N L T S V G P M S L R V D M 000003555

>>>>>>>>> | | | | | | | | | | | | | | | | | | | | >>>>>>>>>

174792891 ggaaatgatgttctctacaacctgaccagcgtcggccccatgagtctgagagtggatatg 174792950

000003556 R Y G N K T V Y A H Y A N F S I D S K E 000003615

>>>>>>>>> | | | | | | | | | | | | | | | | | | | | >>>>>>>>>

174792951 cgatatggaaataaaactgtttatgctcactatgctaacttctccatcgattcaaaggag 174793010

000003616 R H Y T L T V S G Y T G N A 000003657

>>>>>>>>> | | | | | | | | | | | | | | >>>>>>>>>

174793011 aggcactacactctgacagtgtctggttacaccggaaatgca 174793052

000003658 G D S M R Y H N S R P F S S W D K N P D 000003717

>>>>>>>>> | | | | | | | | | | | | | | | | | | | | >>>>>>>>>

174793265 ggtgactccatgaggtaccataacagtcgtccattctcatcttgggacaagaaccctgat 174793324

000003718 P L G I H C A R S Y M G G W W Y K N C Y 000003777

>>>>>>>>> | | | | | | | | | | | | | | | | | | | | >>>>>>>>>

174793325 cctctgggaatccactgtgccaggtcttacatgggaggctggtggtacaagaactgttac 174793384

000003778 K T N L N G 000003795

>>>>>>>>> | | | | | | >>>>>>>>>

174793385 aagaccaacctcaacggt 174793402

Takifugu tenascin-CA:

000000001 M G M K I I L L A C I S M S L L F E L S 000000060

>>>>>>>>> | | | | | | | | | | | | | | | | | | | | >>>>>>>>>

266620049 atgggcatgaaaattatcctcttagcttgtatatccatgagcctgctctttgaactctcc 266620108

000000061 T P G L V R K I I R H R R E A L M P K K 000000120

>>>>>>>>> | | | | | | | | | | | | | | | | | | | | >>>>>>>>>

266620109 actcctggcctggtcagaaaaataatccgccaccgtagagaagctctgatgcctaaaaaa 266620168

000000121 S Q E N I T L P L P D Q P V V F N H V Y 000000180

>>>>>>>>> | | | | | | | | | | | | | | | | | | | | >>>>>>>>>

266620169 tcccaggaaaacataaccctgccccttccagaccagccagtggtgtttaaccacgtctat 266620228

000000181 N I N V P S T S L C S V D L D L P G G P 000000240

>>>>>>>>> | | | | | | | | | | | | | | | | | | | | >>>>>>>>>

266620229 aacatcaatgtcccctcgacctccctctgctctgtggaccttgatttgccaggcgggcct 266620288

000000241 E V K H E T P L K E I Q N M E H I E H T 000000300

>>>>>>>>> | | | | | | | | | | | | | | | | | | | | >>>>>>>>>

266620289 gaggtcaaacacgagacaccactgaaagaaatacagaacatggagcatatagagcacact 266620348

000000301 E D G D N Q I V F T H R I T I P K Q A C 000000360

>>>>>>>>> | | | | | | | | | | | | | | | | | | | | >>>>>>>>>

266620349 gaagatggcgacaaccagattgtgttcacacatcgcatcactatcccgaagcaggcatgc 266620408

000000361 S C R N Q L L D L K T I L N R L E M L E 000000420

>>>>>>>>> | | | | | | | | | | | | | | | | | | | | >>>>>>>>>

266620409 agctgcagaaaccagctgctggatcttaaaactatcctgaataggcttgagatgctggag 266620468

000000421 L E L S S L R E Q C S S G A G C C G A Q 000000480

>>>>>>>>> | | | | | | | | | | | | | | | | | | | | >>>>>>>>>

266620469 ttggaattgtcaagtctaagagagcagtgttccagtggggctggctgctgtggagctcag 266620528

000000481 V 000000483

>>>>>>>>> | >>>>>>>>>

266620529 gtt 266620531

000000484 T G E I S T K P Y C N G H G N W S T D T 000000543

>>>>>>>>> | | | | P | | | | | | | | | | | | | | | >>>>>>>>>

266620657 acaggtgaaattcccaccaaaccttactgtaatggccatgggaactggagcaccgacaca 266620716

000000544 C S C I C E P G W K G H N C S D P E C P 000000603

>>>>>>>>> | | | | | | | | | | | | | | | | | | | | >>>>>>>>>

266620717 tgcagctgcatctgtgagcctggatggaaaggccataactgcagcgatcccgagtgcccc 266620776

000000604 G D C Q D Q G R C L N G R C E C F E G F 000000663

>>>>>>>>> | | | | | | | | | | | | | | | | | | | | >>>>>>>>>

266620777 ggtgactgtcaggaccagggtcgctgcctgaatggcagatgtgaatgctttgagggtttt 266620836

000000664 G G E D C S N E L C L L D C G D Y G H C 000000723

>>>>>>>>> | | | | | | | | | | | | | | | | | | | | >>>>>>>>>

266620837 ggtggtgaagactgcagtaacgagctctgcctgctggactgtggtgattatggtcactgt 266620896

000000724 V N G V C L C E E G F S G E D C S Q T S 000000783

>>>>>>>>> | | | | | | | | | | | | | | | | | | | | >>>>>>>>>

266620897 gtcaacggtgtctgcctatgtgaggagggcttcagcggggaggactgcagccagacaagc 266620956

000000784 C L N N C F G R G S C H E D E C V C D E 000000843

>>>>>>>>> | | | | | | | | | | | | | | | | | | | | >>>>>>>>>

266620957 tgcctcaacaactgcttcggccgtggaagttgtcatgaagatgaatgcgtttgtgatgag 266621016

000000844 P W T G Y D C S E I I C P N D C Y D H G 000000903

>>>>>>>>> | | | | | | | | | | | | | | | | | | | | >>>>>>>>>

266621017 ccatggactggatatgactgctctgaaatcatctgtccaaatgactgttacgatcatggc 266621076

000000904 R C I N G T C E C D E G Y T G E D C G D 000000963

>>>>>>>>> | | | | | | | | | | | | | | | | | | | | >>>>>>>>>

266621077 cggtgcattaatggcacctgtgagtgtgatgaaggctacactggggaagactgcggtgat 266621136

000000964 L S C P S H C N N H G M C L N G Q C V C 000001023

>>>>>>>>> | | | | | | | | | | | | | | | | | | | | >>>>>>>>>

266621137 ttatcctgtcctagtcattgtaacaaccatggcatgtgcttgaatggccagtgtgtgtgt 266621196

000001024 Q T G Y S G E D C S K R S C P K N C N E 000001083

>>>>>>>>> | | | | | | | | | | | | | | | | | | | | >>>>>>>>>

266621197 cagactggctacagtggagaggattgctcaaagcgctcctgtccaaagaactgcaacgag 266621256

000001084 K G H C F N G K C I C D P G H E G 000001134

>>>>>>>>> | | | | | | | | | | | | | | | | | >>>>>>>>>

266621257 aaaggccattgtttcaatggaaaatgtatctgtgatccaggacacgaaggt 266621307

000001144 S I L S C P D N C N S R G E C I N G E C 000001203

>>>>>>>>> | | | X | | | | | | | | | | | | | | | | >>>>>>>>>

266621318 tcaatcctctnatgccccgacaactgcaatagcaggggcgagtgcattaatggagaatgt 266621377

000001204 V C D A G Y Q G E D C S V L A C P N N C 000001263

>>>>>>>>> | | | | | | | | | | | | | | | | | | | | >>>>>>>>>

266621378 gtgtgcgatgctggctaccagggtgaagactgcagtgttctcgcctgtcctaacaattgt 266621437

000001264 L D R G N C V N G Q C M C D K G Y S G E 000001323

>>>>>>>>> | | | | | | | | | | | | | | | | | | | | >>>>>>>>>

266621438 ctggaccgcggaaactgtgttaatggacagtgcatgtgtgacaaaggctacagcggggag 266621497

000001324 D C N I K T C P K N C M G R G D C V D G 000001383

>>>>>>>>> | | | | | | | | | | | | | | | | | | | | >>>>>>>>>

266621498 gactgcaatattaagacctgtccaaagaattgcatgggacggggggattgcgtggatggc 266621557

000001384 K C M C F T G F K G K D C G E M T C P R 000001443

>>>>>>>>> | | | | | | | | | | | | | | | | | | | | >>>>>>>>>

266621558 aagtgtatgtgcttcactggttttaaaggcaaagactgtggtgagatgacctgccccaga 266621617

000001444 D C M N Q G H C E N G K C A C H N G Y T 000001503

>>>>>>>>> | | | | | | | | | | | | | | | | | | | | >>>>>>>>>

266621618 gactgtatgaaccaggggcactgtgagaatggaaaatgtgcttgccataatggttacacg 266621677

000001504 G E D C S Q K T C P K N C H N R G Y C I 000001563

>>>>>>>>> | | | | | | | | | | | | | | | | | | | | >>>>>>>>>

266621678 ggcgaagattgcagtcagaagacgtgtcccaaaaactgtcataacagaggctactgcatt 266621737

000001564 D G D C V C Y E G F T G T D C S I I A C 000001623

>>>>>>>>> | | | | | | | | | | | | | | | | | | | | >>>>>>>>>

266621738 gatggtgactgtgtatgttatgaaggtttcactgggacagactgctccataattgcttgc 266621797

000001624 P S D C L N Q G H C K N G V C V C E E G 000001683

>>>>>>>>> | | | | | | | | | | | | | | | | | | | | >>>>>>>>>

266621798 ccaagtgactgcctaaaccagggacactgtaaaaatggagtatgcgtctgtgaagaagga 266621857

000001684 F T G E D C S A 000001707

>>>>>>>>> | | | | | | | | >>>>>>>>>

266621858 ttcacaggagaggactgttcagcg 266621881

000001708 V S P P K D L T V V E V S P E A V D L S 000001767

>>>>>>>>> | | | | | | | | | | | | | | | | | | | | >>>>>>>>>

266622073 gtctcaccacccaaggacctaacagtagtggaggtgtcaccggaggccgtggacctgtcc 266622132

000001768 W E N E M R V S E Y L I K Y A P T V P G 000001827

>>>>>>>>> | | | | | | | | | | | | | | | | | | | | >>>>>>>>>

266622133 tgggagaatgagatgcgtgtttcagagtacttaataaagtatgcacccactgttcctgga 266622192

000001828 G L E L D M Q V P G D Q K K A T L L E L 000001887

>>>>>>>>> | | | | | | | | | | | | | | | | | | | | >>>>>>>>>

266622193 ggcctggagctggacatgcaggtacctggggaccagaagaaggccacattgctagagctt 266622252

000001888 E P G V E Y L I S V Y A L L N N K K S V 000001947

>>>>>>>>> | | | | | | | | | | | | | | | | | | | | >>>>>>>>>

266622253 gaacctggagtggaatacctgatcagtgtctatgctttgctcaacaacaagaagagtgtt 266622312

000001948 P V N A R V A L 000001971

>>>>>>>>> | | | | | | | | >>>>>>>>>

266622313 cctgtcaatgctagagtagctttg 266622336

000001972 D L P K P D G L K F K S V R D T S V Q V 000002031

>>>>>>>>> | | | | | | | | | | | | | | | | | | | | >>>>>>>>>

266622418 gatttgccaaagcctgacggactcaagttcaagtcagtgcgagatacatcagtgcaggta 266622477

000002032 E W D P I D F P F D G W N L I F R N M 000002088

>>>>>>>>> | | | | | | | | | | | | | | | | | | | >>>>>>>>>

266622478 gagtgggacccaattgactttcccttcgatggctggaacctcatctttagaaacatg 266622534

000002089 K E E D G E I L N F L S H P E T M F E Q 000002148

>>>>>>>>> | | | | | | | | | | | | | | | | | | | | >>>>>>>>>

266622909 aaagaagaggatggcgaaattctgaactttcttagccatccagagaccatgtttgagcag 266622968

000002149 S G L G P G Q E Y E V K L E V V K N N K 000002208

>>>>>>>>> | | | | | | | | | | | | | | | | | | | | >>>>>>>>>

266622969 tcaggcttgggccctggccaagagtatgaggtcaagctggaggttgttaaaaacaacaaa 266623028

000002209 R G P P A S K N V I T 000002241

>>>>>>>>> | | | | | | | | | | | >>>>>>>>>

266623029 cgcggaccacctgcttcgaagaatgtgatcaca 266623061

000002245 I D S P N H V D I R D V T D T T A L V T 000002304

>>>>>>>>> | | | | | | | | | | | | | | | | | | | | >>>>>>>>>

266623944 atcgattccccaaaccatgtagacatccgtgacgtgacagacaccacggcactggtgacc 266624003

000002305 W M P P V A E V E E V S I S Y G P S S N 000002364

>>>>>>>>> | | | | | | | | | | | | | | | | | | | | >>>>>>>>>

266624004 tggatgccgcctgttgcagaggttgaggaggtgagtatttcttatggacccagctccaac 266624063

000002365 P A D R N M V E L S S T E T Q Y H L G G 000002424

>>>>>>>>> | | | | | | | | | | | | | | | | | | | | >>>>>>>>>

266624064 cccgcagaccgcaacatggtggagctgtcatccactgaaacccagtaccacctgggaggc 266624123

000002425 L H P D T Q Y E V S L T A H K G E W S S 000002484

>>>>>>>>> | | | | | | | | | | | | | | | | | | | | >>>>>>>>>

266624124 ctccaccctgacacccagtatgaagtttctctgacagcccacaagggagagtggagcagt 266624183

000002485 N P V H E S F L T 000002511

>>>>>>>>> | | | | | | | | | >>>>>>>>>

266624184 aatcccgtccatgagtcttttctgaca 266624210

000002512 E L D A P K H L K T A E I T D E S I T L 000002571

>>>>>>>>> | | | | | | | | | | | | | | | | | | | | >>>>>>>>>

266624306 gagctggatgctccaaagcacttgaagacagctgaaataacagatgagagcatcactctt 266624365

000002572 E W E N S R A Q V D N Y R I K Y G P L S 000002631

>>>>>>>>> | | | | | | | | | | | | | | | | | | | | >>>>>>>>>

266624366 gagtgggaaaacagtcgagctcaggttgacaactaccgcatcaagtatggacctttgtct 266624425

000002632 G G E H R E L L F T P G A K D Y T H A K 000002691

>>>>>>>>> | | | | | | | | | | | | | | | | | | | | >>>>>>>>>

266624426 ggaggagagcacagggaactactctttaccccaggagccaaagactacacccatgccaaa 266624485

000002692 I T 000002697

>>>>>>>>> | | >>>>>>>>>

266624486 atcact 266624491

000002698 G L R A G T E Y G M G V T A V K D E R E 000002757

>>>>>>>>> | | | | | | | | | | | | | | | | | | | | >>>>>>>>>

266625848 ggcctgagagccggcacagagtatgggatgggtgtaacagcagtgaaagatgagcgggag 266625907

000002758 S L P T T T N A V T 000002787

>>>>>>>>> | | | | | | | | | | >>>>>>>>>

266625908 agtttgccaacaaccaccaatgcagtgacg 266625937

000002788 A L D S P K D L I V T K V T E T T M L L 000002847

>>>>>>>>> | | | | | | | | | | | | | | | | | | | | >>>>>>>>>

266626510 gcactggattctccaaaggacctaattgttaccaaggtgacagagaccaccatgttgctg 266626569

000002848 E W R H P Q A K L D S Y R L V Y V S A D 000002907

>>>>>>>>> | | | | | | | | | | | | | | | | | | | | >>>>>>>>>

266626570 gaatggagacacccccaggctaaactggactcttacaggctggtttatgtatctgccgat 266626629

000002908 G H R S E E V L P G D L K S Y S L M E L 000002967

>>>>>>>>> | | | | | | | | | | | | | | | | | | | | >>>>>>>>>

266626630 ggccacagaagtgaggaggtactgccgggtgatttaaagtcatacagcctgatggagctc 266626689

000002968 T P G M L Y T I S I N T E R G S R T S A 000003027

>>>>>>>>> | | | | | | | | | | | | | | | | | | | | >>>>>>>>>

266626690 acccctggcatgctttacaccatcagcatcaacactgagaggggcagcaggaccagtgca 266626749

000003028 P I T I S A F T 000003051

>>>>>>>>> | | | | | | | | >>>>>>>>>

266626750 cccataaccatctcagcattcaca 266626773

000003052 E E E K P V V T H F T I S D V S W D S F 000003111

>>>>>>>>> | | | | | | | | | | | | | | | | | | | | >>>>>>>>>

266628097 gaggaggagaagccagttgtgacccatttcaccatcagtgatgtgtcctgggacagcttt 266628156

000003112 H L S W S T K D G A F Q A F L I K V T D 000003171

>>>>>>>>> | | | | | | | | | | | | | | | | | | | | >>>>>>>>>

266628157 catttgtcctggtctaccaaggacggggccttccaggccttcctgatcaaggtcacggat 266628216

000003172 A E T S S D V Q N H T L P A A A Q S L A 000003231

>>>>>>>>> | | | | | | | | | | | | | | | | | | | | >>>>>>>>>

266628217 gcagaaacgagttctgatgtccagaaccataccctgcctgctgctgctcaaagtctcgcc 266628276

000003232 I S D L S A T T W Y R V N L Y G L Y R G 000003291

>>>>>>>>> | | | | | | | | | | | | | | | | | | | | >>>>>>>>>

266628277 atctctgatctctctgctactacctggtacagagtcaatctgtatgggttgtacaggggg 266628336

000003292 A L L A P V Y A D T I T 000003327

>>>>>>>>> | | | | | | | | | | | | >>>>>>>>>

266628337 gctctcttagctccagtctatgctgacactatcaca 266628372

000003328 E A E P E I Q A L L V S E V T P E S F W 000003387

>>>>>>>>> | | | | | | | | | | | | | | | | | | | | >>>>>>>>>

266629898 gaggctgaaccagagatccaggccctcctggtctctgaagtcacccctgagagtttttgg 266629957

000003388 L T W M A E E D A L D T F V I M V S P A 000003447

>>>>>>>>> | | | | | | | | | | | | | | | | | | | | >>>>>>>>>

266629958 ctgacgtggatggctgaggaggatgctttggacacctttgtgataatggtcagcccagct 266630017

000003448 D D P G H P K E L V L G S E K R S V A I 000003507

>>>>>>>>> | | | | | | | | | | | | | | | | | | | | >>>>>>>>>

266630018 gatgacccaggtcatccaaaagagcttgtgctgggaagtgagaagcgaagcgtagccatt 266630077

000003508 A N L T E D T E Y R I E M F G L S F G R 000003567

>>>>>>>>> | | | | | | | | | | | | | | | | | | | | >>>>>>>>>

266630078 gcaaaccttacagaggatacagagtacagaatcgaaatgttcggcctcagttttggaaga 266630137

000003568 S T K S V Q E S V R T 000003600

>>>>>>>>> | | | | | | | | | | | >>>>>>>>>

266630138 agcaccaaatctgtgcaggaaagtgtcagaaca 266630170

000003601 D L A P P K G I R F S D V T D T S T T V 000003660

>>>>>>>>> | | | | | | | | | | | | | | | | | | | | >>>>>>>>>

266632762 gacctggcccctcctaaaggcatacgcttctctgatgtgactgacacatccaccactgtt 266632821

000003661 H W G A P R V R V D S Y Q I T Y V P A H 000003720

>>>>>>>>> | | | | | | | | | | | | | | | | | | | | >>>>>>>>>

266632822 cactggggggctccaagagttcgagtggacagctatcagatcacatatgtccctgctcat 266632881

000003721 G 000003723

>>>>>>>>> | >>>>>>>>>

266632882 gga 266632884

000003724 G N A K T L T V D G S K S Q T M L P N L 000003783

>>>>>>>>> | | | | | | | | | | | | | | | | | | | | >>>>>>>>>

266633504 ggtaatgctaagacactcacagtggatggttccaagtctcagaccatgctgcccaacctg 266633563

000003784 T P G V T Y E V T I V A V K G P R E S L 000003843

>>>>>>>>> | | | | | | | | | | | | | | | | | | | | >>>>>>>>>

266633564 actcccggagtcacctatgaggtcacaattgttgctgtcaaagggccacgagagagtctg 266633623

000003844 P A S D S I T 000003864

>>>>>>>>> | | | | | | | >>>>>>>>>

266633624 ccagcatcagacagcatcacc 266633644

000003865 T A L D K P R G L V S I N I T D T G A L 000003924

>>>>>>>>> | | | | | | | | | | | | | | | | | | | | >>>>>>>>>

266633763 acagctctggataaacctcgtggattggtttctataaacatcacagatactggagctctg 266633822

000003925 L R W Q P A I A T I D G Y V I T Y S A D 000003984

>>>>>>>>> | | | | | | | | | | | | | | | | | | | | >>>>>>>>>

266633823 ttgcgctggcagccagctattgctacaatagatggttacgtcatcacgtacagcgcagac 266633882

000003985 G 000003987

>>>>>>>>> | >>>>>>>>>

266633883 gga 266633885

000003988 V D P V M E R V S G N V M E F E M S S L 000004047

>>>>>>>>> | | | | | | | | | | | | | | | | | | | | >>>>>>>>>

266634069 gtggatccagtgatggaacgtgtttctggaaatgtgatggagtttgagatgagctcattg 266634128

000004048 V P A T R Y T V K V F A A R D L A K S T 000004107

>>>>>>>>> | | | | | | | | | | | | | | | | | | | | >>>>>>>>>

266634129 gtgcctgcaacacgttacaccgtgaaggtgtttgctgccagagacttggccaagagcaca 266634188

000004108 A T T T E F T T 000004131

>>>>>>>>> | | | | | | | | >>>>>>>>>

266634189 gctacaaccactgagttcacaacc 266634212

000004132 D V D T P S H L A A S N V Q T E S A M L 000004191

>>>>>>>>> | | | | | | | | | | | | | | | | | | | | >>>>>>>>>

266634359 gatgtggacacgcccagtcatttggcagccagtaatgtccaaacagagagtgccatgttg 266634418

000004192 T W K A P R A G I T G Y I L S F E S V D 000004251

>>>>>>>>> | | | | | | | | | | | | | | | | | | | | >>>>>>>>>

266634419 acctggaaggcaccacgtgctggaatcactgggtacatcctcagctttgagtctgttgat 266634478

000004252 G A V R 000004263

>>>>>>>>> | | | | >>>>>>>>>

266634479 ggcgctgtccgt 266634490

000004264 E V V L S P T A V S Y N M A Q L S A S T 000004323

>>>>>>>>> | | | | | | | | | | | | | | | | | | | | >>>>>>>>>

266634559 gaagtggtcctgagccccaccgctgtgtcctacaacatggctcagttaagtgcttccaca 266634618

000004324 D Y S V K L Q A I A G P K R S R V V T A 000004383

>>>>>>>>> | | | | | | | | | | | | | | | | | | | | >>>>>>>>>

266634619 gattattccgtcaagctgcaggccattgccggtccaaagaggagcagagtcgttacagcc 266634678

000004384 V F K T 000004395

>>>>>>>>> | | | | >>>>>>>>>

266634679 gtcttcaaaacc 266634690

000004399 G V Q Y R H P R D C S Q V I L N G D G S 000004458

>>>>>>>>> | | | | | | | | | | | | | | | | | | | | >>>>>>>>>

266635014 ggtgtgcagtacagacatcctagagactgttcccaggttatcctgaatggtgatggttct 266635073

000004459 S G L Y T I F L S G D E N Q P L Q V Y C 000004518

>>>>>>>>> | | | | | | | | | | | | | | | | | | | | >>>>>>>>>

266635074 tcgggcctgtacaccatcttcctcagtggcgatgagaaccagccactccaagtctactgt 266635133

000004519 D M N T D G G G W M 000004548

>>>>>>>>> | | | | | | | | | | >>>>>>>>>

266635134 gacatgaacactgatggtggtggatggatg 266635163

000004549 V F L R R Q S G K L D F F R N W K N Y T 000004608

>>>>>>>>> | | | | | | | | | | | | | | | | | | | | >>>>>>>>>

266635666 gtttttctcagacggcaaagtgggaaattggattttttccgcaactggaagaattacaca 266635725

000004609 A G F G D I N D E F W L 000004644

>>>>>>>>> | | | | | | | | | | | | >>>>>>>>>

266635726 gctgggtttggagatataaatgatgaattttggctg 266635761

000004645 G L S N L N K I T A A A Q Y E L R V D L 000004704

>>>>>>>>> | | | | | | | | | | | | | | | | | | | | >>>>>>>>>

266635864 ggtctgtccaacctgaataagatcacagcagcagctcagtatgaactcagagtggatctg 266635923

000004705 R D K G E T A F A Q Y D R F S V S E S R 000004764

>>>>>>>>> | | | | | | | | | | | | | | | | | | | | >>>>>>>>>

266635924 agggataaaggtgaaacggcctttgctcagtatgacaggttctctgtctctgagtctcgg 266635983

000004765 S R Y K V H I G G Y S G T A 000004806

>>>>>>>>> | | | | | | | | | | | | | | >>>>>>>>>

266635984 agccgctacaaagtccacattggaggctacagtgggacagca 266636025

000004807 G D S M T Y H H G R P F S T Y D N D N D 000004866

>>>>>>>>> | | | | | | | | | | | | | | | | | | | | >>>>>>>>>

266636623 ggggattccatgacctaccaccatggccgcccattttccacctacgacaatgacaacgac 266636682

000004867 I A V T N C A L S Y K G A F W Y K N C H 000004926

>>>>>>>>> | | | | | | | | | | | | | | | | | | | | >>>>>>>>>

266636683 attgcggtcaccaactgcgccctgtcatataagggagcgttctggtacaaaaactgccac 266636742

000004927 R V N L M G R Y G D N S H S K 000004971

>>>>>>>>> | | | | | | | | | | | | | | | >>>>>>>>>

266636743 cgggtcaacctcatgggacgatatggagataatagccacagcaag 266636787

000004972 G V N W F H W K G H E H S I E F A E M K 000005031

>>>>>>>>> | | | | | | | | | | | | | | | | | | | | >>>>>>>>>

266637046 ggtgtgaactggttccactggaaaggtcatgagcactcaattgagtttgctgagatgaag 266637105

000005032 L R P S N F R N L E G R R K R S 000005079

>>>>>>>>> | | | | | | | | | | | | | | | | >>>>>>>>>

266637106 ctgaggccatccaactttagaaacctggagggaagacggaaacgctcc 266637153

Takifugu tenascin-CB:

00000001 L L L A L L S L L N A G L V K K I L R H 00000060

<<<<<<<< | | | | | | | | | | | | | | | | | | | | <<<<<<<<

58561201 ctcctgcttgctttgctcagcttattgaacgccggccttgtgaagaaaattctgcgtcat 58561142

00000061 R R Q T L A A P E E Y N A T V P G A G H 00000120

<<<<<<<< | | | | | | | | | | | | | | | | | | | | <<<<<<<<

58561141 cgtcgccagactttggccgcccctgaggaatataacgccactgttcccggcgcaggccat 58561082

00000121 P V V F N H V Y N I N V P A S S L C S V 00000180

<<<<<<<< | | | | | | | | | | | | | | | | | | | | <<<<<<<<

58561081 cccgtcgtgttcaaccacgtctacaacatcaacgttcctgccagttccctgtgctcggtc 58561022

00000181 S L D S P D S A E L E A Q D G P L S S G 00000240

<<<<<<<< | | | | | | | | | | | | | | | | | | | | <<<<<<<<

58561021 agcctggactcgcctgacagcgcggagctggaagcccaggacgggccgctttcctcaggc 58560962

00000241 H Q T T E H T V D G E N Q I I F T H R I 00000300

<<<<<<<< | | | | | | | | | | | | | | | | | | | | <<<<<<<<

58560961 catcaaaccaccgagcacaccgtggacggggagaaccagatcatcttcacccatcgtatc 58560902

00000301 N I P R Q A C G C S D D L L N L R D L M 00000360

<<<<<<<< | | | | | | | | | | | | | | | | | | | | <<<<<<<<

58560901 aacatacctcggcaggcctgcggctgctccgacgacttgctcaacctaagagacctcatg 58560842

00000361 S R L E M L E G E V S A L K D Q C N G E 00000420

<<<<<<<< | | | | | | | | | | | | | | | | | | | | <<<<<<<<

58560841 agccgcttggagatgcttgaaggggaagtttcagcgctgaaagatcagtgtaacggcgag 58560782

00000421 R M C C G S Q V T 00000447

<<<<<<<< | | | | | | | | | <<<<<<<<

58560781 agaatgtgctgcggatcgcaagtgaca 58560755

00000448 G E V A I R P Y C N G H G N Y S S D T C 00000507

<<<<<<<< | | | | | | | | | | | | | | | | | | | | <<<<<<<<

58560654 ggtgaagtggccatcagaccttactgcaacggccatggaaactacagcagcgacacctgc 58560595

00000508 S C M C E P G W R G T N C T E L D C P G 00000567

<<<<<<<< | | | | | | | | | | | | | | | | | | | | <<<<<<<<

58560594 agctgcatgtgtgaaccaggctggaggggaaccaactgcaccgaactggactgccccggg 58560535

00000568 N C Q D Q G R C V D G K C Q C L K G F G 00000627

<<<<<<<< | | | | | | | | | | | | | | | | | | | | <<<<<<<<

58560534 aactgccaggatcagggacgctgcgttgatggaaaatgccaatgcctcaaaggcttcggg 58560475

00000628 G E N C T A E V C P V D C G A H G R C V 00000687

<<<<<<<< | | | | | | | | | | | | | | | | | | | | <<<<<<<<

58560474 ggggaaaactgcacggccgaggtctgtcccgtggactgtggagcccacggcaggtgcgtc 58560415

00000688 G A I C V C S E G F F G E D C S K T K C 00000747

<<<<<<<< | | | | | | | | | | | | | | | | | | | | <<<<<<<<

58560414 ggcgccatctgtgtctgttccgaaggcttcttcggcgaggactgctccaaaaccaagtgc 58560355

00000748 L N N C R A R G R C D A G V C V C D E P 00000807

<<<<<<<< | | | | | | | | | | | | | | | | | | | | <<<<<<<<

58560354 ctgaacaactgccgggctcggggccgctgcgatgctggagtctgcgtgtgcgatgagccc 58560295

00000808 W S G A D C S S L L C P K D C R S Q G R 00000867

<<<<<<<< | | | | | | | | | | | | | | | | | | | | <<<<<<<<

58560294 tggagtggcgccgactgctcgagcctcctctgccccaaagactgccgctcccagggacgc 58560235

00000868 C V N G T C Y C D E G Y A G E D C G E R 00000927

<<<<<<<< | | | | | | | | | | | | | | | | | | | | <<<<<<<<

58560234 tgcgtgaacggcacctgctactgcgatgagggctacgccggggaggactgtggcgagcgc 58560175

00000928 A C P G K C Y G N G F C V D G R C V C I 00000987

<<<<<<<< | | | | | | | | | | | | | | | | | | | | <<<<<<<<

58560174 gcgtgccctggtaagtgctatggcaacggcttctgtgtggacggccggtgcgtgtgcatt 58560115

00000988 A G Y S G E D C S Q L N C L N D C N G R 00001047

<<<<<<<< | | | | | | | | | | | | | | | | | | | | <<<<<<<<

58560114 gctggctacagtggcgaagactgctcccagctcaactgcctgaacgactgcaacggccga 58560055

00001048 G S C F N G L C I C D T G Y Q G E D C S 00001107

<<<<<<<< | | | | | | | | | | | | | | | | | | | | <<<<<<<<

58560054 ggcagctgcttcaacgggctgtgtatctgtgacacgggctaccagggcgaagactgcagc 58559995

00001108 Q L A C V N N C N S R G Q C I N G Q C S 00001167

<<<<<<<< | | | | | | | | | | | | | | | | | | | | <<<<<<<<

58559994 cagttagcatgtgtgaacaactgtaacagcagaggccagtgcatcaacgggcagtgctcc 58559935

00001168 C D A G F H G E D C G E L S C P N S C L 00001227

<<<<<<<< | | | | | | | | | | | | | | | | | | | | <<<<<<<<

58559934 tgcgacgctgggttccacggagaagactgcggcgagctctcctgtcccaatagctgcttg 58559875

00001228 N R G R C V N G Q C V C E E G Y A G E D 00001287

<<<<<<<< | | | | | | | | | | | | | | | | | | | | <<<<<<<<

58559874 aacagggggcgctgcgttaacgggcagtgcgtgtgcgaagagggctacgccggcgaggac 58559815

00001288 C R A M T C P S N C Y G R G E C T E G R 00001347

<<<<<<<< | | | | | | | | | | | | | | | | | | | | <<<<<<<<

58559814 tgcagggccatgacctgcccctctaactgctacggccggggggagtgcaccgagggtcgc 58559755

00001348 C V C H T G F T G D D C S K L S C P N S 00001407

<<<<<<<< | | | | | | | | | | | | | | | | | | | | <<<<<<<<

58559754 tgcgtgtgccacacgggcttcaccggggacgactgcagcaaactgagctgccccaacagc 58559695

00001408 C Q D R G R C V D G Q C V C D E G F A G 00001467

<<<<<<<< | | | | | | | | | | | | | | | | | | | | <<<<<<<<

58559694 tgccaggatcgtggcaggtgcgtggacgggcagtgcgtctgcgatgaaggcttcgccggg 58559635

00001468 E D C S R K A C P N D C L A R G Y C D D 00001527

<<<<<<<< | | | | | | | | | | | | | | | | | | | | <<<<<<<<

58559634 gaggactgcagccggaaagcttgtcccaacgactgcctggcacgaggctactgcgatgac 58559575

00001528 G K C V C Q E G Y A G D D C S A L T C P 00001587

<<<<<<<< | | | | | | | | | | | | | | | | | | | | <<<<<<<<

58559574 ggcaagtgcgtctgccaggaaggctacgcaggagacgactgctccgcgctcacctgccct 58559515

00001588 A N C N N R G R C V S G R C A C E S G Y 00001647

<<<<<<<< | | | | | | | | | | | | | | | | | | | | <<<<<<<<

58559514 gccaactgtaacaacagggggcgctgcgtgagtggaaggtgcgcgtgtgagagcggatac 58559455

00001648 E G E S C A E R S C L N G C R D N G R C 00001707

<<<<<<<< | | | | | | | | | | | | | | | | | | | | <<<<<<<<

58559454 gaaggggaaagctgtgcggagcggagctgcctcaacggctgccgggacaacggccgctgc 58559395

00001708 L N G Q C L C D E G Y V G E D C S E 00001761

<<<<<<<< | | | | | | | | | | | | | | | | | | <<<<<<<<

58559394 ctcaacggccagtgcctctgcgatgagggctacgtgggggaagactgctcggaa 58559341

00001762 V S P P T D L V V S E V T S D T V D L S 00001821

<<<<<<<< | | | | | | | | | | | | | | | | | | | | <<<<<<<<

58559024 gtgtctcctcccactgacctggtggtctccgaggtcaccagcgacaccgtggatctgtcc 58558965

00001822 W R N Q M L V T E Y L V T Y T P T R P G 00001881

<<<<<<<< | | | | | | | | | | | | | | | | | | | | <<<<<<<<

58558964 tggcgcaaccagatgttggtgacggagtaccttgtgacgtacacccccacgagacccggc 58558905

00001882 G L L Q E F T V S G D K T A A T V P E L 00001941

<<<<<<<< | | | | | | | | | | | | | | | | | | | | <<<<<<<<

58558904 ggtcttctccaggagttcaccgtgtcgggggataaaactgcagccacggtgccagagctg 58558845

00001942 E P G L E Y V I K V Y A I L S N K R S V 00002001

<<<<<<<< | | | | | | | | | | | | | | | | | | | | <<<<<<<<

58558844 gagcccggtcttgagtatgtgatcaaagtttacgccatcctgagcaacaagaggagcgtc 58558785

00002002 P V S A R V A T 00002025

<<<<<<<< | | | | | | | | <<<<<<<<

58558784 cccgtcagcgccagggtggctaca 58558761

00002026 D F P R P E G V I F K S V S E T S V E V 00002085

<<<<<<<< | | | | | | | | | | | | | | | | | | | | <<<<<<<<

58558667 gattttcctcgacctgagggtgtaatattcaaatctgttagtgagacttcagtggaggtc 58558608

00002086 M W D Q L D I P F D G W E I Y F R N T 00002142

<<<<<<<< | | | | | | | | | | | | | | | | | | | <<<<<<<<

58558607 atgtgggaccagctggacatccccttcgatggctgggaaatctatttccgtaacacg 58558551

00002143 K E E N G Q V K S T V P S S Q N Q F V Q 00002202

<<<<<<<< | | | | | | | | | | | | | | | | | | | | <<<<<<<<

58558020 aaagaagaaaacggtcaagtcaagagcaccgttccatcctcccagaaccagttcgtccag 58557961

00002203 S G L G P G Q E Y E I A I N M I K N N T 00002262

<<<<<<<< | | | | | | | | | | | | | | | | | | | | <<<<<<<<

58557960 tcaggcctcggaccggggcaggagtacgagatcgccatcaacatgattaagaacaacacc 58557901

00002263 R G P Q S K K K V T T 00002295

<<<<<<<< | | | | | | | | | | | <<<<<<<<

58557900 agggggccccagagcaagaagaaggtcacgact 58557868

00002299 I D A P Q Q V E V K D V T D T S 00002346

<<<<<<<< | | | | | | | | | | | | | | | | <<<<<<<<

58557365 atcgacgccccccagcaggtggaggtgaaggacgtgacggacacctcg 58557318

00002347 L V G W S Q P V A S V D R I T M F Y G L 00002406

<<<<<<<< | | | | | | | | | | | | | | | | | | | | <<<<<<<<

58557313 ctggtcggctggtctcagccggtggcgtctgtggacagaatcaccatgttctacgggttg 58557254

00002407 T S D P S D T N S V E I F S P D K Q Y S 00002466

<<<<<<<< | | | | | | | | | | | | | | | | | | | | <<<<<<<<

58557253 acctccgacccctccgacacaaacagcgtggagatattctcccccgacaaacagtacagc 58557194

00002467 V D G L R P D T E Y K V T L I S R R G D 00002526

<<<<<<<< | | | | | | | | | | | | | | | | | | | | <<<<<<<<

58557193 gttgacggactgaggccagacaccgagtacaaggtgacgctcatctccaggaggggagac 58557134

00002527 S T S D P V S A S F S T 00002562

<<<<<<<< | | | | | | | | | | | | <<<<<<<<

58557133 tccaccagtgaccccgtcagcgcctccttcagcacg 58557098

00002563 A L D A P T N L Q I V S Q T D Q S I T V 00002622

<<<<<<<< | | | | | | | | | | | | | | | | | | | | <<<<<<<<

58556980 gccctcgatgctcccacaaacctgcagattgtatcccagacggaccagagcatcactgtt 58556921

00002623 Q W T N S K A N V S S Y L V K Y S P I S 00002682

<<<<<<<< | | | | | | | | | | | | | | | | | | | | <<<<<<<<

58556920 cagtggaccaacagcaaggctaacgttagcagctatctggtgaaatacagccccatttct 58556861

00002683 G E S H G E E L F P R Q P G H A T K A T 00002742

<<<<<<<< | | | | | | | | | | | | | | | | | | | | <<<<<<<<

58556860 ggagaaagtcacggcgaggaactcttcccacgtcaaccgggacatgccaccaaagctact 58556801

00002743 L T 00002748

<<<<<<<< | | <<<<<<<<

58556800 ctgact 58556795

00002749 G L R P G T E Y G I G V T A M K N E T E 00002808

<<<<<<<< | | | | | | | | | | | | | | | | | | | | <<<<<<<<

58556717 gggctgcggcctggaaccgaatatggaatcggtgtgactgccatgaagaacgagacagag 58556658

00002809 S L P A T T N A E T 00002838

<<<<<<<< | | | | | | | | | | <<<<<<<<

58556657 agccttccagctaccaccaacgctgaaact 58556628

00002839 E I D P P T N V E E V E S S E T S L T L 00002898

<<<<<<<< | | | | | | | | | | | | | | | | | | | | <<<<<<<<

58556557 gaaatcgaccctcccacaaacgtggaggaggtggagtcctcggagacgtccctcaccctg 58556498

00002899 R W Q K P Q A K F S T Y R L V Y V S R D 00002958

<<<<<<<< | | | | | | | | | | | | | | | | | | | | <<<<<<<<

58556497 aggtggcagaaacctcaggctaagttcagcacctacagactggtgtacgtctccagggac 58556438

00002959 G Q V E E D V I P A S A T S Y V M S N L 00003018

<<<<<<<< | | | | | | | | | | | | | | | | | | | | <<<<<<<<

58556437 ggccaggtcgaggaggacgtgatcccagcctcggcaacttcttacgtcatgtccaacctg 58556378

00003019 T P G M S Y S V T L T A E R G L K R S K 00003078

<<<<<<<< | | | | | | | | | | | | | | | | | | | | <<<<<<<<

58556377 actcctggaatgagctacagcgtgactctgacggcagagaggggtctgaagaggagcaaa 58556318

00003079 P V S H S A T T 00003102

<<<<<<<< | | | | | | | | <<<<<<<<

58556317 ccagtctcccattctgcaacaaca 58556294

00003103 D P S E T Q F S R M E P D E E P G T L T 00003162

<<<<<<<< | | | | | | | | | | | | | | | | | | | | <<<<<<<<

58555937 gacccctctgaaacacagttttccaggatggagcctgatgaggagcccggaacgctgact 58555878

00003163 V S G V T H D G F D V S W N L T A H S I 00003222

<<<<<<<< | | | | | | | | | | | | | | | | | | | | <<<<<<<<

58555877 gtgtcgggcgtcacgcacgatgggtttgacgtctcttggaacctcacagctcacagcatt 58555818

00003223 Y D S F T V E Y T D I R Q L W D M R E V 00003282

<<<<<<<< | | | | | | | | | | | | | | | | | | | | <<<<<<<<

58555817 tatgatagtttcactgtagaatatacggacattcgacaattatgggacatgcgagaagtt 58555758

00003283 P L P G D V T G T S I R G L K A L T E Y 00003342

<<<<<<<< | | | | | | | | | | | | | | | | | | | | <<<<<<<<

58555757 ccgcttccaggagacgtcactggcactagcatccgaggcctgaaggcactcacagaatat 58555698

00003343 Q I K L Y G E I Q N Q R S A L L E A V A 00003402

<<<<<<<< | | | | | | | | | | | | | | | | | | | | <<<<<<<<

58555697 caaataaaactttacggagaaatccaaaatcagagatctgcactacttgaagctgtcgca 58555638

00003403 V T 00003408

<<<<<<<< | | <<<<<<<<

58555637 gttaca 58555632

00003409 A E Q L E P V V T N L T V S D I A W D G 00003468

<<<<<<<< | | | | | | | | | | | | | | | | | | | | <<<<<<<<

58554294 gccgagcagctggagcctgtagtgaccaacctcaccgtctctgacattgcgtgggacggc 58554235

00003469 F T V S W S P A G G E F D S F V I E I T 00003528

<<<<<<<< | | | | | | | | | | | | | | | | | | | | <<<<<<<<

58554234 ttcacagtgtcctggagccccgcaggtggagaatttgacagctttgtcattgagataaca 58554175

00003529 N L G N L E E S Q N L T L S G G A F S L 00003588

<<<<<<<< | | | | | | | | | | | | | | | | | | | | <<<<<<<<

58554174 aacctggggaaccttgaggagagccagaacctcacgctctccgggggagcgttcagtctg 58554115

00003589 A V S G L N P N T S Y M V G L F G V Y Q 00003648

<<<<<<<< | | | | | | | | | | | | | | | | | | | | <<<<<<<<

58554114 gctgtgtccgggctgaaccccaacaccagctacatggttggactgtttggggtctatcaa 58554055

00003649 D S I L E P V Y T E A T T 00003687

<<<<<<<< | | | | | | | | | | | | | <<<<<<<<

58554054 gactccattcttgaacccgtgtacactgaagccaccaca 58554016

00003688 V D M P A V G K L Y I T N L T S D S F S 00003747

<<<<<<<< | | | | | | | | | | | | | | | | | | | | <<<<<<<<

58553214 gtggacatgcccgcggttggcaaactgtatatcacaaacttaacgtcagacagcttttcg 58553155

00003748 I L W N G T R G E F D G F V L E I I D S 00003807

<<<<<<<< | | | | | | | | | | | | | | | | | | | | <<<<<<<<

58553154 atcctctggaacggcacccggggggagtttgatggctttgtcctggaaataattgattct 58553095

00003808 N W L M E P K E Y N L S H L V R S Y D I 00003867

<<<<<<<< | | | | | | | | | | | | | | | | | | | | <<<<<<<<

58553094 aattggctgatggagccaaaggaatataatttatcccaccttgtgaggtcttatgatata 58553035

00003868 T G L R P T T D Y V A Y L Y G T Y K R S 00003927

<<<<<<<< | | | | | | | | | | | | | | | | | | | | <<<<<<<<

58553034 acagggctcaggccaaccactgactatgtagcctacctctacgggacgtacaagaggtcc 58552975

00003928 R T N A V S I V A S T 00003960

<<<<<<<< | | | | | | | | | | | <<<<<<<<

58552974 cgaacaaacgctgtcagtattgttgcatcaaca 58552942

00003961 A E E P D L S K L V V S N V T S D R F S 00004020

<<<<<<<< | | | | | | | | | | | | | | | | | | | | <<<<<<<<

58552852 gctgaagagcctgatctgtccaagctagttgtttctaacgttacctctgacagattttct 58552793

00004021 L S W Q T G R K P F D N F V V E L R E S 00004080

<<<<<<<< | | | | | | | | | | | | | | | | | | | | <<<<<<<<

58552792 ctatcctggcaaacgggacggaagccgttcgataactttgtcgtagagctcagagagtcc 58552733

00004081 A M P S Q A M G R V V P G D V R S T V M 00004140

<<<<<<<< | | | | | | | | | | | | | | | | | | | | <<<<<<<<

58552732 gccatgccctctcaagcgatgggccgtgttgtgccgggagacgtgcgctccacggtcatg 58552673

00004141 A G L K A S T S Y D I K L Y G S A G A Q 00004200

<<<<<<<< | | | | | | | | | | | | | | | | | | | | <<<<<<<<

58552672 gccggcctcaaagccagcacaagttacgatataaaactgtatggcagcgctggcgcccag 58552613

00004201 N T Q A L F G V A T T 00004233

<<<<<<<< | | | | | | | | | | | <<<<<<<<

58552612 aacacacaggccctgtttggtgtagctaccaca 58552580

00004234 E E V P Q L G L L T A S S V S P H D V S 00004293

<<<<<<<< | | | | | | | | | | | | | | | | | | | | <<<<<<<<

58552502 gaggaggtcccacagttggggctcctaactgcctcctccgtgagtccccacgacgtcagc 58552443

00004294 L T W S T L S G H F D G F V I R V S D P 00004353

<<<<<<<< | | | | | | | | | | | | | | | | | | | | <<<<<<<<

58552442 ttgacctggagcacgctgtcgggccactttgacggctttgtgatccgcgtcagtgacccc 58552383

00004354 Q Q R F D T Q E F R L P R G A R N F T V 00004413

<<<<<<<< | | | | | | | | | | | | | | | | | | | | <<<<<<<<

58552382 cagcagcggtttgacacgcaggagttccgactcccgcgtggagcccgtaactttaccgtc 58552323

00004414 S N L V D A T A Y D V E L Y G I S H D R 00004473

<<<<<<<< | | | | | | | | | | | | | | | | | | | | <<<<<<<<

58552322 tctaacctggtggacgccacggcctatgacgttgaactgtacggtatctctcatgaccgt 58552263

00004474 H T T S V F A H A V T 00004506

<<<<<<<< | | | | | | | | | | | <<<<<<<<

58552262 cacactacctctgtgtttgcccatgctgtcaca 58552230

00004507 A P L P K V E N I T L S H V T P Y G F R 00004566

<<<<<<<< | | | | | | | | | | | | | | | | | | | | <<<<<<<<

58551992 gctccgttacctaaagtggaaaatatcaccctttcccacgttactccctacggcttccgc 58551933

00004567 V S W E V K Q Q L Q Q E D L A P S S G R 00004626

<<<<<<<< | | | | | | | | | | | | | | | | | | | | <<<<<<<<

58551932 gtgtcgtgggaggtgaagcagcagctgcagcaggaggatttagccccctctagtggtcgc 58551873

00004627 F R Y F H V V V T D S G W L L E P Q E F 00004686

<<<<<<<< | | | | | | | | | | | | | | | | | | | | <<<<<<<<

58551872 ttccgctattttcacgtagtggtgacagactcgggctggctgttggaacctcaggagttt 58551813

00004687 S V P G N Q T H L D V T G L I T G I G Y 00004746

<<<<<<<< | | | | | | | | | | | | | | | | | | | | <<<<<<<<

58551812 tctgtgccggggaaccaaactcacctggacgtcacgggccttatcaccggcatcggctat 58551753

00004747 E V R L T G V S E S G L L S R P L T T V 00004806

<<<<<<<< | | | | | | | | | | | | | | | | | | | | <<<<<<<<

58551752 gaggtcaggctgaccggcgtgtccgagtcagggctcctctctcggcccctgacgacagtg 58551693

00004807 A V T 00004815

<<<<<<<< | | | <<<<<<<<

58551692 gcggtgaca 58551684

00004816 E A E P E V E H L F V S D I T D S G F R 00004875

<<<<<<<< | | | | | | | | | | | | | | | | | | | | <<<<<<<<

58550972 gaggccgagccggaggtggaacacctgtttgtctccgatatcacggacagcggtttccgc 58550913

00004876 L S W T S D E D M F D R F V V K I R D G 00004935

<<<<<<<< | | | | | | | | | | | | | | | | | | | | <<<<<<<<

58550912 ctgtcctggacttctgatgaagacatgtttgacagatttgtggtcaaaataagagacggc 58550853

00004936 K R L A H P Q E Y A V R G N E R T T V V 00004995

<<<<<<<< | | | | | | | | | | | | | | | | | | | | <<<<<<<<

58550852 aaaagattagctcaccctcaggagtacgccgtccgaggcaacgagcggacgacggttgta 58550793

00004996 T G L M S G T E Y E I E L Y G V T L D K 00005055

<<<<<<<< | | | | | | | | | | | | | | | | | | | | <<<<<<<<

58550792 actggactcatgagcggcaccgagtacgaaatcgagctttacggtgtcacgttagacaaa 58550733

00005056 R S Q P V T G V A Q T 00005088

<<<<<<<< | | | | | | | | | | | <<<<<<<<

58550732 cgctcccaacccgttaccggagtcgctcagaca 58550700

00005089 G L S T P K G L H F S D V T D S S A V V 00005148

<<<<<<<< | | | | | | | | | | | | | | | | | | | | <<<<<<<<

58550444 ggtctgagcactccgaaggggctccatttctcggacgtgaccgactcctcggctgtggtt 58550385

00005149 H W S M P R S L V D S Y R V T Y V P F E 00005208

<<<<<<<< | | | | | | | | | | | | | | | | | | | | <<<<<<<<

58550384 cactggtccatgcctcgctctctggtagacagctaccgtgtcacctacgtacccttcgaa 58550325

00005209 G 00005211

<<<<<<<< | <<<<<<<<

58550324 gga 58550322

00005212 G S P M T V T V D G G A F E A L L A N M 00005271

<<<<<<<< | | | | | | | | | | | | | | | | | | | | <<<<<<<<

58550244 ggcagcccgatgacggtgacggtggacggcggcgcgttcgaggctctgctcgccaacatg 58550185

00005272 I P G K T Y Q V T V S S V K G L E E S D 00005331

<<<<<<<< | | | | | | | | | | | | | | | | | | | | <<<<<<<<

58550184 attccaggcaaaacgtaccaggtaacggtgagttctgtgaagggtctggaggagagcgac 58550125

00005332 P S M D T V T T 00005355

<<<<<<<< | | | | | | | | <<<<<<<<

58550124 cccagcatggacaccgttaccaca 58550101

00005356 A L D R P Q T L T A L N V T D S S A L L 00005415

<<<<<<<< | | | | | | | | | | | | | | | | | | | | <<<<<<<<

58550031 gctttggatcggccgcagactctgacggcgctcaacgtcaccgactcctccgccctcctg 58549972

00005416 L W Q P C A A T V D S Y V I T Y S A E S 00005475

<<<<<<<< | | | | | | | | | | | | | | | | | | | | <<<<<<<<

58549971 ctgtggcagccgtgcgccgccaccgtggacagctatgtcatcacctacagcgcagagtca 58549912

00005476 V S P I V E H V S G N T V E F E M G S L 00005535

<<<<<<<< | | | | | | | | | | | | | | | | | | | | <<<<<<<<

58549843 gtgtcccccatcgtggagcacgtttctgggaacacggtggagtttgagatgggttctctg 58549784

00005536 V P G T R Y K V G V Y A V K D A L K S N 00005595

<<<<<<<< | | | | | | | | | | | | | | | | | | | | <<<<<<<<

58549783 gttccgggaacgcgctataaagttggagtctacgcggtaaaggatgccctgaagagcaac 58549724

00005596 P T V T E F T T 00005619

<<<<<<<< | | | | | | | | <<<<<<<<

58549723 cccaccgttactgaattcaccacc 58549700

00005620 D V D P P R D L E A V N I Q T D S A T L 00005679

<<<<<<<< | | | | | | | | | | | | | | | | | | | | <<<<<<<<

58549627 gatgtggaccctcctcgggatctggaggccgtcaacatacagaccgacagcgccactctc 58549568

00005680 T W K P P Q A A V T G Y T L T F S S A D 00005739

<<<<<<<< | | | | | | | | | | | | | | | | | | | | <<<<<<<<

58549567 acgtggaaaccgccgcaggccgccgtcaccggctacaccctgaccttctcctccgccgac 58549508

00005740 G V I R 00005751

<<<<<<<< | | | | <<<<<<<<

58549507 ggcgtgatcagg 58549496

00005752 E V V L S P T A S S Y S M V Q L A G S T 00005811

<<<<<<<< | | | | | | | | | | | | | | | | | | | | <<<<<<<<

58549411 gaggtggtgctgagcccgacagcgtcctcctacagcatggttcagctggctggctccacc 58549352

00005812 E Y N V R L Q A I A G A Q R S H H I D T 00005871

<<<<<<<< | | | | | | | | | | | | | | | | | | | | <<<<<<<<

58549351 gagtacaacgtcaggctgcaggccatcgccggggcccagcggagtcaccacatcgacacc 58549292

00005872 A F T T 00005883

<<<<<<<< | | | | <<<<<<<<

58549291 gccttcacgacc 58549280

00005887 G Q L F T R P R D C A Q I W L N G E S T 00005946

<<<<<<<< | | | | | | | | | | | | | | | | | | | | <<<<<<<<

58549163 ggacagttgttcacacggccgcgggactgcgcccagatttggctgaacggagagtcgacc 58549104

00005947 S G L Y S I Y V G G E E S Q P L Q V Y C 00006006

<<<<<<<< | | | | | | | | | | | | | | | | | | | | <<<<<<<<

58549103 tctggcctgtactccatctatgtcgggggggaggagagccagcccctccaggtttactgt 58549044

00006007 D M A T D G G G W T 00006036

<<<<<<<< | | | | | | | | | | <<<<<<<<

58549043 gacatggccacagacggtggaggctggacg 58549014

00006037 V F L R R Q N G K L E F F R N W K N Y T 00006096

<<<<<<<< | | | | | | | | | | | | | | | | | | | | <<<<<<<<

58548921 gttttcctgagacgccagaatgggaagctggaattcttcaggaactggaagaactacacg 58548862

00006097 A G F G N M N D E F W 00006129

<<<<<<<< | | | | | | | | | | | <<<<<<<<

58548861 gccggcttcgggaacatgaacgatgagttctgg 58548829

00006130 L G L T N L H K I T S S G H Y E L R V D 00006189

<<<<<<<< | | | | | | | | | | | | | | | | | | | | <<<<<<<<

58548759 ttaggtctcaccaacctccataagataacaagttctggccattatgagctgcgtgtggac 58548700

00006190 L R D S G E S A Y A Q Y D K F V V A E P 00006249

<<<<<<<< | | | | | | | | | | | | | | | | | | | | <<<<<<<<

58548699 ctgagggacagcggcgagtcggcctacgctcagtacgataagttcgtggtggcagagccg 58548640

00006250 R T R Y K L H I G A Y S G T A 00006294

<<<<<<<< | | | | | | | | | | | | | | | <<<<<<<<

58548639 agaacgcgctataaactgcatatcggagcctacagtggaacagca 58548595

00006295 G D S M T Y H Q G R P F S T Y D N D N D 00006354

<<<<<<<< | | | | | | | | | | | | | | | | | | | | <<<<<<<<

58548266 ggtgactccatgacgtaccaccagggtcgacccttctccacctacgacaacgacaacgac 58548207

00006355 I A V T N C A L S Y K G A F W Y K N C H 00006414

<<<<<<<< | | | | | | | | | | | | | | | | | | | | <<<<<<<<

58548206 atcgccgtcaccaactgcgccctgtcctacaaaggcgccttctggtataaaaactgtcac 58548147

00006415 R V N L M G K Y G E K G H S K 00006459

<<<<<<<< | | | | | | | | | | | | | | | <<<<<<<<

58548146 cgtgtcaacctcatgggaaaatatggtgagaaaggtcacagcaag 58548102

00006460 G V N W F H W K G H E H S I E F A E M K 00006519

<<<<<<<< | | | | | | | | | | | | | | | | | | | | <<<<<<<<

58547870 ggggtcaactggtttcactggaagggccacgaacactcaattgaatttgcagagatgaaa 58547811

00006520 L R P A N F K N P E N R R K R S 00006567

<<<<<<<< | | | | | | | | | | | | | | | | <<<<<<<<

58547810 cttaggccggccaacttcaaaaacccggagaacagaaggaaacgatcg 58547763
