# Supplementary material for: Phylogenetic analysis of the tenascin gene family: evidence of origin early in the chordate lineage
Source: BMC Evol Biol. 2006 Aug 7;6:60. doi: 10.1186/1471-2148-6-60 (PMC1578592; doi:10.1186/1471-2148-6-60)
Supplement: Additional file 3 — Side-by-side alignment of predicted tenascins from Xenopus. [file 1471-2148-6-60-S3.doc]

Appendix 3: Side by side alignment of tenascins from Xenopus tropicalis.

Xenopus tenascin-C:

000001 M G S G H A D T L L S V A V A G R Q K G 000060

<<<<<< | | | | | | | | | | | | | | | | | | | | <<<<<<

243644 atgggttccggacacgcagatactttactgtctgtcgcggtggctgggcggcaaaaggga 243585

000061 V A L G H P I Q K S G P V N H T 000108

<<<<<< | | | | | | | | | | | | | | | | <<<<<<

243584 gtggccttggggcaccctattcagaaatccggccctgtcaatcataca 243537

000109 G V S L L E L S S S V L C M S G L F L M 000168

<<<<<< | | | | | | | | | | | | | | | | | | | | <<<<<<

242847 ggagtatccttgcttgaactcagctcaagtgtcttatgtatgtctgggttatttttaatg 242788

000169 P H L R K T K N 000192

<<<<<< | | | | | | | | <<<<<<

242787 ccccacttacgcaaaaccaagaat 242764

000193 P I A A L A Y T T F N H D P I D P S L L 000252

<<<<<< | | | | | | | | | | | | | | | | | | | | <<<<<<

240529 ccgattgcagcactagcctacaccaccttcaatcatgaccccatcgatccaagtcttctt 240470

000253 L K M L Y L G L P I K M Y C L L C T Y S 000312

<<<<<< | | | | | | | | | | | | | | | | | | | | <<<<<<

240469 ttgaaaatgctgtatttaggcttaccaattaaaatgtactgccttttgtgtacatactca 240410

000313 I F I N I T N I L M K 000345

<<<<<< | | | | | | | | | | | <<<<<<

240409 atattcataaatatcacaaatattctgatgaaa 240377

000346 V D L D P T S T K D N E L S P E V G K N 000405

<<<<<< | | | | | | | | | | | | | | | | | | | | <<<<<<

234543 gttgacttggatccaacaagcacaaaagataatgagctctctccagaagttggcaagaac 234484

000406 Y Q E H N L N G E N Q I V F T H R I N I 000465

<<<<<< | | | | | | | | | | | | | | | | | | | | <<<<<<

234483 taccaggagcataatttaaatggtgaaaatcaaatagtttttactcaccgtatcaacatt 234424

000466 P R R A C G C A S A P D I K D L L T R L 000525

<<<<<< | | | | | | | | | | | | | | | | | | | | <<<<<<

234423 cctcgaagagcatgtggctgtgcatcggcccctgatatcaaggaccttctgaccaggttg 234364

000526 E E L E G L V S S L R E Q C T T G A G C 000585

<<<<<< | | | | | | | | | | | | | | | | | | | | <<<<<<

234363 gaagagttagaaggactggtatcatctcttagagagcagtgcactacaggggctggatgc 234304

000586 C S S A Q 000600

<<<<<< | | | | | <<<<<<

234303 tgttctagtgcacaa 234289

000601 G V L E T K P Y C N G R G N Y S S E A K 000660

<<<<<< | | | | | | | | | | | | | | | | | | | | <<<<<<

232262 ggtgttctagaaaccaaaccttattgcaatggacgtggcaattacagttctgaagcaaaa 232203

000661 A C I C E P G W T G L N C T E I M C P G 000720

<<<<<< | | | | | | | | | | | | | | | | | | | | <<<<<<

232202 gcgtgtatttgcgaacctggatggactgggctcaactgcacagaaataatgtgtcctggg 232143

000721 Q C N N R G V C V N G A C V C S P G F F 000780

<<<<<< | | | | | | | | | | | | | | | | | | | | <<<<<<

232142 caatgcaataataggggtgtgtgtgtgaacggtgcatgtgtgtgtagcccaggtttcttt 232083

000781 G E D C S E V A C P D D C N D Q G K C V 000840

<<<<<< | | | | | | | | | | | | | | | | | | | | <<<<<<

232082 ggggaggactgtagtgaggtggcttgtccagatgactgcaatgaccagggtaaatgtgtc 232023

000841 N G R C V C F E G Y G G E D C K E E V C 000900

<<<<<< | | | | | | | | | | | | | | | | | | | | <<<<<<

232022 aatggccgttgtgtttgctttgagggctatggaggagaagactgcaaggaagaggtatgc 231963

000901 P L P C G E H G K C V N G Q C V C D E N 000960

<<<<<< | | | | | | | | | | | | | | | | | | | | <<<<<<

231962 ccattaccctgtggtgaacatggcaaatgtgtgaatggacaatgtgtgtgtgatgaaaat 231903

000961 F I G E D C S E P R C P N N C N N R G R 001020

<<<<<< | | | | | | | | | | | | | | | | | | | | <<<<<<

231902 tttataggggaagactgcagtgagcctcgctgcccaaataactgtaacaatcgtggacgt 231843

001021 C V D N E C V C D D P Y T G E D C S E L 001080

<<<<<< | | | | | | | | | | | | | | | | | | | | <<<<<<

231842 tgtgtggataatgagtgtgtctgtgatgatccctacacgggagaggactgcagtgaactt 231783

001081 I C P N D C F D R G R C V N G V C Y C E 001140

<<<<<< | | | | | | | | | | | | | | | | | | | | <<<<<<

231782 atatgccccaacgactgctttgatcgtggccgatgtgtgaatggagtatgctattgtgaa 231723

001141 E G F T G E D C G Q L A C P N N C N N H 001200

<<<<<< | | | | | | | | | | | | | | | | | | | | <<<<<<

231722 gaggggtttactggagaggactgtggtcaacttgcttgccccaataactgcaacaaccat 231663

001201 G R C V N G L C V C E T G Y T G D D C S 001260

<<<<<< | | | | | | | | | | | | | | | | | | | | <<<<<<

231662 ggccgctgtgtgaatggattatgtgtctgtgaaactggctatactggagatgactgcagt 231603

001261 E L A C P D N C N N R G R C I N G Q C V 001320

<<<<<< | | | | | | | | | | | | | | | | | | | | <<<<<<

231602 gaacttgcttgtccagacaactgcaataaccgcgggcgctgtataaatggtcagtgtgtt 231543

001321 C D E G Y T G E N C G E L R C P N D C N 001380

<<<<<< | | | | | | | | | | | | | | | | | | | | <<<<<<

231542 tgtgatgaaggttacacaggagaaaactgtggagaactaagatgtccaaatgattgtaac 231483

001381 N R G R C V N G Q C V C D D A Y I G S D 001440

<<<<<< | | | | | | | | | | | | | | | | | | | | <<<<<<

231482 aacagagggcgttgtgttaatgggcagtgtgtatgtgatgatgcctacataggatctgat 231423

001441 C S D L R C P N D C N N R G R C V N G Q 001500

<<<<<< | | | | | | | | | | | | | | | | | | | | <<<<<<

231422 tgttctgatcttagatgtcctaatgattgtaataacagaggccgctgtgttaatggacaa 231363

001501 C V C D E G F I G D D C G E L R C P D D 001560

<<<<<< | | | | | | | | | | | | | | | | | | | | <<<<<<

231362 tgtgtttgtgatgaaggattcataggggatgattgtggtgagcttaggtgccctgatgac 231303

001561 C N D R G R C V N G Q C V C D E G Y T G 001620

<<<<<< | | | | | | | | | | | | | | | | | | | | <<<<<<

231302 tgtaacgatagggggcgatgtgttaatggacaatgtgtgtgtgatgaaggatatacaggc 231243

001621 L D C G E L R C P N D C N N R G R C E N 001680

<<<<<< | | | | | | | | | | | | | | | | | | | | <<<<<<

231242 ttagactgtggggagcttaggtgtccaaatgactgtaataacaggggacgatgtgaaaat 231183

001681 G Q C V C D E E F T G E D C S E L R C P 001740

<<<<<< | | | | | | | | | | | | | | | | | | | | <<<<<<

231182 ggacagtgtgtgtgtgatgaagaatttacgggtgaagattgttctgaactgagatgcccc 231123

001741 N D C N N R G R C V N G Q C V C D T L F 001800

<<<<<< | | | | | | | | | | | | | | | | | | | | <<<<<<

231122 aatgactgtaacaacaggggtcgctgtgttaatggacagtgtgtgtgtgacactttattc 231063

001801 M G D D C G E L R C P D D C N N R G R C 001860

<<<<<< | | | | | | | | | | | | | | | | | | | | <<<<<<

231062 atgggtgatgactgtggtgaactgcgctgccctgatgactgcaataacagaggacgctgt 231003

001861 I D G Q C V C D E G F T G D N C A E L T 001920

<<<<<< | | | | | | | | | | | | | | | | | | | | <<<<<<

231002 attgatgggcaatgtgtgtgcgatgaaggatttacaggggacaactgtgctgaactaacc 230943

001921 C L D N C H N Q G R C I D G Q C V C D E 001980

<<<<<< | | | | | | | | | | | | | | | | | | | | <<<<<<

230942 tgccttgataattgccataaccaaggccgttgcattgatggacagtgtgtctgtgatgaa 230883

001981 G F T G D F C S E 002007

<<<<<< | | | | | | | | | <<<<<<

230882 ggtttcactggagacttctgctcagaa 230856

002008 V S P P K D L K V I D V T T K T M N L E 002067

<<<<<< | | | | | | | | | | | | | | | | | | | | <<<<<<

229830 gtttctccaccaaaggacttaaaggtgattgatgtgacgacaaaaaccatgaatcttgaa 229771

002068 W Q N K M R V N E Y L V S Y V P T S P G 002127

<<<<<< | | | | | | | | | | | | | | | | | | | | <<<<<<

229770 tggcaaaacaaaatgcgagtcaatgaatatcttgtttcatatgttcctaccagccctgga 229711

002128 G L E L D F R V P G D Q T A A T I R E L 002187

<<<<<< | | | | | | | | | | | | | | | | | | | | <<<<<<

229710 gggttggagcttgattttagagttccaggggaccaaacagctgccactattagagaactt 229651

002188 E P G V E Y F V R V Y A I L R N Q K S I 002247

<<<<<< | | | | | | | | | | | | | | | | | | | | <<<<<<

229650 gaacctggggtggagtattttgtacgtgtatatgcaatcctcagaaatcagaagagtatt 229591

002248 P V S A R V S T 002271

<<<<<< | | | | | | | | <<<<<<

229590 cctgtgagcgcaagggtatcgact 229567

002275 L P T T D D L R F K S V R E T F V D V E 002334

<<<<<< | | | | | | | | | | | | | | | | | | | | <<<<<<

228274 ctcccaactacagatgacttaaggtttaaatctgtgagagaaacatttgttgatgtggaa 228215

002335 W D P L D I S F D T W Q L I F R N T 002388

<<<<<< | | | | | | | | | | | | | | | | | | <<<<<<

228214 tgggatccactggacatttcttttgatacttggcaactcatcttcaggaacaca 228161

002389 K E E N G E I T T S L E R P V T S F V Q 002448

<<<<<< | | | | | | | | | | | | | | | | | | | | <<<<<<

227675 aaagaggagaatggcgagataaccaccagcttagaaagacctgtcacatcttttgtgcaa 227616

002449 T G L A P G E T Y E V S I Q V V K N K T 002508

<<<<<< | | | | | | | | | | | | | | | | | | | | <<<<<<

227615 actggacttgcaccaggggagacctatgaagtgtctatacaagttgttaaaaacaaaacc 227556

002509 Q G P P L T K V T T T 002541

<<<<<< | | | | | | | | | | | <<<<<<

227555 caagggccacccttaactaaagtgaccaccaca 227523

002545 L D A P S Q V E V N D V T D T S A L I T 002604

<<<<<< | | | | | | | | | | | | | | | | | | | | <<<<<<

224995 ttggatgcaccaagtcaggttgaagtaaatgatgttactgatacttctgctttaataacc 224936

002605 W V K P L A E I D G I S L S Y G T E N E 002664

<<<<<< | | | | | | | | | | | | | | | | | | | | <<<<<<

224935 tgggttaagcccctggcagaaatcgatggtatttcgttgtcttatggaactgagaatgag 224876

002665 P I T T V E L T E D E T Q Y S M N G L R 002724

<<<<<< | | | | | | | | | | | | | | | | | | | | <<<<<<

224875 ccaataaccactgtggaactgactgaggatgaaacacagtattcaatgaatggtttacga 224816

002725 P D T E Y E V T L I S R R R E M T S S P 002784

<<<<<< | | | | | | | | | | | | | | | | | | | | <<<<<<

224815 ccggacacagaatatgaagtaacacttatctctcgacgcagagaaatgacaagttcccca 224756

002785 A T E T F T T 002805

<<<<<< | | | | | | | <<<<<<

224755 gcaaccgagactttcacaaca 224735

002806 E N I P E V G D L A V S D V T T N S F D 002865

<<<<<< | | | | | | | | | | | | | | | | | | | | <<<<<<

216982 gaaaacattccagaagttggtgatttagctgtctctgatgtcactacaaacagctttgat 216923

002866 L S W K A T S D S F E E F V I E V I D T 002925

<<<<<< | | | | | | | | | | | | | | | | | | | | <<<<<<

216922 ctgtcatggaaagcaacctcggattcatttgaagaatttgttattgaagtgattgatacc 216863

002926 N R L F E P I T L N V S G N L R T S S V 002985

<<<<<< | | | | | | | | | | | | | | | | | | | | <<<<<<

216862 aacaggttatttgaaccaataacactgaatgtctcaggcaacctcagaacatcatctgtc 216803

002986 S G L L P K T D Y S I S L F G I V H G L 003045

<<<<<< | | | | | | | | | | | | | | | | | | | | <<<<<<

216802 tctggactgttaccaaagacagattacagcatcagcctctttggcattgtccatggtctg 216743

003046 R T Q A I S T S A T T 003078

<<<<<< | | | | | | | | | | | <<<<<<

216742 cgcacacaggccataagtacttctgctaccaca 216710

003079 E P E P E V G N L L V S K I T S D S F H 003138

<<<<<< | | | | | | | | | | | | | | | | | | | | <<<<<<

214819 gagccagagcctgaggttggcaatcttctagtttccaaaataacttcagacagtttccac 214760

003139 L S W N A D E S G F D N F I L K I R D A 003198

<<<<<< | | | | | | | | | | | | | | | | | | | | <<<<<<

214759 ctatcatggaacgctgatgagagtggttttgacaattttatacttaaaataagggatgcc 214700

003199 K Q Q F E P I T L T V P G S E R S S L V 003258

<<<<<< | | | | | | | | | | | | | | | | | | | | <<<<<<

214699 aaacagcagtttgagcctataactctaactgtccctggcagtgaacgttcttcattggta 214640

003259 T G L R D G T E Y D I E L V G V A S G Q 003318

<<<<<< | | | | | | | | | | | | | | | | | | | | <<<<<<

214639 acaggattgagagatggtacagagtatgacattgaactggtcggcgttgccagtggtcaa 214580

003319 R S P P I K G M A T T 003351

<<<<<< | | | | | | | | | | | <<<<<<

214579 cgctctcctcccataaaaggaatggcaactaca 214547

003352 A L G S P K G L S F S D I T E N S A K V 003411

<<<<<< | | | | | | | | | | | | | | | | | | | | <<<<<<

209373 gctctaggctctcctaaaggactttcattctctgatataactgaaaacagtgctaaagtt 209314

003412 T W V A P R I R V E K F L I T Y I P V S 003471

<<<<<< | | | | | | | | | | | | | | | | | | | | <<<<<<

209313 acctgggtagcaccaaggatcagggtggaaaaattcctgatcacatatattcctgtgtct 209254

003472 G 003474

<<<<<< | <<<<<<

209253 gga 209251

003475 G S P N T V E V D G S K T Q S T L V N L 003534

<<<<<< | | | | | | | | | | | | | | | | | | | | <<<<<<

207880 ggttctcctaacactgtggaagtagatgggtctaaaactcagtcaactttggtaaatctc 207821

003535 H P G V E Y I V S I V S V Q G S E E S E 003594

<<<<<< | | | | | | | | | | | | | | | | | | | | <<<<<<

207820 catccaggtgtagaatatattgtgtctattgtatctgtgcaaggaagcgaagaaagtgag 207761

003595 P A S G T F T T 003618

<<<<<< | | | | | | | | <<<<<<

207760 ccagcttctgggactttcaccaca 207737

003619 A L D S P S G L K V V N I T E S D A L A 003678

<<<<<< | | | | | | | | | | | | | | | | | | | | <<<<<<

205604 gctctggacagtccatccggtcttaaggttgtaaacattacagagtcagatgcactggcc 205545

003679 L W Q P S L A S V D N Y V F S Y S A K N 003738

<<<<<< | | | | | | | | | | | | | | | | | | | | <<<<<<

205544 ctctggcaaccatctttggcatctgtggacaattatgtcttctcctactctgcaaagaat 205485

003739 A P A V T H S I S G N T V E N D L I G L 003798

<<<<<< | | | | | | | | | | | | | | | | | | | | <<<<<<

205401 gcacctgcagtaacacattccatctcaggcaatactgtagagaatgatttgattggtctg 205342

003799 H P S T E Y T V K V Y A V R G Q H R S A 003858

<<<<<< | | | | | | | | | | | | | | | | | | | | <<<<<<

205341 catccctctaccgaatatactgttaaagtctatgctgtcagaggccaacatcggagtgct 205282

003859 E I S T K F T T G M P R I P I E T L T K 003918

<<<<<< | | | | | | | | | | | | | | | | | | | | <<<<<<

205281 gaaatctctacaaaattcaccacaggtatgccacggataccaatagaaaccttgaccaaa 205222

003919 C I D Q Q V K F K L K V A I H C K I R S 003978

<<<<<< | | | | | | | | | | | | | | | | | | | | <<<<<<

205221 tgcattgaccaacaagtcaaattcaaacttaaggtggccatacattgtaagatccgctca 205162

003979 F G E V T K R A E L L L I C P S M 004029

<<<<<< | | | | | | | | | | | | | | | | | <<<<<<

205161 tttggtgaagtcaccaagcgagcagagcttctcctgatatgcccatctatg 205111

004030 A L D G P R D L S A S E I Q S E S A L L 004089

<<<<<< | | | | | | | | | | | | | | | | | | | | <<<<<<

202864 gcccttgatggtccaagagatttgagtgcaagtgagatccagtcagaatcagcattgctc 202805

004090 T W K P P R S T I T G Y V L I Y E S V D 004149

<<<<<< | | | | | | | | | | | | | | | | | | | | <<<<<<

202804 acctggaaacctccaaggtcaacaattaccggatatgtgctaatctacgagtctgtggat 202745

004150 G T V K 004161

<<<<<< | | | | <<<<<<

202744 ggaaccgtcaag 202733

004162 E V V V G P D T T S Y R L L D L S P S A 004221

<<<<<< | | | | | | | | | | | | | | | | | | | | <<<<<<

201155 gaagttgttgtgggtccagataccacttcttacagactactcgatctaagcccatctgct 201096

004222 Q Y I A R V Q A I N G D R R S K E I Q T 004281

<<<<<< | | | | | | | | | | | | | | | | | | | | <<<<<<

201095 caatatattgcaagggttcaagcgataaatggtgacagaagaagcaaggagatccaaacc 201036

004282 I F I 004290

<<<<<< | | | <<<<<<

201035 atttttata 201027

004291 T I G L L Y P Y P K D C S Q A L L N G E 004350

<<<<<< | V | | | | | | | | | | | | | | | | | | <<<<<<

197872 acagttgggctactttatccatatccaaaggactgttcccaggctttgctgaatggagaa 197813

004351 A D S G L Y T I Y V N G D Q S Q P M E V 004410

<<<<<< | | | | | | | | | | | | | | | | | | | | <<<<<<

197812 gcagactctggtttatacacaatttatgtaaatggtgaccagtctcagcccatggaggta 197753

004411 Y C D M S V D G G G W I 004446

<<<<<< | | | | | | | | | | | | <<<<<<

197752 tactgcgacatgagtgttgatggaggaggatggatt 197717

004447 V F L R R T D G S E E F Y R N W K T Y S 004506

<<<<<< | | | | | | | | | | | | | | | | | | | | <<<<<<

197057 gtatttttacggcgtacagatgggtcagaagaattttacagaaactggaagacatacagt 196998

004507 A G F G N I N N E F F M 004542

<<<<<< | | | | | | | | | | | | <<<<<<

196997 gcaggatttggaaatattaacaatgaatttttcatg 196962

004543 G L E N L H K L T S L G Q Y E L R V D L 004602

<<<<<< | | | | | | | | | | | | | | | | | | | | <<<<<<

195891 ggtctggaaaacctgcataaactgacctccctgggtcagtatgagctacgagtagacctt 195832

004603 R D N D E T A Y A V Y D K F S V G D A K 004662

<<<<<< | | | | | | | | | | | | | | | | | | | | <<<<<<

195831 cgtgataatgatgaaactgcatatgcagtctatgataagtttagcgttggagatgcaaag 195772

004663 S R F R L K V E G Y S G T A 004704

<<<<<< | | | | | | | | | | | | | | <<<<<<

195771 agtcgcttccggctcaaggttgaaggttacagtggtacagca 195730

004705 G D S M T Y H N G R S F S T F D K D N D 004764

<<<<<< | | | | | | | | | | | | | | | | | | | | <<<<<<

194404 ggagattccatgacttaccacaatgggagatcattttccacatttgacaaggacaatgat 194345

004765 S A I T N C A L S Y K G A F W Y K N C H 004824

<<<<<< | | | | | | | | | | | | | | | | | | | | <<<<<<

194344 tcagctattacaaactgcgctttgtcatacaaaggggctttctggtacaagaactgtcac 194285

004825 R V N L M G R Y G D T N H S 004866

<<<<<< | | | | | | | | | | | | | | <<<<<<

194284 agagtaaaccttatgggtagatacggtgacacaaaccacagt 194243

004867 Q G V N W F H W K G H E Y S I Q F A E M 004926

<<<<<< | | | | | | | | | | | | | | | | | | | | <<<<<<

192763 cagggtgtcaactggttccactggaagggacatgaatactcaattcagtttgcagaaatg 192704

004927 K I R P V S F R N L E G R R K R A 004977

<<<<<< | | | | | | | | | | | | | | | | | <<<<<<

192703 aagataagaccagtaagcttcagaaatcttgaaggaaggcggaagagagca 192653

Xenopus tenascin-W:

0000001 M D I I M S F W A V L V F L L C A P P Y 0000060

>>>>>>> | | | | | | | | | | | | | | | | | | | | >>>>>>>

1653126 atggacattattatgtctttctgggcggttttggtgtttctcttgtgtgcccccccttat 1653185

0000061 L L T S A A A I K T T Q I D N C S S E K 0000120

>>>>>>> | | | | | | | | | | | | | | | | | | | | >>>>>>>

1653186 ctgctgacttctgctgctgctataaaaactacccaaattgataattgtagcagtgagaaa 1653245

0000121 G L T F T H V Y S S P G E E N E K L Q T 0000180

>>>>>>> | | | | | | | | | | | | | | | | | | | | >>>>>>>

1653246 ggcctcacatttacccatgtgtacagcagccctggcgaggagaatgagaagcttcaaaca 1653305

0000181 V H S D E Q N L V F R H Y I H L Q T P A 0000240

>>>>>>> | | | | | | | | | | | | | | | | | | | | >>>>>>>

1653306 gtgcattcagatgagcagaatcttgtctttaggcattatatccatctccaaacccctgca 1653365

0000241 A D C E K N K L L N D L L S R L Q A L E 0000300

>>>>>>> | | | | | | | | | | | | | | | | | | | | >>>>>>>

1653366 gcagactgtgagaaaaacaagctacttaatgacctgctgtccagactacaagctttagaa 1653425

0000301 V E M K E L K E K C D G C C G G 0000348

>>>>>>> | | | | | | | | | | | | | | | | >>>>>>>

1653426 gtggagatgaaagaattaaaggagaagtgtgatgggtgctgtggagga 1653473

0000349 S G A C H N C N G H G R F L P Q L G H C 0000408

>>>>>>> | | | | | | | | | | | | | | | | | | | | >>>>>>>

1656253 tcaggggcctgccataactgcaatggacatggaaggtttcttccacaattgggtcattgc 1656312

0000409 Q C D E G W E G D D C S T K S C P N N C 0000468

>>>>>>> | | | | | | | | | | | | | | | | | | | | >>>>>>>

1656313 cagtgtgatgaaggctgggagggggatgactgttctacaaagagttgccctaacaactgt 1656372

0000469 A G N G K C I D G V C Q C A P G Y S G P 0000528

>>>>>>> | | | | | | | | | | | | | | | | | | | | >>>>>>>

1656373 gcagggaatgggaaatgtattgatggtgtttgccagtgcgcaccaggctatagtggtcca 1656432

0000529 D C S E K V C P F D C G K Y G T C E D G 0000588

>>>>>>> | | | | | | | | | | | | | | | | | | | | >>>>>>>

1656433 gactgcagtgagaaggtgtgtccctttgattgtggtaagtatggaacatgtgaagatggc 1656492

0000589 S C K C A E G Y T G P A C R K K K C P F 0000648

>>>>>>> | | | | | | | | | | | | | | | | | | | | >>>>>>>

1656493 tcctgtaaatgtgcagaagggtacactggcccagcatgtagaaagaaaaaatgtcccttc 1656552

0000649 D C G E F G T C V D G T C Q C S E G Y T 0000708

>>>>>>> | | | | | | | | | | | | | | | | | | | | >>>>>>>

1656553 gactgtggtgaatttgggacttgtgttgatggtacctgccagtgctcagaggggtacact 1656612

0000709 G P A C R K K K C P L D C G E H G K C I 0000768

>>>>>>> | | | | | | | | | | | | | | | | | | | | >>>>>>>

1656613 gggccagcatgcagaaaaaagaagtgtcctttagactgtggtgaacatggaaagtgtatt 1656672

0000769 D G S C K C S E G Y K G V D C K E K K C 0000828

>>>>>>> | | | | | | | | | | | | | | | | | | | | >>>>>>>

1656673 gatggatcttgcaaatgttcagagggatacaagggcgtggactgcaaagaaaagaagtgt 1656732

0000829 A V D C G P N G R C V D G Q C V C N D G 0000888

>>>>>>> | | | | | | | | | | | | | | | | | | | | >>>>>>>

1656733 gctgttgactgtggccccaatggacgatgtgttgatggacaatgtgtttgcaacgatggt 1656792

0000889 F M G S S C S I 0000912

>>>>>>> | | | | | | | | >>>>>>>

1656793 ttcatgggaagcagttgttcaata 1656816

0000913 E I A S L C S G H G K Y L M D T G G C Q 0000972

>>>>>>> | | | | | | | | | | | | | | | | | | | | >>>>>>>

1659126 gaaatcgctagtctctgcagtggacacggcaagtacctaatggacactggtggttgtcag 1659185

0000973 C D D G W E G T D C S Q R S C P N N C D 0001032

>>>>>>> | | | | | | | | | | | | | | | | | | | | >>>>>>>

1659186 tgtgatgatggctgggagggaactgactgttcacagagaagttgtccaaataattgtgac 1659245

0001033 N N G V C V D G V C Q C F S G Y T G L D 0001092

>>>>>>> | | | | | | | | | | | | | | | | | | | | >>>>>>>

1659246 aataatggggtatgtgttgatggagtatgccagtgcttcagtggttacactggcctagac 1659305

0001093 C S E R E C P F D C G E H G S C V D G A 0001152

>>>>>>> | | | | | | | | | | | | | | | | | | | | >>>>>>>

1659306 tgcagtgagagggagtgtccatttgattgtggagagcatggcagttgtgttgatggagct 1659365

0001153 C K C S V G Y T G L T C R E E D C L V N 0001212

>>>>>>> | | | | | | | | | | | | | | | | | | | | >>>>>>>

1659366 tgcaagtgctcagttggatacaccgggctgacttgcagagaagaagactgccttgtgaat 1659425

0001213 C G E N G R C D G G Q C F C E E G F I G 0001272

>>>>>>> | | | | | | | | | | | | | | | | | | | | >>>>>>>

1659426 tgtggtgaaaatgggcgctgcgatggtggccagtgcttctgcgaggagggatttattggg 1659485

0001273 E D C S E 0001287

>>>>>>> | | | | | >>>>>>>

1659486 gaagattgttctgaa 1659500

0001288 V I T V Q N L H L I S A T E D S L S I A 0001347

>>>>>>> | | | | | | | | | | | | | | | | | | | | >>>>>>>

1659879 gtgattacagtgcagaatttacatttgatcagtgctacagaggattcactgagcatcgct 1659938

0001348 W D M L L E V D Y Y L L V Y Y P L D N E 0001407

>>>>>>> | | | | | | | | | | | | | | | | | | | | >>>>>>>

1659939 tgggatatgttgcttgaagtggattattacttgcttgtatattaccccttggacaatgaa 1659998

0001408 G L K K E I Q V S V D K D T Y Q I M G L 0001467

>>>>>>> | | | | | | | | | | | | | | | | | | | | >>>>>>>

1659999 ggcttaaaaaaggaaatacaagtatctgtagacaaggatacataccaaatcatgggtttg 1660058

0001468 N P G T K Y K V L M Y N A K N G V T S Q 0001527

>>>>>>> | | | | | | | | | | | | | | | | | | | | >>>>>>>

1660059 aatcctggtaccaaatataaggtgctcatgtacaatgcaaagaatggagtgaccagccag 1660118

0001528 P A E L L A K T 0001551

>>>>>>> | | | | | | | | >>>>>>>

1660119 ccagcagaactgcttgccaaaaca 1660142

0001552 D S I S L G T L W V A E E T E D S L E V 0001611

>>>>>>> | | | | | | | | | | | | | | | | | | | | >>>>>>>

1661716 gatagtatttcactggggactctgtgggttgcagaagagacagaagactctttggaagta 1661775

0001612 E W E N P I V N V D H Y K L K Y A Q Q L 0001671

>>>>>>> | | | | | | | | | | | | | | | | | | | | >>>>>>>

1661776 gaatgggaaaatcccattgtaaatgtggatcattacaaactgaagtatgctcagcagctg 1661835

0001672 N G A E R E V I V S Q S Q D P K S R Y T 0001731

>>>>>>> | | | | | | | | | | | | | | | | | | | | >>>>>>>

1661836 aatggggctgagagagaagttatagtgtcacaaagccaagaccctaagagcaggtacacc 1661895

0001732 I T 0001737

>>>>>>> | | >>>>>>>

1661896 atcaca 1661901

0001738 G L Q P S T S Y Q I S V Q T V R S G L E 0001797

>>>>>>> | | | | | | | | | | | | | | | | | | | | >>>>>>>

1662369 ggtctgcagcctagcacatcctatcagataagtgtccagactgtcagaagtggtctagag 1662428

0001798 G K P S T A V G V T 0001827

>>>>>>> | | | | | | | | | | >>>>>>>

1662429 ggaaagccatctactgcagttggggttaca 1662458

0001828 G I D G P K N L V T T E V G E D T A S L 0001887

>>>>>>> | | | | | | | | | | | | | | | | | | | | >>>>>>>

1664720 gggattgatggtcctaaaaatttagtgaccacagaggtgggtgaggatacagccagcctg 1664779

0001888 S W K G S Q A D I D K Y M L R Y S A H D 0001947

>>>>>>> | | | | | | | | | | | | | | | | | | | | >>>>>>>

1664780 tcctggaaaggatcccaagcagacatagataaatatatgctgagatattcggcacatgat 1664839

0001948 G I A E E I S L G K E K I S T S L S N L 0002007

>>>>>>> | | | | | | | | | | | | | | | | | | | | >>>>>>>

1664840 gggatcgctgaagagatatccttgggcaaagagaagatttcaacatctctgagtaaccta 1664899

0002008 T P G T E Y M F H L W A E K G T Q Q S K 0002067

>>>>>>> | | | | | | | | | | | | | | | | | | | | >>>>>>>

1664900 acgcctggcacagaatacatgtttcatctgtgggcagaaaaaggcacccaacaaagcaag 1664959

0002068 R T S V T A V T 0002091

>>>>>>> | | | | | | | | >>>>>>>

1664960 agaaccagtgtcacagcagtgact 1664983

0002092 E I D P P K N L Q Y S K S Q P Q A S L T 0002151

>>>>>>> | | | | | | | | | | | | | | | | | | | | >>>>>>>

1671012 gagatcgacccaccaaagaatctgcaatattctaaatcacaaccgcaagcctctcttacc 1671071

0002152 W N Q P I A Q I D G Y V L V L E D S D G 0002211

>>>>>>> | | | | | | | | | | | | | | | | | | | | >>>>>>>

1671072 tggaatcaacctattgcacaaattgatggctatgttttagtcttggaagactctgatgga 1671131

0002212 G Q 0002217

>>>>>>> | | >>>>>>>

1671132 ggccag 1671137

0002218 Q E I Q L D S T V N N F E L Q D L N K G 0002277

>>>>>>> | | | | | | | | | | | | | | | | | | | | >>>>>>>

1672084 caggagattcagctggattctacagtaaataattttgaactacaagacctgaataaaggg 1672143

0002278 L K Y T V Y L L A Y R G D R R S R Q V T 0002337

>>>>>>> | | | | | | | | | | | | | | | | | | | | >>>>>>>

1672144 ctaaagtacacagtctacctgctagcttatagaggagaccgtcggagcagacaagtcacc 1672203

0002338 T S F Y T 0002352

>>>>>>> | | | | | >>>>>>>

1672204 accagcttttataca 1672218

0002356 I D P P T N L Q S F D V T Q T E A S L T 0002415

>>>>>>> | | | | | | | | | | | | | | | | | | | | >>>>>>>

1674638 attgaccccccaacaaacctgcaatcttttgatgttacgcagactgaagcctccctcacg 1674697

0002416 W T P P R A K I D G Y I L T Y T D A D G 0002475

>>>>>>> | | | | | | | | | | | | | | | | | | | | >>>>>>>

1674698 tggactccaccaagggcaaagatagatggctacatactcacttatacggatgcagatggc 1674757

0002476 S T E 0002484

>>>>>>> | | | >>>>>>>

1674758 agcacagag 1674766

0002485 E I Q L D S S S K S F A M K N L K K G L 0002544

>>>>>>> | | | | | | | | | | | | | | | | | | | | >>>>>>>

1677506 gagattcagctggattcttcaagtaaaagttttgccatgaaaaacttgaagaaaggatta 1677565

0002545 K Y T V Y L T A F K G D S R S S Q A S T 0002604

>>>>>>> | | | | | | | | | | | | | | | | | | | | >>>>>>>

1677566 aagtacacagtttacttgacagctttcaaaggagacagtaggagcagccaggccagtacc 1677625

0002605 I F S T 0002616

>>>>>>> | | | | >>>>>>>

1677626 atattcagtaca 1677637

0002617 V A F I I T H P S D C I Q I Q L S G N R 0002676

>>>>>>> | | | | | | | | | | | | | | | | | | | | >>>>>>>

1678828 gttgcctttattatcacccacccatctgactgcattcaaatccaactaagtggaaacaga 1678887

0002677 Q S G V Y T I Y P G G D T A K G V R V Y 0002736

>>>>>>> | | | | | | | | | | | | | | | | | | | | >>>>>>>

1678888 caaagtggtgtatacactatctacccaggtggtgatacggcaaaaggagtcagagtttat 1678947

0002737 C D Q E T D G G G W I 0002769

>>>>>>> | | | | | | | | | | | >>>>>>>

1678948 tgtgatcaagagactgatggtggaggatggatt 1678980

0002770 V F Q R R N S G K L D F Y Q R W R T Y V 0002829

>>>>>>> | | | | | | | | | | | | | | | | | | | | >>>>>>>

1679376 gtctttcagagaagaaacagtggaaagcttgacttttaccagcgatggagaacttatgtg 1679435

0002830 E G F G D P S D E F W L 0002865

>>>>>>> | | | | | | | | | | | | >>>>>>>

1679436 gagggatttggtgatccaagtgatgaattctggctt 1679471

0002866 G L E W I H K L T S S P G N N Y E I R V 0002925

>>>>>>> | | | | | | | | | | | | | | | | | | | | >>>>>>>

1680053 ggtcttgagtggatacataagctgacttcttctccaggcaataactatgaaatacgtgta 1680112

0002926 D L R A G D E S V Y A F Y R N F R V G S 0002985

>>>>>>> | | | | | | | | | | | | | | | | | | | | >>>>>>>

1680113 gatctgcgtgctggggatgagtcggtctatgctttttaccgcaatttccgggtgggctct 1680172

0002986 S K D R Y K L S I S D Y S G T 0003030

>>>>>>> | | | | | | | | | | | | | | | >>>>>>>

1680173 tcaaaagacaggtataaactgtccatcagtgactactctggcact 1680217

0003031 A G D G L T Y H N G W K F S T W D K D N 0003090

>>>>>>> | | | | | | | | | | | | | | | | | | | | >>>>>>>

1681961 gcaggtgatggacttacatatcacaacggctggaagttcagtacatgggacaaagataat 1682020

0003091 D I A L T N C A L S H R G A F W Y K N C 0003150

>>>>>>> | | | | | | | | | | | | | | | | | | | | >>>>>>>

1682021 gacattgcacttactaactgtgccctctcacatcgtggagccttttggtataaaaactgt 1682080

0003151 H L A N L N G Q Y G E T G H S Q 0003198

>>>>>>> | | | | | | | | | | | | | | | | >>>>>>>

1682081 caccttgccaacctgaatgggcagtatggagaaactggtcacagccag 1682128

0003199 G V N W E P W K G H E F S V P F V E M K 0003258

>>>>>>> | | | | | | | | | | | | | | | | | | | | >>>>>>>

1683308 ggtgtaaattgggagccatggaaaggacatgagttttctgtcccctttgttgaaatgaag 1683367

0003259 M R P N L K S 0003279

>>>>>>> | | | | | | | >>>>>>>

1683368 atgcgtcccaatctaaagtcc 1683388

Xenopus tenascin-X:

000001 M P S F L P L L F F I L F F K G C L A P 000060

>>>>>> | | | | | | | | | | | | | | | | | | | | >>>>>>

102616 atgccctctttcctgcctctgctcttcttcattttgtttttcaagggttgtctggcccct 102675

000061 P S T S T Q C P D A G G V R A L L K R L 000120

>>>>>> | | | | | | | | | | | | | | | | | | | | >>>>>>

102676 ccatccacatcaactcaatgtccagatgcaggaggggtgcgagctctcctcaagcgactt 102735

000121 E I L E K L V R D I K G Q C S P P C C G 000180

>>>>>> | | | | | | | | | | | | | | | | | | | | >>>>>>

102736 gaaattttggaaaagttggttcgggacataaaaggacagtgttcacctccatgttgtgga 102795

000181 N V Q S G A 000198

>>>>>> | | | | | | >>>>>>

102796 aatgtgcagagtggagct 102813

000199 D D P A S H K S P S C P Q P A E S C S G 000258

>>>>>> | | | | | | | | | | | | | | | | | | | | >>>>>>

106282 gatgaccctgcaagtcacaaatctccttcatgtccacagccagctgaatcatgctctggg 106341

000259 G C G G E E H G I C I D G Q C Q C K D G 000318

>>>>>> | | | | | | | | | | | | | | | | | | | | >>>>>>

106342 ggttgtggaggggaagaacatggaatctgcattgatggacagtgtcagtgcaaagatgga 106401

000319 Y M G E N C Q L K T C P E D C N D Q G R 000378

>>>>>> | | | | | | | | | | | | | | | | | | | | >>>>>>

106402 tacatgggagaaaactgccagctgaaaacttgtcctgaagattgtaatgaccaaggtcga 106461

000379 C K D G Q C F C F S G Y F G V D C S S K 000438

>>>>>> | | | | | | | | | | | | | | | | | | | | >>>>>>

106462 tgcaaagatggccaatgcttctgcttttctggatactttggagttgattgtagctcaaag 106521

000439 S C P N N C Q N H G R C D K G V C I C D 000498

>>>>>> | | | | | | | | | | | | | | | | | | | | >>>>>>

106522 tcctgtcctaataattgtcaaaatcatggacgctgtgataaaggagtttgtatttgtgac 106581

000499 P G F T G V D C S S R T C P K N C F N R 000558

>>>>>> | | | | | | | | | | | | | | | | | | | | >>>>>>

106582 cctggttttactggagttgactgtagttccagaacctgccccaaaaactgtttcaataga 106641

000559 G R C E D G V C I C Y P D Y T G P D C S 000618

>>>>>> | | | | | | | | | | | | | | | | | | | | >>>>>>

106642 ggtcgatgtgaagatggagtatgtatatgttaccctgattataccggtcctgattgcagc 106701

000619 I K T C L N D C Q D H G R C E D G M C V 000678

>>>>>> | | | | | | | | | | | | | | | | | | | | >>>>>>

106702 attaaaacttgccttaacgactgtcaagaccatgggcgttgtgaagatggaatgtgtgtg 106761

000679 C D P G F T G I D C S S R T C H N D C Q 000738

>>>>>> | | | | | | | | | | | | | | | | | | | | >>>>>>

106762 tgtgatcctggattcactggaatagattgtagttccagaacttgccacaatgactgtcag 106821

000739 N H G R C E N G L C V C D S G Y S G P D 000798

>>>>>> | | | | | | | | | | | | | | | | | | | | >>>>>>

106822 aatcatggcagatgtgaaaatggactttgtgtttgtgattctgggtattctggaccagat 106881

000799 C G I M S C P E D C N E Q G R C V S G V 000858

>>>>>> | | | | | | | | | | | | | | | | | | | | >>>>>>

106882 tgtggaattatgagctgcccagaagattgcaatgagcagggccgctgtgtgtctggagtc 106941

000859 C V C D S G F I G P D C G T R V C S P E 000918

>>>>>> | | | | | | | | | | | | | | | | | | | | >>>>>>

106942 tgtgtatgtgatagtggttttattgggccagattgtggaactagggtctgttctcccgaa 107001

000919 C E R R G R C E D G E C I C N P G F T G 000978

>>>>>> | | | | | | | | | | | | | | | | | | | | >>>>>>

107002 tgtgagagacgtggtcgttgtgaggatggtgagtgtatctgtaaccctggatttactggc 107061

000979 P D C E I K T C P N D C H K Q G M C V D 001038

>>>>>> | | | | | | | | | | | | | | | | | | | | >>>>>>

107062 ccagactgtgagataaaaacttgccctaatgattgtcacaaacaaggaatgtgtgtggat 107121

001039 G K C V C D S G Y T G V D C Q V K T C P 001098

>>>>>> | | | | | | | | | | | | | | | | | | | | >>>>>>

107122 ggaaaatgtgtttgtgattctggatatacaggagtagactgccaagttaaaacttgccca 107181

001099 N K C H N R G R C E D G I C I C N S G Y 001158

>>>>>> | | | | | | | | | | | | | | | | | | | | >>>>>>

107182 aacaaatgtcacaaccggggtaggtgtgaagatggaatctgcatttgtaattctggatat 107241

001159 S G S D C G S K S C P K N C S G N G Q C 001218

>>>>>> | | | | | | | | | | | | | | | | | | | | >>>>>>

107242 tctgggtcagattgtgggtcaaaatcttgtccaaagaactgcagtgggaatggccaatgt 107301

001219 V K G K C V C D S G F I G P V C G T R A 001278

>>>>>> | | | | | | | | | | | | | | | | | | | | >>>>>>

107302 gtgaagggcaagtgtgtatgtgattctggatttattggcccagtttgtgggaccagagct 107361

001279 C P A G C G N H G R C L R G T C V C S P 001338

>>>>>> | | | | | | | | | | | | | | | | | | | | >>>>>>

107362 tgtccagctgggtgtggcaatcatggacgctgtttgagagggacctgtgtctgctctcca 107421

001339 G Y T G V D C A S R L C P K N C H N R G 001398

>>>>>> | | | | | | | | | | | | | | | | | | | | >>>>>>

107422 gggtacactggggtagattgtgcatctaggctatgccccaaaaactgccataaccgaggc 107481

001399 R C E Q G V C I C N P E Y I G L D C G S 001458

>>>>>> | | | | | | | | | | | | | | | | | | | | >>>>>>

107482 aggtgtgaacagggagtctgtatatgtaatcctgaatatataggccttgattgtggatct 107541

001459 R T C P K N C H G K G Q C D D G V C I C 001518

>>>>>> | | | | | | | | | | | | | | | | | | | | >>>>>>

107542 aggacttgccccaaaaactgccacggtaaggggcaatgtgatgatggtgtgtgtatttgt 107601

001519 D L G Y T G L D C A T K S C F N D C H H 001578

>>>>>> | | | | | | | | | | | | | | | | | | | | >>>>>>

107602 gacttgggatatactggcttggattgtgcaactaaatcctgcttcaatgactgccaccat 107661

001579 R G R C E D G V C I C D V G Y T G L D C 001638

>>>>>> | | | | | | | | | | | | | | | | | | | | >>>>>>

107662 agggggcgttgtgaagatggtgtgtgtatttgtgatgttggatatacaggcctagattgt 107721

001639 G T L S C P K D C H N R 001674

>>>>>> | | | | | | | | | | | | >>>>>>

107722 ggaactctaagctgtcctaaagactgccacaacagg 107757

001675 V V T E L V A V T G L H V T S V E E S S 001734

>>>>>> | | | | | | | | | | | | | | | | | | | | >>>>>>

108981 gttgtcacagaacttgtagcagttactggtttacatgtgacgtctgtggaagaatcttca 109040

001735 V T I E W D L Q Q T P P D V Y A I S F K 001794

>>>>>> | | | | | | | | | | | | | | | | | | | | >>>>>>

109041 gtaactattgagtgggaccttcaacagactcccccagatgtatatgccatttcattcaaa 109100

001795 A K 001800

>>>>>> | | >>>>>>

109101 gcaaag 109106

001801 K E N G L L N N T V D G T L T S F V Q T 001860

>>>>>> | | | | | | | | | | | | | | | | | | | | >>>>>>

109341 aaggaaaatggcttattaaacaatacagtggatggcactctcacttcatttgttcagact 109400

001861 G L A S G E E Y L V S I Q P Q K G L T V 001920

>>>>>> | | | | | | | | | | | | | | | | | | | | >>>>>>

109401 ggtctggcctcaggagaggagtatcttgtgtctatacaaccccagaagggactaactgtt 109460

001921 G P E T T V T A T T S 001953

>>>>>> | | | | | | | | | | | >>>>>>

109461 gggcctgaaaccactgtaactgctactacaagt 109493

001954 I E A P L G L R V T E I T T T S F L L R 002013

>>>>>> | | | | | | | | | | | | | | | | | | | | >>>>>>

110035 attgaggctccactgggactcagagtaacagaaatcaccaccacttctttccttttaaga 110094

002014 W E R P Q S F P D R Y I V T L V S P T G 002073

>>>>>> | | | | | | | | | | | | | | | | | | | | >>>>>>

110095 tgggaacgtccccagtctttccctgatcggtacatagtgacactagtgtccccaactggg 110154

002074 K E R K L K V P G K G D R V R I T A L E 002133

>>>>>> | | | | | | | | | | | | | | | | | | | | >>>>>>

110155 aaggagagaaagctgaaagtccctggaaagggtgatagggtgaggattacagcacttgaa 110214

002134 E G T I Y K V I L R A E R G Q E Q S K G 002193

>>>>>> | | | | | | | | | | | | | | | | | | | | >>>>>>

110215 gagggaacaatttacaaagtgatattgagagcagaaaggggacaggagcaaagcaaaggg 110274

002194 M E T T A K T 002214

>>>>>> | | | | | | | >>>>>>

110275 atggagacgactgctaaaaca 110295

002215 A L D R E R K N I D D E D A R K K K G K 002274

>>>>>> | | | | | | | | | | | | | | | | | | | | >>>>>>

112936 gctcttgacagggagagaaaaaatatagatgatgaagatgcccgcaagaagaaaggaaag 112995

002275 V V T P Q Q G G Q L E K P R F S E D H Y 002334

>>>>>> | | | | | | | | | | | | | | | | | | | | >>>>>>

112996 gtagtgacccctcaacagggaggtcaacttgaaaagccacgattttcagaagatcattac 113055

002335 R P A T T G Q V L I H S G G G E G S S K 002394

>>>>>> | | | | | | | | | | | | | | | | | | | | >>>>>>

113056 cgtcctgcaacaacggggcaagttttaattcattctggaggtggagaaggcagttcaaag 113115

002395 T R E I T E Y I V E D Q T Q K G V V N T 002454

>>>>>> | | | | | | | | | | | | | | | | | | | | >>>>>>

113116 acaagggagattacagagtatattgttgaagaccaaacacaaaagggtgtggtgaacact 113175

002455 T R K T I Q T T I I T T Y H I Q N H K E 002514

>>>>>> | | | | | | | | | | | | | | | | | | | | >>>>>>

113176 actaggaagactatccagaccactattattactacataccacattcagaatcataaggaa 113235

002515 G D V V D M F E D T K S S E Y S Q T K H 002574

>>>>>> | | | | | | | | | | | | | | | | | | | | >>>>>>

113236 ggagatgttgtggatatgtttgaggatactaagagtagtgaatattcacagacaaagcat 113295

002575 L P D R K K I S S D V E G S D V G E T T 002634

>>>>>> | | | | | | | | | | | | | | | | | | | | >>>>>>

113296 ctaccagatagaaaaaaaataagcagtgatgtagagggaagtgatgtgggagagactaca 113355

002635 G Q T D I T G T I I R K H T P E K K V Q 002694

>>>>>> | | | | | | | | | | | | | | | | | | | | >>>>>>

113356 ggacagactgatataacagggactattatcagaaagcacacacctgagaagaaagtgcaa 113415

002695 S W I A R G K I I D A S S V E K P P N H 002754

>>>>>> | | | | | | | | | | | | | | | | | | | | >>>>>>

113416 agttggattgccagaggaaaaattatagatgcatcttctgtggagaaacctccaaaccat 113475

002755 L T Q K T N K T S V Q L G D T Q A A G Q 002814

>>>>>> | | | | | | | | | | | | | | | | | | | | >>>>>>

113476 cttacacagaaaactaataaaacatcagttcagttaggtgatacacaggcagctggacaa 113535

002815 Q R K K P S D K S V E V V T G I Q V N L 002874

>>>>>> | | | | | | | | | | | | | | | | | | | | >>>>>>

113536 cagaggaaaaaaccaagtgataaatctgtggaggtggtgactgggatccaagttaatcta 113595

002875 G S T E S K T F S D E K L P L T I N E K 002934

>>>>>> | | | | | | | | | | | | | | | | | | | | >>>>>>

113596 gggagtacagagtcaaaaacattttccgatgaaaaattacctttgaccataaatgagaaa 113655

002935 Q L E N L K D E R D P G S I L D D V S T 002994

>>>>>> | | | | | | | | | | | | | | | | | | | | >>>>>>

113656 cagttggagaatttaaaggatgaaagagatccaggcagtatacttgatgatgtatcaaca 113715

002995 K V H T E G R N S R K N N T G L E K K R 003054

>>>>>> | | | | | | | | | | | | | | | | | | | | >>>>>>

113716 aaagtgcatacagaaggcagaaactctagaaaaaataatacagggttggagaaaaaaagg 113775

003055 L D A G V R V Q K Q N K M E K L Y P G G 003114

>>>>>> | | | | | | | | | | | | | | | | | | | | >>>>>>

113776 ctagatgcaggtgtaagagttcagaaacaaaataaaatggaaaagttatatcctggaggt 113835

003115 L H I K A V I E N L P T K L S V Y N G T 003174

>>>>>> | | | | | | | | | | | | | | | | | | | | >>>>>>

113836 ttacacataaaagctgttattgagaacttaccaacaaaactatcagtttacaatgggacc 113895

003175 F I Q R L E S Y L R S T S Y P L R A N Q 003234

>>>>>> | | | | | | | | | | | | | | | | | | | | >>>>>>

113896 tttatacaacgtcttgaaagttacctgcgttcaacttcttacccactaagagctaatcaa 113955

003235 T V E S V A R A I F L Y L V K Y K P N S 003294

>>>>>> | | | | | | | | | | | | | | | | | | | | >>>>>>

113956 acagtggaatctgtggccagagcaattttcctttacttagtaaagtacaaacccaatagt 114015

003295 F T G M V Y D R L P Q K T P D S P E N E 003354

>>>>>> | | | | | | | | | | | | | | | | | | | | >>>>>>

114016 tttacaggaatggtatatgatcgtcttccacaaaaaactccagattcacctgaaaatgag 114075

003355 E P F G A S K I Q G N M G S V M V D N K 003414

>>>>>> | | | | | | | | | | | | | | | | | | | | >>>>>>

114076 gaaccttttggagcaagtaaaattcagggtaatatggggagtgttatggtggataataag 114135

003415 P D Q A K E V V V L R P K D R H A T M E 003474

>>>>>> | | | | | | | | | | | | | | | | | | | | >>>>>>

114136 ccagatcaagcaaaagaagtagtagttctcagaccaaaggataggcatgcaacaatggaa 114195

003475 G S Q R K V D T P A I S R Y T S S S E L 003534

>>>>>> | | | | | | | | | | | | | | | | | | | | >>>>>>

114196 ggatcacagagaaaagtagacactccagctataagtaggtacacttcttcatctgaactt 114255

003535 Y E V N M K V L K E T P T S R E E E K L 003594

>>>>>> | | | | | | | | | | | | | | | | | | | | >>>>>>

114256 tatgaagttaacatgaaagtcctgaaggagactccaacctcaagagaagaggaaaagtta 114315

003595 I S E K S T L Q R L P L K T Q L N K T Y 003654

>>>>>> | | | | | | | | | | | | | | | | | | | | >>>>>>

114316 ataagtgagaagagcaccttacaacgccttcctctaaaaactcagttaaataaaacatac 114375

003655 S E K I S D S T K R T I V P D N Q R V W 003714

>>>>>> | | | | | | | | | | | | | | | | | | | | >>>>>>

114376 tcggaaaagatttctgattctacaaaaagaactatagtacctgataatcaaagggtgtgg 114435

003715 Q A G E K D K T F T G K N K N E S L K K 003774

>>>>>> | | | | | | | | | | | | | | | | | | | | >>>>>>

114436 caagcaggtgaaaaggataagacattcactggcaaaaataagaatgaaagcttgaaaaag 114495

003775 T T V S P T T M E Y L I Y T P S V D T L 003834

>>>>>> | | | | | | | | | | | | | | | | | | | | >>>>>>

114496 actacagtgtccccaacaactatggaatatttgatctacactcctagtgtagatacattg 114555

003835 N R P T Q S S E D K T A P G V I L L T N 003894

>>>>>> | | | | | | | | | | | | | | | | | | | | >>>>>>

114556 aacagacccactcaaagctcagaagacaaaacagcacctggtgtaattttgttgactaat 114615

003895 D K G I T P K K Q P Y Y G K K V P P R P 003954

>>>>>> | | | | | | | | | | | | | | | | | | | | >>>>>>

114616 gacaaaggaattaccccgaaaaagcaaccttattatggtaaaaaagttccaccaagacca 114675

003955 E K V G S K I K E G E I K N E R L I D K 004014

>>>>>> | | | | | | | | | | | | | | | | | | | | >>>>>>

114676 gagaaagtaggatctaaaataaaggagggggagataaaaaatgaacgtctcatagataaa 114735

004015 D S R S K I L D D K A S L L Q A R E G E 004074

>>>>>> | | | | | | | | | | | | | | | | | | | | >>>>>>

114736 gattcaagaagtaagatacttgatgacaaggcatctttactgcaagcgagagagggtgag 114795

004075 E K E N E E K T I Q A L G S A G P S T H 004134

>>>>>> | | | | | | | | | | | | | | | | | | | | >>>>>>

114796 gagaaagaaaatgaagagaagactatccaagcacttgggtctgctggtccctcaacgcat 114855

004135 P T S V K I S G P G I F G R P T V V K S 004194

>>>>>> | | | | | | | | | | | | | | | | | | | | >>>>>>

114856 cctacatcagtaaaaatttctggtccaggcatttttggcagaccaactgttgtaaagagt 114915

004195 T P T N L V V S L D G L G V L L D K V M 004254

>>>>>> | | | | | | | | | | | | | | | | | | | | >>>>>>

114916 actcctacaaatcttgttgtatccctagatggtcttggggttctattggacaaggtgatg 114975

004255 I H Y R P F Q A I M T D M P Q Q L E V G 004314

>>>>>> | | | | | | | | | | | | | | | | | | | | >>>>>>

114976 attcattatcgtccatttcaagccatcatgactgatatgccacaacaactagaggttgga 115035

004315 K G V G K V V I R D L E P G T T Y R L E 004374

>>>>>> | | | | | | | | | | | | | | | | | | | | >>>>>>

115036 aaaggagttggaaaagttgtgatcagggatctagaaccagggaccacctaccgccttgaa 115095

004375 I H G L L R G Q S S K S Y I L V A D 004428

>>>>>> | | | | | | | | | | | | | | | | | | >>>>>>

115096 attcatgggctattgagggggcagtcctccaagtcctacattttggtagctgac 115149

004429 T A Q S P T I L P T E V T T K P T I S P 004488

>>>>>> | | | | | | | | | | | | | | | | | | | | >>>>>>

117429 acagctcagagtcccacaattttgcccactgaggtgaccacaaagccaacaatatcccca 117488

004489 I E D I L T T T A S S 004521

>>>>>> | | | | | | | | | | | >>>>>>

117489 atagaggacattttaactaccacggcttcttca 117521

004522 A T T R P R I P V Q M G A L Q V R N V T 004581

>>>>>> | | | | | | | | | | | | | | | | | | | | >>>>>>

117887 gctactacacggccccgcataccagttcaaatgggggcgctgcaagtgagaaatgtaaca 117946

004582 S E S I T L V W K A K I G A Y D S F L V 004641

>>>>>> | | | | | | | | | | | | | | | | | | | | >>>>>>

117947 agtgaaagtatcacattagtctggaaggccaagattggggcttatgattcattcttggtg 118006

004642 R Y D E V T D G V S P Q E L S V P G D Q 004701

>>>>>> | | | | | | | | | | | | | | | | | | | | >>>>>>

118007 cgctacgatgaggtaactgatggtgttagtccacaagaactatctgttccaggagaccaa 118066

004702 R E V T L R G L T Q D T R Y E V L L Y G 004761

>>>>>> | | | | | | | | | | | | | | | | | | | | >>>>>>

118067 cgagaggtgactttaagagggctgactcaagataccagatatgaagtattgctctatggt 118126

004762 I R E G K L T R P F K E E V T T 004809

>>>>>> | | | | | | | | | | | | | | | | >>>>>>

118127 ataagggaaggaaagctgacacggccttttaaagaagaagtaacaaca 118174

004843 M Q E E T L R G D Q T S F L I S G L V A 004902

>>>>>> | | | | | | | | | | | | | | | | | | | | >>>>>>

128028 atgcaagaggaaaccttgagaggagaccagacatctttccttatctctggactggttgca 128087

004903 A V N Y S V E L R G I W G E S Y T E P Q 004962

>>>>>> | | | | | | | | | | | | | | | | | | | | >>>>>>

128088 gcagttaattactcggttgagctgcgtggcatttggggagagagctacactgagcctcag 128147

004963 A T Y V L T 004980

>>>>>> | | | | | | >>>>>>

128148 gctacatatgtgctgaca 128165

004981 E K P Q P P R L E S L T L F D V R S E S 005040

>>>>>> | | | | | | | | | | | | | | | | | | | | >>>>>>

128936 gaaaaaccacagcctccccgcttggagtccctcactctgtttgacgtgcgcagtgaatcc 128995

005041 L N L S W D V Q G G D F D S F L L W Y R 005100

>>>>>> | | | | | | | | | | | | | | | | | | | | >>>>>>

128996 ctaaatctctcttgggatgtccagggtggagattttgattcttttctcctttggtaccgt 129055

005101 D G E G K P Q E I A L D K D L R S S T V 005160

>>>>>> | | | | | | | | | | | | | | | | | | | | >>>>>>

129056 gatggggagggaaagccacaggaaatagctcttgataaagatttgcgctcttccactgtc 129115

005161 R D L K P G K K Y K F V L Y G I S G E K 005220

>>>>>> | | | | | | | | | | | | | | | | | | | | >>>>>>

129116 agagacttgaagcctgggaaaaagtataaatttgtcttgtatggcatatcaggggaaaag 129175

005221 R S K P V T A E G 005247

>>>>>> | | | | | | | | | >>>>>>

129176 aggagcaaaccagtgacagcagagggg 129202

005248 S T D K L Q P P Q L E S L S V S D V H S 005307

>>>>>> | | | | | | | | | | | | | | | | | | | | >>>>>>

130452 tccacagacaaactacaaccaccacaactagagtctctctctgtgtctgatgtccacagt 130511

005308 D S V L L S W V V Q G G K F D S F L L N 005367

>>>>>> | | | | | | | | | | | | | | | | | | | | >>>>>>

130512 gactctgtgctactttcttgggtggttcaaggtggtaaatttgactcattccttctaaat 130571

005368 Y R D A E G K P K E A S L E G E Q R S V 005427

>>>>>> | | | | | | | | | | | | | | | | | | | | >>>>>>

130572 tatcgagacgctgagggcaaacccaaggaagcttctttggaaggggaacagcgcagtgtc 130631

005428 P V G D L K P G K K Y K F V L Y G I S G 005487

>>>>>> | | | | | | | | | | | | | | | | | | | | >>>>>>

130632 ccagttggggatctgaagcctggaaagaaatataaatttgtcctgtatgggatttcagga 130691

005488 G K K S K A A I A E T T T S 005529

>>>>>> | | | | | | | | | | | | | | >>>>>>

130692 ggaaagaaaagcaaagcagcgatagcagagaccaccacaagt 130733

005530 F L E A V G Q P S S F L N Y L F V S P R 005589

>>>>>> | | | | | | | | | | | | | | | | | | | | >>>>>>

132597 ttcctggaggccgttggtcaaccttcctctttcctgaattatctttttgtttctccacgt 132656

005590 G P H S I L V S W Q A P E G S F D S F V 005649

>>>>>> | | | | | | | | | | | | | | | | | | | | >>>>>>

132657 gggcctcattccatcttagtttcttggcaagctccagaaggatcatttgactcatttgtc 132716

005650 I R Y G I E G H E K P Q K E A S V N G T 005709

>>>>>> | | | | | | | | | | | | | | | | | | | | >>>>>>

132717 attcgttatggcattgagggtcatgagaaacctcagaaagaggcatctgtaaatggaact 132776

005710 A R T L L L A N L Q P D T P Y K V K L H 005769

>>>>>> | | | | | | | | | | | | | | | | | | | | >>>>>>

132777 gcacgtactcttttacttgctaacctccagcctgacaccccttataaagtaaaactacat 132836

005770 G V R R G K E Q G S L E T T G R T G S 005826

>>>>>> | | | | | | | | | | | | | | | | | | | >>>>>>

132837 ggtgttagaagagggaaagagcagggcagtttagaaaccactgggcgtacaggatct 132893

005827 L D L E P P Q N L R F N D V E E T S L T 005886

>>>>>> | | | | | | | | | | | | | | | | | | | | >>>>>>

133025 ttagacctggaacctcctcaaaacttaagatttaatgatgtagaagaaacatcattaact 133084

005887 V S W D P P N P E T T T F K V S Y Q L A 005946

>>>>>> | | | | | | | | | | | | | | | | | | | | >>>>>>

133085 gtcagctgggatccccccaatcctgaaactaccacattcaaagtatcatatcagcttgca 133144

005947 K G 005952

>>>>>> | | >>>>>>

133145 aagggg 133150

005953 G E P E S V N V V G D S K S L Q R L N V 006012

>>>>>> | | | | | | | | | | | | | | | | | | | | >>>>>>

133943 ggagaacctgaaagtgtaaatgtagttggagacagcaaatctcttcagagacttaatgtt 134002

006013 G T R Y E V T V V S V R G F E E S Q P L 006072

>>>>>> | | | | | | | | | | | | | | | | | | | | >>>>>>

134003 ggaactcgttatgaagttactgtggtatctgtacgagggtttgaggaaagtcagcctctg 134062

006073 T G Y I T T 006090

>>>>>> | | | | | | >>>>>>

134063 acaggctacatcactaca 134080

006091 G G G G P H S L H A H D V T E E S A L L 006150

>>>>>> | | | | | | | | | | | | | | | | | | | | >>>>>>

134448 ggtggaggtgggccacattctctgcatgcccatgatgtgactgaagaatcagcactttta 134507

006151 R W E P E P G F V D R Y V V T Y R A E N 006210

>>>>>> | | | | | | | | | | | | | | | | | | | | >>>>>>

134508 cgctgggaaccagaaccgggatttgtggacagatatgtagtgacatacagggcagagaat 134567

006211 G D Q T E L P I S G L N P H T E Y Y A S 006270

>>>>>> | | | | | | | | | | | | | | | | | | | | >>>>>>

135736 ggtgatcagactgagcttcctatctctggtctgaatccccacactgaatactatgcaagt 135795

006271 V Q S L H G S K L S S P T S T S F I T S 006330

>>>>>> | | | | | | | | | | | | | | | | | | | | >>>>>>

135796 gtgcaaagcctccatggatccaagctgagctcaccaacatctacctctttcattactagt 135855

006331 A D T P R E L L A S Q I T A Q S A L L T 006390

>>>>>> | | | | | | | | | | | | | | | | | | | | >>>>>>

136418 gctgatactccacgggaactccttgccagtcagatcactgctcagagtgctctgttgacc 136477

006391 W K P P Q V V P D G Y R L V Y Q T H D G 006450

>>>>>> | | | | | | | | | | | | | | | | | | | | >>>>>>

136478 tggaaaccacctcaggttgttcctgatgggtatcgacttgtgtatcaaacacatgatggt 136537

006451 E I K 006459

>>>>>> | | | >>>>>>

136538 gaaattaag 136546

006460 E L I L L A N L S S F T L S H L T P S T 006519

>>>>>> | | | | | | | | | | | | | | | | | | | | >>>>>>

137447 gagttaattttactagccaacttgtcctcctttaccctaagtcatctcacaccttccacc 137506

006520 P Y K V Q L H A L R G A S S S A P I S T 006579

>>>>>> | | | | | | | | | | | | | | | | | | | | >>>>>>

137507 ccatacaaagttcagcttcatgccttgcgtggtgcttccagctcggcacccatttccacc 137566

006580 S F T T 006591

>>>>>> | | | | >>>>>>

137567 tccttcactaca 137578

006592 G R I R Y P F P R D C W E K H M N G D L 006651

>>>>>> | | | | | | | | | | | | | | | | | | | | >>>>>>

139135 ggtcgaataagatatccatttccccgagattgctgggaaaagcatatgaatggagacttg 139194

006652 Q S G V F T I Y L G G S K D D P L L V Y 006711

>>>>>> | | | | | | | | | | | | | | | | | | | | >>>>>>

139195 cagagtggtgtgttcacaatatatctgggaggatcaaaagatgatccattgctggtgtac 139254

006712 C D M E T D G G G W I 006744

>>>>>> | | | | | | | | | | | >>>>>>

139255 tgtgacatggagactgatggaggtggctggata 139287

006745 V F Q R R I D G R T D F W R N W R Q Y K 006804

>>>>>> | | | | | | | | | | | | | | | | | | | | >>>>>>

140405 gtttttcagaggaggatagatgggagaactgatttttggagaaactggaggcaatataag 140464

006805 E G F G N L T S E F W L 006840

>>>>>> | | | | | | | | | | | | >>>>>>

140465 gaggggtttggtaaccttacttcagagttctggctg 140500

006841 G N I A L H R L S S L A P Y E L R V D L 006900

>>>>>> | | | | | | | | | | | | | | | | | | | | >>>>>>

140806 ggaaacattgctctgcatcgtctatcatctttggcaccatatgagttacgtgtagacctc 140865

006901 R A G A E A A Y A V Y E D F R V E G E D 006960

>>>>>> | | | | | | | | | | | | | | | | | | | | >>>>>>

140866 cgggcaggagctgaggctgcctatgctgtgtatgaagattttcgtgttgagggtgaagac 140925

006961 K H F R L R I G A Y R G N A 007002

>>>>>> | | | | | | | | | | | | | | >>>>>>

140926 aaacactttaggctgcggataggagcatacagaggaaatgca 140967

007003 G D S L S Y H N N M I F S T R D R D A E 007062

>>>>>> | | | | | | | | | | | | | | | | | | | | >>>>>>

141111 ggggactctctgagctatcacaacaatatgattttttcaacacgggaccgtgatgcagag 141170

007063 K R I L P C S I S Y R G A W W Y K N C H 007122

>>>>>> | | | | | | | | | | | | | | | | | | | | >>>>>>

141171 aaaagaatattaccttgttccatttcttatcgtggagcttggtggtataagaactgccac 141230

007123 Y A N L N G M Y G N N K D H 007164

>>>>>> | | | | | | | | | | | | | | >>>>>>

141231 tatgccaatcttaatgggatgtatggcaacaacaaagaccat 141272

007165 Q G V N W H T W K G F E F S I P F T E M 007224

>>>>>> | | | | | | | | | | | | | | | | | | | | >>>>>>

141358 cagggtgtgaactggcacacatggaaaggcttcgaattttcgattccattcactgagatg 141417

007225 K M R P Q R T G N W R R L 007263

>>>>>> | | | | | | | | | | | | | >>>>>>

141418 aagatgcggccacagagaacaggcaactggcgccgtctt 141456

Xenopus tenascin-R (partial):

0000001 L D P P M N L T A T E V A R T S A L I S 0000060

<<<<<<< | | | | | | | | | | | | | | | | | | | | <<<<<<<

1806838 ctggatccacccatgaatctgactgccacagaagtggcaaggactagtgcgttaatttct 1806779

0000061 W Q P P I S P I E N Y I L T Y K S A N E 0000120

<<<<<<< | | | | | | | | | | | | | | | | | | | | <<<<<<<

1806778 tggcagccacccatctctcctattgaaaattacatcctcacttataaatctgctaatgag 1806719

0000121 S R K 0000129

<<<<<<< | | | <<<<<<<

1806718 agccggaag 1806710

0000130 E L I V D A E D T W I R L E G L L E S T 0000189

<<<<<<< | | | | | | | | | | | | | | | | | | | | <<<<<<<

1806526 gagctgatagtagatgctgaggacacatggatccgcctggaaggcttactggagagtaca 1806467

0000190 E Y T V S I L S V Q N G E R S S V T H T 0000249

<<<<<<< | | | | | | | | | | | | | | | | | | | | <<<<<<<

1806466 gaatacactgtgagcattctgtctgtgcagaatggggagcggagctcggtcacccacact 1806407

0000250 V F T T 0000261

<<<<<<< | | | | <<<<<<<

1806406 gtattcactaca 1806395

0000262 G G R V F S Y P Q D C A Q H M L N G D N 0000321

<<<<<<< | | | | | | | | | | | | | | | | | | | | <<<<<<<

1804547 ggagggagggtgttttcctacccacaagactgtgctcagcacatgctgaatggagacaac 1804488

0000322 Q S G V Y Y I Y I N G D M S Q S V P V Y 0000381

<<<<<<< | | | | | | | | | | | | | | | | | | | | <<<<<<<

1804487 cagagtggagtatattatatttatatcaatggtgacatgagccaaagtgtccctgtatac 1804428

0000382 C D M A T D A G G W I 0000414

<<<<<<< | | | | | | | | | | | <<<<<<<

1804427 tgtgacatggctactgatgcaggaggctggatt 1804395

0000415 V F Q R R Q N G L T D F F R K W A D Y R 0000474

<<<<<<< | | | | | | | | | | | | | | | | | | | | <<<<<<<

1803363 gtatttcaaagacgccagaatggattgacagattttttccgcaagtgggcagattatcgt 1803304

0000475 V G F G N L E D E F W L 0000510

<<<<<<< | | | | | | | | | | | | <<<<<<<

1803303 gtaggttttgggaacctggaggatgagttttggctg 1803268

0000511 G L D T L H Q V T S Q G R Y E L R I D M 0000570

<<<<<<< | | | | | | | | | | | | | | | | | | | | <<<<<<<

1802869 ggacttgacactcttcatcaggtgacctctcaggggcgctacgaactgcgaattgatatg 1802810

0000571 R D G Q E A V Y A Y Y N K F N I G D A R 0000630

<<<<<<< | | | | | | | | | | | | | | | | | | | | <<<<<<<

1802809 cgggatggacaagaagcagtctatgcatattataataagttcaacattggcgatgcccga 1802750

0000631 S L Y K L R I G D F N G T S 0000672

<<<<<<< | | | | | | | | | | | | | | <<<<<<<

1802749 agtttatacaagctgcgtatcggggacttcaatgggacatca 1802708

0000673 G D S L T Y H Q G R P F S T K D R D N D 0000732

<<<<<<< | | | | | | | | | | | | | | | | | | | | <<<<<<<

1802544 ggtgattcactcacttaccaccagggacgaccattctctacaaaggatagagacaatgat 1802485

0000733 V A V T N C A S S Y K G A W W Y K N C H 0000792

<<<<<<< | | | | | | | | | | | | | | | | | | | | <<<<<<<

1802484 gttgctgtaacaaattgtgcctcttcatataaaggagcatggtggtataagaactgtcac 1802425

0000793 R T N L N G K Y G E S R H S 0000834

<<<<<<< | | | | | | | | | | | | | | <<<<<<<

1802424 cgtacaaacctgaatggcaaatatggtgaatcccggcacagc 1802383

0000835 Q F N N I P T I L Q K T A Y 0000876

<<<<<<< | | | | | | | | | | | | | | <<<<<<<

1800233 cagttcaataacattcctaccatattgcagaaaactgcatac 1800192
